# Oleuropein Activates Neonatal Neocortical Proteasomes, but Proteasome Gene Targeting by AAV9 Is Variable in a Clinically Relevant Piglet Model of Brain Hypoxia-Ischemia and Hypothermia

**DOI:** 10.3390/cells10082120

**Published:** 2021-08-18

**Authors:** Nagat El Demerdash, May W. Chen, Caitlin E. O’Brien, Shawn Adams, Ewa Kulikowicz, Lee J. Martin, Jennifer K. Lee

**Affiliations:** 1 Department of Anesthesiology and Critical Care Medicine, Johns Hopkins University, Baltimore, MD 21287, USA; neldeme1@jhmi.edu (N.E.D.); cobrie19@jhmi.edu (C.E.O.); shawnmadams99@gmail.com (S.A.); ekuliko1@jhmi.edu (E.K.); 2 Department of Pediatrics, School of Medicine, Johns Hopkins University, Baltimore, MD 21287, USA; maywchen@jhmi.edu; 3 Department of Pathology, Johns Hopkins University, Baltimore, MD 21287, USA; martinl@jhmi.edu

**Keywords:** neonatal brain damage, hypoxia-ischemia, white matter injury, proteinopathy, conditional gene manipulation, hypothermia, newborn, proteasome, adeno-associated virus

## Abstract

Cerebral hypoxia-ischemia (HI) compromises the proteasome in a clinically relevant neonatal piglet model. Protecting and activating proteasomes could be an adjunct therapy to hypothermia. We investigated whether chymotrypsin-like proteasome activity differs regionally and developmentally in the neonatal brain. We also tested whether neonatal brain proteasomes can be modulated by oleuropein, an experimental pleiotropic neuroprotective drug, or by targeting a proteasome subunit gene using recombinant adeno-associated virus-9 (AAV). During post-HI hypothermia, we treated piglets with oleuropein, used AAV-short hairpin RNA (shRNA) to knock down proteasome activator 28γ (PA28γ), or enforced PA28γ using AAV-PA28γ with green fluorescent protein (GFP). Neonatal neocortex and subcortical white matter had greater proteasome activity than did liver and kidney. Neonatal white matter had higher proteasome activity than did juvenile white matter. Lower arterial pH 1 h after HI correlated with greater subsequent cortical proteasome activity. With increasing brain homogenate protein input into the assay, the initial proteasome activity increased only among shams, whereas HI increased total kinetic proteasome activity. OLE increased the initial neocortical proteasome activity after hypothermia. AAV drove GFP expression, and white matter PA28γ levels correlated with proteasome activity and subunit levels. However, AAV proteasome modulation varied. Thus, neonatal neocortical proteasomes can be pharmacologically activated. HI slows the initial proteasome performance, but then augments ongoing catalytic activity. AAV-mediated genetic manipulation in the piglet brain holds promise, though proteasome gene targeting requires further development.

## 1. Introduction

Neonatal hypoxic-ischemic encephalopathy (HIE) from birth asphyxia causes nearly one million infant deaths worldwide each year [1,2]. Therapeutic hypothermia reduces the risk of death [3], but approximately a third of survivors who receive hypothermia have moderate-to-severe impairments in cognitive and executive function 6–7 years later [4,5]. Proteinopathy is signatory for hypothermia-resistant brain injury from hypoxia-ischemia (HI), notably in the white matter, in a well-established and clinically relevant neonatal piglet model [6]. Failure to clear damaged proteins could be cytotoxic to neural cells, though the causality is uncertain [7,8,9,10]. Proteasomes normally degrade proteins damaged by oxidative stress. We found proteasome insufficiency and carbonylated and ubiquitinated protein accumulation in neonatal piglets that received hypothermia after HI. Higher levels of these damaged proteins were directly associated with myelin injury [6]. Because the proteasome response to HI and hypothermia may differ between cerebral cortex and white matter [6], understanding regional proteasome dysfunction could be pivotal for treating HI brain injury. Stress responses are compromised in humans after birth asphyxia, including a depletion in hypoxia-inducible factor 1α [11].

Choosing an appropriate preclinical model to test brain proteasomes in vivo is critical because longer-lived species have greater proteasome activity than do short-lived species, such as mice and rats [12]. Swine proteasomes are likely to be clinically relevant because pigs live for 10–20 years and they have gyrencephalic brains with large white matter volume, similar to that of humans [13]. Human and pig proteasomes also interact similarly with proteins [14], whereas human and mouse proteasomes process peptides differently [15]. Therefore, we explored oleuropein (OLE), a small molecule botanical compound that has proteasome-activating properties in cell culture [16], as a potential therapeutic for piglet HI. Our piglet HI model has salient physiologic and neuropathologic features that are similar to those of human neonatal HIE [17,18,19]. Piglet models [19,20,21,22] contributed to the successful translation of therapeutic hypothermia, which is the main clinical therapy for neonatal HIE [4]. Previously, we found that OLE protects the white matter after HI and during hypothermia in piglets [23]. However, we do not know whether the OLE-mediated white matter protection was related to proteasomes because OLE is pleiotropic and also modulates autophagy [24], inflammation [25], and oxidative stress [25,26].

This study was designed to characterize proteasomes in neonatal piglet tissues and to test whether systemically administering OLE after HI and during hypothermia, the standard of care for neonatal HIE [4], can activate brain proteasomes. We studied a piglet cohort that previously showed reduced HI white matter injury with OLE treatment [23]. In a parallel setting with hypothermia, we also explored the feasibility of virus-mediated manipulation of the proteasome activator 28γ (PA28γ) gene. PA28γ is a known regulatory subunit that stimulates the proteasome 20S (P20S) core particle to degrade damaged peptides [27]. We hypothesized that proteasome activity differs in the neocortex, white matter, liver, and kidney; that brain proteasome activity differs developmentally; that OLE activates proteasomes after HI and hypothermia; and that adeno-associated virus 9 (AAV) can genetically modulate the proteasome during hypothermia in the neonatal piglet brain. To our knowledge, the efficacy of AAV-mediated genetic manipulation has not been studied in a clinically relevant, neonatal, large animal HI model. Approaches to specifically modulate the proteasome in the neonatal gyrencephalic brain could isolate the proteasome’s identity as a therapeutic target in HIE.

## 2. Methods

**Figure 1:**
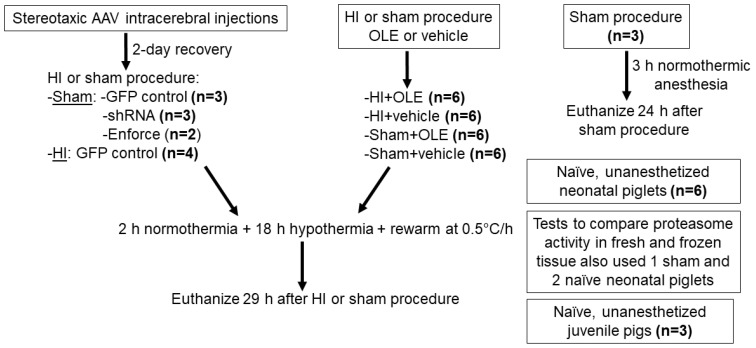
Study design and number of piglets in each experimental group. AAV, adeno-associated virus serotype 9; GFP, green fluorescent protein; HI, hypoxia-ischemia; OLE, oleuropein; shRNA, short hairpin RNA for proteasome subunit gene knockdown; enforce, proteasome subunit gene overexpression. Not included here is a small group of HI piglets recovered with hypothermia (n = 2) or normothermia (n = 2) without OLE or vehicle treatments that were used for pilot proteomic studies.

The Johns Hopkins University Animal Care and Use Committee approved all protocols (research protocol SW20M201, approved 14 July 2020), which were conducted in compliance with the United States Public Health Service Policy on the Humane Care and Use of Laboratory Animals and the Guide for the Care and Use of Laboratory Animals. Our protocols and reporting also followed the National Institutes of Health and the Animal Research: Reporting in Vivo Experiments guidelines. Animal comfort was ensured at all times. To conserve animals, we measured proteasome activity in tissue that had been collected from 24 piglets (Sus scrofa) used in a previously reported study and stored at −80 °C [23]. These conditions do not significantly affect proteasome activity (Supplemental Figure S1). We conducted experiments in 24 additional neonatal piglets. Figure 1 shows the study design. We also harvested tissue from three juvenile pigs (approximately 30 kg, males) that did not receive any anesthesia or surgery and were reported in another study [28]. All neonatal piglets were male, 2–4 days old, and approximately 1.0–2.2 kg. Pigs were delivered to the laboratory from a local farm (Archer Farms, Inc., Darlington, MD, USA). The primary endpoints were proteasome activity and subunit levels. Secondary endpoints were markers for protein ubiquitination, the oxidative stress response, and cell death measured by western blot.

### 2.1. Anesthesia

For all experiments, we anesthetized the piglets with 5% isoflurane and 50%/50% nitrous oxide/oxygen through a nose cone before intubation. Mechanical ventilation maintained normocarbia. We decreased the isoflurane to 1–2% and increased the nitrous oxide to 70% in 30% oxygen for placement of a femoral arterial and external jugular venous catheters. Finally, we administered a 20 µg/kg fentanyl bolus and delivered 20 µg/kg/h intravenous (IV) fentanyl and IV normal saline with 5% dextrose at 4 mL/kg/h.

### 2.2. Genetic Manipulation of the Neonatal Piglet Brain Using AAV9

Once anesthetized, the piglets were positioned in a stereotaxic head frame, and the skull was secured in a flat position with ear bars, a mouthpiece, and snout clamp. Bilateral cranial burr holes (2–3 mm in diameter) were drilled at the coordinates 14 mm, 8 mm, and 2 mm anterior to the bregma along the coronal suture and 7.5 mm lateral to the midline. These coordinates target the somatosensory cortex and underlying subcortical white matter. This cortical region is vulnerable to HI in neonatal piglet [17]. A sterile needle was secured by a micromanipulator and slowly advanced 7.5 mm ventral to the dura to deliver the viruses into the brain parenchyma.

Custom-designed recombinant AAVs were engineered (Vector Biosystems, Inc., Malvern, PA, USA) to target proteasome subunit PA28γ (PSME3). All viruses were serotype AAV9 for clinical relevance [29]. To knockdown PA28γ, we used an AAV (AAV9-eGFP-U6-hPSME3-shRNA) expressing enhanced green fluorescent protein (GFP) and human short hairpin RNA (shRNA) driven by the U6 promoter that controls RNA expression. The shRNA targeting sequence that recognizes human PSME3 mRNA variant 1 is: CGTGACAGATTGATGAGAACTCGAGTTCTCATCAATCTCTGTCACGTTTTT. Humans and pigs have a 100% identical sequence for PMSE3 (https://www.uniprot.org/uniprot/P61291, accessed on 4 January 2021) [30]. The Internal Ribosome Entry site (IRES) drove bicistronic dual expression of two transgenes. To enforce PA28γ expression, we used AAV9-CMV-hPSME3-IRES-eGFP. The recombinant AAV9 was designed to express the 765 base pair open reading frame of human PMSE3 (Entrez gene ID 10197, transcript ID NM_005782.4). AAV-CMV-eGFP was used as a control virus.

Piglets received AAV doses of 2 × 10^10^–2 × 10^11^ genome copies (gc; 20 µL), 4 × 10^10^–4 × 10^11^ gc (40 µL), or 5 × 10^10^–5 × 10^11^ gc (50 µL) in each cerebral hemisphere. We added F108 40 ng and polybrene 2.5 µg (an additional 2.5 µL) to some AAV injections to test whether these adjuvants enhance transduction in piglet brain [31]. The hemispheric AAV dose was divided among the three unilateral injection sites. For each cranial burr hole, approximately one-quarter of the AAV volume was injected at the deepest location (7.5 mm ventral to dura) followed by a 5-min needle dwell time. Then, the needle was withdrawn a few millimeters for the next injection and 5-min dwell time. These steps were repeated until all AAV was delivered. We used slow needle penetrations to minimize injury above that of the needle track. Piglets emerged from anesthesia, recovered for 2 days with free access to milk, and then underwent a second anesthetic for hypothermia after HI or sham procedure. We tested AAV transduction in the presence of hypothermia, because this is the standard of clinical care for HIE [4,32,33].

### 2.3. HI and Hypothermia

We have reported our HI and hypothermia protocol in detail [6,23,34,35,36]. After the piglets were anesthetized, the isoflurane was discontinued. For the AAV piglets that were re-anesthetized, the venous catheter was re-accessed. All piglets received vecuronium (0.2 mg/kg/h, IV) to prevent ventilatory effort during the HI protocol and to prevent shivering during hypothermia. Dopamine was started when needed to maintain the mean arterial blood pressure (MAP) above 45 mmHg, which approximates the lower limit of autoregulation for hypothermic neonatal piglets [37]. This anesthetic regimen does not cause cortical apoptosis [35] or oligodendrocyte or myelin injury [23,34], nor does it affect the unfolded protein response [38] or P20S levels [6] in the piglet brain.

The piglets were randomized to sham + OLE, sham + vehicle, HI + OLE, or HI + vehicle treatments. We also performed the HI and sham procedures in piglets with intracerebral AAV to test the feasibility of virus transduction under both conditions (Figure 1). For a pilot proteomic study, we used HI piglets recovered under hypothermic or normothermic temperatures for 29 h. For the HI protocol, the inspired oxygen concentration was decreased to 10% to achieve a goal oxyhemoglobin saturation of 30–35% for 45 min. Then, 5 min of room air was supplied to briefly reoxygenate the heart, a step that is required for cardiac resuscitation in this model. Asphyxia was subsequently induced by clamping the endotracheal tube for 8 min. Piglets were resuscitated with 50% oxygen, chest compressions, and 100 µg/kg epinephrine IV. Ventricular fibrillation was treated with 10 J/kg biphasic defibrillation [39]. Piglets that required more than 3 min of chest compressions for return of spontaneous circulation were excluded. Then, the oxygen was decreased to 30% for the remainder of the experiment. Piglets that underwent the sham procedure received the same anesthetic and catheter placement, with 30% oxygen throughout the protocol. We measured arterial blood gases and electrolyte levels every 1–4 h. Sodium bicarbonate was administered as needed for metabolic acidosis when the base excess was −6 or lower.

OLE ((2*S*,3*E*,4*S*)-3-Ethylidene-2-(β-D-glucopyranosyloxy)-3,4-dihydro-5-(methoxycarbonyl)-2*H*-pyran-4-acetic acid 2-(3,4-dihydroxyphenyl) ethyl ester, CAS number 32619-424, catalog number 12247, Sigma Aldrich, St. Louis, MO, USA) 2.7 mg/kg IV in 1% dimethylsulfoxide (DMSO, in saline) was administered 15 min after resuscitation from HI or time equivalent in the sham procedure. Subsequent OLE doses of 0.7 mg/kg IV were administered 1 h after HI and again every 2 h until the experiment ended. Piglets that were randomized to receive vehicle were administered 1% DMSO in equal volumes and dose timing. Piglets with intracerebral AAV did not receive OLE or vehicle.

Piglets that received OLE, vehicle, or AAV received overnight hypothermia and rewarming. After resuscitation from HI or time equivalent in shams, normothermia was maintained for 2 h at a goal rectal temperature of 38.0–39.5 °C, which is normothermic for neonatal swine [40,41]. Whole-body hypothermia was then induced by applying ice packs and cooling blankets to a goal of 34.0 °C, which mimics the 4 °C decrease of clinical hypothermia for HIE (37 °C human normothermia with cooling to ~33 °C) [32]. We delayed the induction of hypothermia by 2 h to simulate the cooling delay that occurs clinically [42]. After 18 h of hypothermia (20 h after resuscitation from HI), rewarming at 0.5 °C/h was initiated with heat lamps and warming blankets to achieve piglet normothermia at 29 h. This rewarming rate is used clinically for the treatment of HIE [32]. The piglets were euthanized 29 h after HI or time equivalent in shams. We studied this subacute survival time because proteasome P20S immunoreactivity becomes depleted in white matter oligodendrocytes [6] and OLE can rescue oligodendrocytes and myelin 29 h after HI [23].

### 2.4. Proteasome Evaluation in Brain, Liver, and Kidney

Separate neonatal piglets had sham anesthesia induced as described above for intubation and mechanical ventilation. After placing external jugular venous and arterial catheters, we administered fentanyl and discontinued the isoflurane. The pigs received 3 h of normothermic anesthesia with 70%/30% nitrous oxide/oxygen and fentanyl. After they had recovered from anesthesia for 24 h, we euthanized them to compare proteasome activity levels in the cerebral cortex, white matter, liver, and kidney.

### 2.5. Tissue Harvesting

All pigs were deeply anesthetized and euthanized at their designated times with 50 mg/kg phenobarbital and 6.4 mg/kg phenytoin administered IV. Freshly prepared ice-cold phosphate-buffered saline was transcardially perfused for exsanguination. The brain was rapidly removed from the skull, transected in the midsagittal plane, and placed onto ice. Each hemisphere was cut into slabs. The somatosensory cortex and subcortical white matter were dissected, and tissue samples were immediately flash frozen in cold (−70 °C) isopentane chilled by dry ice. Pure white matter samples were obtained by using a 2-mm micropunch [23]. We analyzed the somatosensory cortex and its subcortical white matter because these regions show highly reproducible vulnerability to HI in piglets [43], and human infants with HIE have similar peri-Rolandic vulnerability [32,44].

### 2.6. Proteasome Activity Assay

We used a commercial proteasome activity assay kit with the fluorogenic substrate succinyl-Leu-Leu-Val-Tyr-7-amido-4-methylcoumarin (Suc-LLVY-AMC) to detect chymotrypsin-like activity (Ubiquitin-Proteasome Biotechnologies, LLC, Dallas, TX, USA; catalog number J4110, purchased in 2020–2021). This AMC-tagged peptide is a P20S β5 subunit substrate that is cleaved to liberate AMC for fluorescence detection [45]. The P20S is the core protease subunit that degrades oxidized and ubiquitinated proteins [8]. Unfixed, fresh non-frozen or fresh-frozen samples of somatosensory cortex and subcortical white matter were homogenized in cold tissue lysis buffer (40 mM Tris and 50 mM NaCl in distilled water at pH 7.2 with 10% glycerol, 2 mM β-mercaptoethanol [Sigma Aldrich], 5 mM MgCl_2_, and 2 mM ATP [Ubiquitin-Proteasome Biotechnologies, LLC]). After the homogenate was centrifuged for 30 min at 4 °C, the supernatant was collected and kept on ice for protein measurements using the Pierce BCA protein assay kit (Thermo Scientific, Carlsbad, CA, USA). The proteasome assay buffer was prepared by diluting 20× proteasome assay buffer to 1× in sterile water at 37 °C. The substrate was then prepared by adding 2 µL of the 1000× Suc-LLVY-AMC stock to the assay buffer.

All samples were tested in duplicate in 96-well, black-bottom plates. Each plate included AMC dilutions (blank, 1:500, 1:1000, 1:5000, and 1:10,000) to generate a concentration-dependent AMC fluorescence standard curve. We added 50 µL of Suc-LLVY-AMC (50 µM final concentration) dissolved in reaction buffer and 50 µL of reaction buffer to each well followed by 25 µg, 50 µg, or 100 µg (maximum sample input was ~1.2 µL) of cortical or white matter protein sample. We added the proteasome inhibitor MG132 at 100 µM to a parallel set of wells as a negative control to isolate proteasome-specific chymotrypsin-like activity from the overall catalytic activities of non-proteasome serine proteases. As a positive control, we used an HEK293 cell lysate (Ubiquitin-Proteasome Biotechnologies, LLC) because these cells are a standard test input for proteasome activity [46].

AMC fluorescence from Suc-LLVY-AMC peptide cleavage was measured immediately by a plate reader for 15 min of assay reaction time. Each sample’s AMC fluorescence was determined by the AMC standard curve and is reported as relative fluorescence units. In addition to the proteasome, Suc-LLVY-AMC is also degraded by calpains and other chymotrypsin-like proteases. Adding the proteasome inhibitor MG132 isolates and distinguishes the proteasome substrate cleavage from non-proteasome proteolysis. Calculating the difference between AMC fluorescence in tissue with MG132 and that of tissue without MG132 established the relative fluorescence levels specific to proteasome Suc-LLVY-AMC peptidolysis. Proteasome activity levels were measured by AMC relative fluorescence from 0 to 15 min of assay reaction time. The investigator who conducted the proteasome activity assay was blinded to the treatment group.

### 2.7. Western Blot Analysis

We used our published western blot protocol [6,38] to study proteasome subunit protein levels in the same brain homogenates (aliquots were spiked with protease inhibitors) that we tested for the proteasome assay. For piglets that received HI or sham procedure followed by OLE or vehicle, only the left side of the brain was analyzed. The HI protocol causes global injury, and laterality is not expected. Both cerebral hemispheres were analyzed in piglets that received AAV. An HEK293 cell lysate was loaded into each gel as an inter-gel standard and proteasome subunit control because the proteasome β5 and α3 subunit identities have been confirmed by transient transfection in HEK293 cells [47]. GFP protein (Biovision, Milpitas, CA, USA) was loaded into gels for the AAV experiments as a positive control.

We studied two P20S proteasome subunits and a regulatory subunit. The P20S β5 subunit is the known chymotrypsin-like catalytic subunit for SUC-LLVY-AMC [48]. The P20S α3 subunit is a cornerstone structural subunit that interacts with many different proteins and functions as a putative core particle gatekeeper [49]. The P20S α3 subunit also has a role in RNA metabolism, cell death, and cell cycle control [50,51], which could be important to oligodendrocytes in immature white matter after HI [23,34]. Proteasome subunit PA28γ activates P20S in brain cells [27,52]. We additionally measured caspase-7 to assess cortical cell death [53], the antioxidant enzyme manganese superoxide dismutase (SOD2) [54], and ubiquitinated protein levels.

Ponceau S staining (Sigma Life Science, St. Louis, MO, USA) was used to confirm uniform electroelution transfer to the nitrocellulose membrane and to quantify protein loading in each lane. After being blocked with 5% nonfat milk, the membranes were incubated with one of the following primary antibodies: polyclonal GFP IgG (1:1500, rabbit, BioVision, Milpitas, CA, Research Resource Identifier [RRID] AB_222261), monoclonal P20S subunit α3 IgG (1:1000, rabbit, clone 12446, Cell Signaling, Danvers, MA, RRID AB_2797918), polyclonal P20S subunit β5 IgG (1:1000, rabbit, GeneTex, Irvine, CA, RRID AB_385014), polyclonal PA28γ IgG (1:1000, rabbit, Cell Signaling, RRID AB_10695726), monoclonal cleaved caspase-7 IgG (1:1000, rabbit, Cell Signaling, RRID:AB_11178377), polyclonal SOD2 IgG (1:1500, rabbit, Enzo, RRID:AB_10616816), or monoclonal ubiquitin IgG (1:1000, mouse, Abcam, RRID:AB_305802).

The membranes were then washed and incubated with an anti-rabbit secondary antibody (1:5000, IgG-horseradish peroxidase, Jackson ImmunoResearch, West Grove, PA, USA) or anti-mouse secondary antibody (1:5000, IgG-horseradish peroxidase, Jackson ImmunoResearch). All full-length western blots used in this study for each antibody and their corresponding Ponceau S-stained membranes are shown in the Supplemental Western Blot Common Data Element file. Chemiluminescent detection (ThermoFisher, San Francisco, CA, USA) enabled us to visualize the proteins using a ChemiDoc imaging system (BioRad, Hercules, CA, USA). Target protein band densities were quantified by ImageJ (National Institutes of Health, Bethesda, MD, USA), and each band was normalized to (divided by) the Ponceau protein load. Each piglet’s target protein band density was also divided by that of the common HEK293 cell lysate to allow for between-gel comparisons. Because the HEK293 cell lysate is negative for cleaved caspase-7 and could not be used as an inter-gel standard, all caspase-7 western blot membranes were exposed to identical solutions and underwent identical durations of chemiluminescent detection. The investigators who analyzed the western blots were blinded to the treatment group.

### 2.8. Proteome-Wide Profiling of Protein Oxidative Damage in Neonatal HI Piglet White Matter by Liquid Chromatography-Tandem Mass Spectroscopy (LC-MS/MS)

Freshly frozen subcortical white matters samples from HI-hypothermia piglets (n = 2) and HI-normothermia piglets (n = 2), generated from a prior study [6], were subjected to dinitrophenylhydrazone (DNP) derivatization to tag carbonyl modified protein amino acids using the Oxyblot protein oxidation detection kit (Millipore, Burlington, MA, USA) as described (6). The samples were then fractionated by SDS-PAGE using 4–12% Tris-glycine gels. Protein size fractionation was verified by flanking lanes with Precision Plus Protein Dual Color Standards (Bio-Rad, Hercules, CA, USA). Using the molecular standards as a guide, regions were excised in the gels containing the 20–30 kDa protein sizes. Negative control lanes were also sampled. We used a biased sampling design, because in prior work, we have found the accumulation of carbonyl-modified proteins within this size range [6].

Gel slices were cut into smaller pieces in Eppendorf tubes using a scalpel blade (number 11) and incubated overnight on a rotating plate in elution buffer (50 mM Tris, pH 7.9, 0.1 mM EDTA, 1 mM dithiothreitol [DTT], 0.15 M NaCl, and 0.1% SDS). The mixture was then centrifuged at maximum speed to pellet gel fragments and separate the supernatant. Four volumes of cold acetone (−20 °C) were added to the protein eluate. Proteins were allowed to precipitate in a dry-ice-ethanol slurry. The precipitated protein was washed three times in ethanol-ethyl acetate (1:1) and then dissolved in 50 mM ammonium bicarbonate buffer for enzymatic digestion. The proteins were subjected to trypsin digestion (1:200, porcine pancreas, sequencing grade, ThermoFisher) in 25 mM ammonium bicarbonate (pH 8.5) for 8 h at 37 °C. This approach was an optimization in the balance of amino acid sequence coverage in peptide fragments and missed cleavages. Peptides were reduced with 20 mM DTT for 30 min, alkylated with 100 mM iodoacetamide in the dark for 1 h, acidified with 0.5% trifluroacetic acid, reverse-phase filtered, vacuum-dried, and reconstituted in acetonitrile/formic acid. Peptides were subjected to LC-MS/MS at the JHU Proteomic Core Facility using Linear Trap Quadrupole (LTQ) Orbitrap Velos MS (ThermoFisher Scientific, Waltham, MA) at 2.0 kV, operating in a data-dependent Fourier transform-ion trap (FT-IT) parallel acquisition mode for a “shotgun” optimization and normalization of collision energy.

Tandem mass spectra were extracted without charge state deconvolution and deisotoping. Protein digestion efficiency by trypsin was verified qualitatively by assessing the presence or absence of an intact peak for cytochrome *c* detected in the chromatogram. All MS/MS samples were analyzed using Mascot (Matrix Science, London, UK; version 2.5.1). Mascot was set to search the SusScrofa 2015_Pig_20170112 database (https://www.uniprot.org/proteomes/UP000008227, accessed on 7 August 2021, 49,792 entries containing manual and computational annotations). Mascot was searched with a fragment ion mass tolerance of 0.0100 Da (monoisotopic) and a parent ion tolerance of 5.0 PPM (monoisotopic). Carbamidomethyl of cysteine (+57) was specified in Mascot as a fixed modification. The variable modification was oxidation. Arginine (+137), threonine (+178), lysine (+179), and proline (+194) carbonylation detected by DNP were specified in Mascot as the variable amino acid residue modifications. The maximum missed cleavage was set at 3.

Scaffold (version Scaffold_5.0.1, Proteome Software Inc., Portland, OR, USA) was used to validate MS/MS based peptide and protein identifications. Peptide identifications were accepted if they could be established at greater than 95.0% probability by the Scaffold Local False Detection Rate (FDR) algorithm utilizing decoy matches. Protein identifications were accepted if they could be established at greater than 95.0% probability and contained at least one identified peptide. Protein probabilities were assigned by the Protein Prophet algorithm [55]. Proteins that contained similar peptides and could not be differentiated based on MS/MS analysis alone were grouped to satisfy the principles of parsimony.

### 2.9. Statistical Analysis

We used GraphPad v.8.0.0 (GraphPad Software, San Diego, CA, USA) to generate graphs and analyze the data. In naïve unanesthetized piglets, we compared proteasome activity (relative fluorescence levels) across the 15-min assay in the cortex and white matter with and without the proteasome inhibitor MG132 using repeated measures two-way analysis of variance (ANOVA) and post-hoc Tukey’s tests. These proteasome activity data passed Shapiro–Wilk tests of normality.

We used linear regression to analyze the slope of proteasome activity over assay reaction time and the starting fluorescence plate read (t_0_) with 25, 50, and 100 µg of assay protein input among the treatment groups (sham + vehicle, sham + OLE, HI + vehicle, HI + OLE, and naïve). We stratified the data by cortex and white matter and confirmed that the data passed the Shapiro–Wilk tests of normality.

The data for 1-h pH and 1-h MAP were compared to the slope of proteasome activity over assay reaction time from the 100-µg protein assay load using Pearson correlations if the data passed the Shapiro–Wilk tests of normality or Spearman correlations for non-normal data.

Western blot data among naïve, HI, and sham piglets that received OLE or vehicle were analyzed by a Mann–Whitney test or Kruskal–Wallis ANOVA on ranks. Mann–Whitney tests compared proteasome activity between fresh and frozen tissue. For piglets that received AAV, PA28γ levels were compared to proteasome subunit levels, the slope of proteasome activity over assay reaction time, and the t_0_ proteasome activity using Spearman correlations in data normalized to (divided by) GFP western blot levels. Proteasome activity data in the brain regions, liver, and kidney were descriptively evaluated for comparisons between neonatal and juvenile pigs and between neonatal piglets with and without AAV injection.

### 2.10. Power Calculations

We found no information on proteasome chymotrypsin-like activity in neonatal brain regions. We conducted a power calculation using somatosensory cortex and subcortical white matter of three naïve unanesthetized piglets using PS Power and Sample Size Calculations v 3.0 (biostat.mc.vanderbilt.edu/PowerSampleSize). The mean difference in peak proteasome activity between the paired brain regions was 1367 (within-group standard deviation [SD]: 476). A sample size of six piglets would have a power of >0.9 to reject the null hypothesis of no regional difference at an alpha level of 0.05 and Cohen’s *d* of 1.7, which indicates a large potential effect size [56].

Because OLE’s effects on proteasome activity in HI neonatal brain have not been well studied, we estimated the power from the cortical proteasome activity of three HI + vehicle and three HI + OLE piglets. The mean difference in the peak proteasome activity was 2840 with a within-group SD of 1388. Six piglets per group would provide power of 0.9 to reject the null hypothesis of no OLE effect at an alpha level of 0.05 with Cohen’s *d* of 2 for a large potential effect size [56].

## 3. Results

Forty-five neonatal piglets and three juvenile pigs were studied. Additionally, two naïve piglets and one sham piglet that received 3 h of normothermic anesthesia were euthanized to compare proteasome activity levels in fresh and frozen tissue (Supplemental Figure S1). Six naïve, unanesthetized piglets had brain tissue harvested for proteasome activity assays and western blots. Three piglets that underwent sham procedure, 3 h of anesthesia, and 24 h of normothermic recovery were used to compare brain, liver, and kidney proteasome activity. Eight piglets had stereotaxic intracerebral AAV injections (n = 3 with AAV-GFP; n = 3 with AAV-shRNA to PA28γ-GFP; and n = 2 with AAV-PA28γ-GFP) followed by sham procedure, 18 h of hypothermia, and 9 h of rewarming. Four other piglets received AAV-GFP followed by HI, hypothermia, and rewarming. We tested hypothermia with rewarming to mimic clinical therapeutic hypothermia for HIE [32]. All piglets that received AAV or 3 h of anesthesia only to compare proteasome activity among different organs completed their protocols. Among piglets randomized to HI or sham procedure and OLE or vehicle, three HI + vehicle piglets died during overnight hypothermia and were excluded from the study.

For the 24 piglets used in our prior study of HI and OLE, their physiologic data were reported previously [23]. Briefly, the hypoxia protocol reduced the oxyhemoglobin saturation to approximately 30% at 42 min. The saturations further decreased to approximately 6% at 7 min of asphyxia. All piglets recovered to have oxyhemoglobin saturations of 97% or higher after resuscitation.

### 3.1. Proteasome Activity and Subunit Levels Are Enriched in Neonatal, Uninjured Neocortex and White Matter

**Figure 2:**
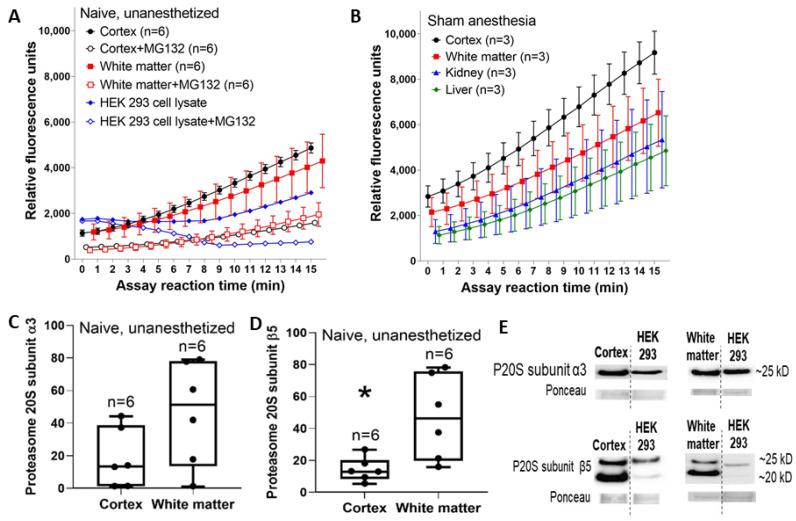
Reaction progress curves for proteasome activity in somatosensory cortex and subcortical white matter of uninjured neonatal piglets. (**A**) The proteasome inhibitor MG132 reduced fluorescence in the cortex and white matter independently (*p* = 0.005) and interactively with assay time (*p <* 0.001). In post-hoc comparisons, MG132 decreased the fluorescence levels in the cortex (*p* < 0.001) and white matter (*p* < 0.001). Proteasome activity did not differ between the cortex and white matter in the presence or absence of MG132 (*p* > 0.05 for both). An HEK293 cell lysate with and without MG132 treatment is shown for a control comparison. (**B**) Separate piglets received 3 h of normothermic sham anesthesia and 24-h recovery. The cortex had the highest proteasome activity followed by the white matter. The liver and kidney had lower activity than either brain region. Data are shown as means with standard errors of the means. (**C**,**D**) Western blot analysis showed that naïve cortex and white matter had similar proteasome 20S subunit α3 levels (C, *p* = 0.094), but immunoreactivity for proteasome 20S subunit β5 was higher in white matter than in the cortex (* *p* = 0.015). Between-gel comparisons were made by dividing each band’s immunoreactivity optical density by the density of a common HEK293 cell lysate that was loaded into every gel. In panels (**C**,**D**), each circle represents one piglet and the box plot whiskers are 5–95th percentiles. (**E**) Representative western blots of the naïve, unanesthetized piglet brain. Bands from the cortex and the HEK293 cell lysate are shown from the same gel with the same exposure time. Separate gels have bands from white matter and the HEK293 cell lysate. Molecular weights are indicated.

We first characterized proteasome-related chymotrypsin-like activity within the somatosensory cortex and subcortical white matter of naïve, unanesthetized piglets. Relative fluorescence levels differed among brain regions with and without the proteasome inhibitor MG132 (cortex, cortex + MG132, white matter, and white matter + MG132; *p* = 0.005, n = 6) and interacted with time over the total kinetic assay (*p <* 0.001). Post-hoc comparisons showed that MG132 inhibited proteasome activity in the cortex (*p <* 0.001, mean difference: 1805, 95% confidence interval [CI]: 1454, 2156) and white matter (*p <* 0.001, mean difference: 1518, 95% CI: 934, 2103). MG132 suppressed mean chymotrypsin-like activity by 67% in the cortex and by 55% in white matter at 15 min into the reaction. Cortical and white matter activity did not differ (*p* > 0.05 without MG132; *p* > 0.05 with MG132; Figure 2A). Western blots showed that P20S subunit β5 immunoreactivity was higher in white matter than in the cortex of naïve piglets (*p* = 0.015; n = 6), whereas P20S subunit α3 immunoreactivity did not differ (*p* = 0.094; Figure 2C–E).

In piglets that received 3 h of normothermic anesthesia, sham procedure, and 24-h recovery, proteasome activity was highest in the cortex followed by white matter (Figure 2B). The initial fluorescence readings at t_0_ differed among the tissue regions. The liver and kidney had lower activities than did either brain region.

**Figure 3:**
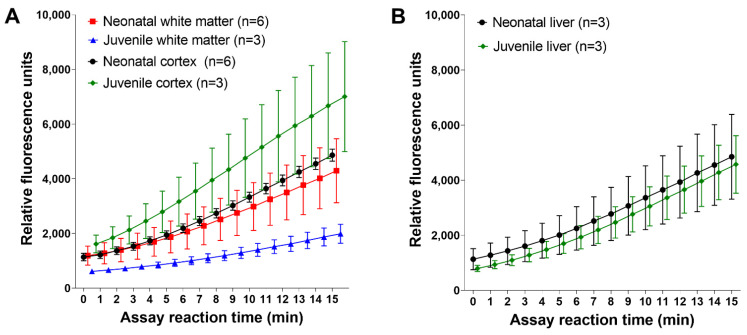
Proteasome activity reaction progress curves in the somatosensory cortex and subcortical white matter of naïve and unanesthetized neonatal and juvenile pigs. (**A**) In the cortex, juvenile pigs had greater mean proteasome activity levels than did neonatal pigs. The white matter showed the opposite relationship, with neonatal piglets having greater white matter proteasome activity than juvenile pigs. Juvenile white matter had the lowest activity of all groups (the neonatal white matter and neonatal cortex data are the same as the data shown in Figure 2A). (**B**) Proteasome activity in the liver was similar in neonatal and juvenile pigs. Data are shown as means with standard errors of the means.

Next, we compared naïve and unanesthetized neonatal and juvenile piglets. Proteasome activity in subcortical white matter was lower in juvenile pigs than in neonatal pigs (Figure 3A). It was also lower than proteasome activity in either the juvenile or neonatal cortex. The neonatal and juvenile liver had similar activities (Figure 3B).

### 3.2. Proteasome Activity and Subunit Levels after HI, OLE, and Hypothermia

**Figure 4:**
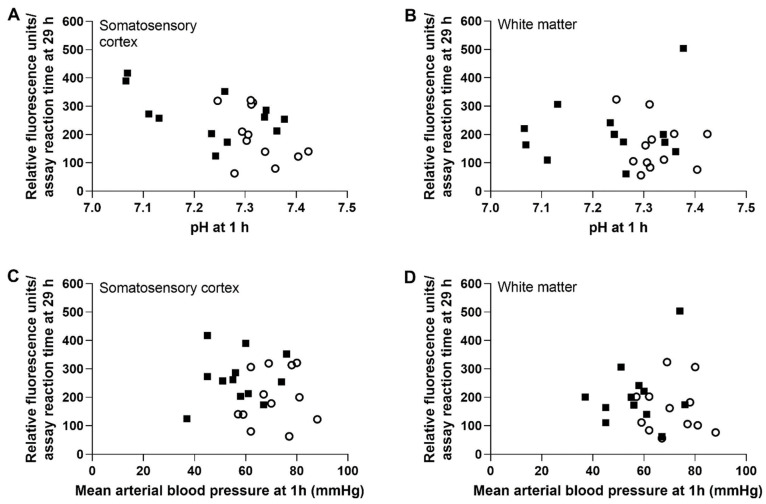
Correlation between the arterial pH and mean arterial blood pressure (MAP) 1 h after resuscitation and the slope of proteasome activity over the 15-min assay reaction time 29 h after resuscitation. HI piglets are represented by solid squares. Shams are represented by open circles. (**A**,**B**) Lower pH correlated with higher subsequent proteasome activity in the somatosensory cortex (r = −0.538; 95% CI: −0.773, −0.171; *p* = 0.007, n = 24, (**A**) but not in the white matter. (**C**,**D**) Early MAP was not related to future proteasome activity in either brain region (n = 24). All piglets received hypothermia and rewarming.

Early blood gas physiology after HI was associated with brain proteasome activity 1 day later in piglets that received HI or sham procedure, OLE or vehicle, and hypothermia. Lower pH measured 1 h after resuscitation or time equivalent in shams correlated with higher cortical proteasome activity at 29 h (r = −0.538; 95% CI: −0.773, −0.171; *p* = 0.007, n = 24). However, the 1-h pH was not related to the white matter’s 29-h proteasome activity (r = −0.092, *p* = 0.668). Early MAP was also unrelated to the future proteasome activity in the cortex (r = −0.101, *p* = 0.638) and white matter (r = −0.188, *p* = 0.380; Figure 4).

**Figure 5:**
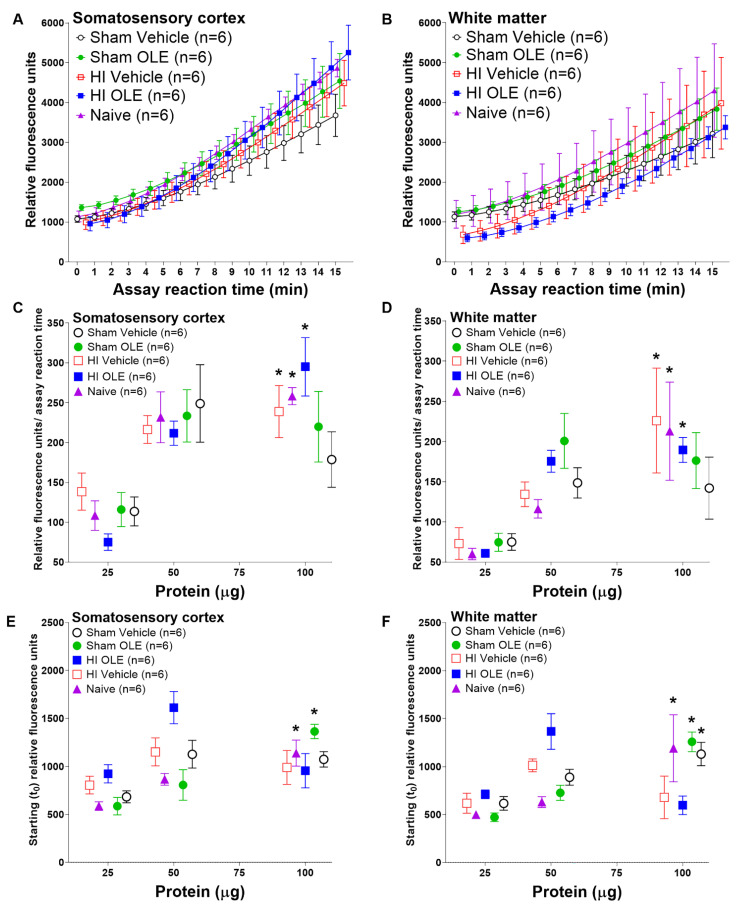
Proteasome activity in piglets randomized to hypoxia-ischemia (HI) or sham procedure, and oleuropein (OLE) or vehicle. All groups except for the naïve unanesthetized control group received hypothermia. (**A**,**B**) Example assay reaction data in somatosensory cortex and white matter with 100 µg protein in the fluorescence proteasome activity assay. (**C**,**D**) The slope of proteasome activity over assay reaction time increased in the cortex and white matter with greater assay protein input among HI OLE (cortex: *p* < 0.001; white matter: *p <* 0.001), HI vehicle (cortex: *p* = 0.023; white matter: *p* = 0.013), and naïve piglets (cortex: *p* = 0.002; white matter: *p* = 0.007). (**E**) In the cortex, the starting (t_0_) proteasome activity increased with greater protein input in sham OLE (*p <* 0.001) and naïve piglets (*p <* 0.001). (**F**) The white matter’s t_0_ proteasome activity increased with higher protein input in sham OLE (*p <* 0.001), sham vehicle (*p* = 0.002), and naïve groups (*p* = 0.021). Data are shown as means with standard errors of the means. * *p <* 0.05.

We profiled proteasome activity relative to homogenate protein input into the assay, assuming proportionally higher amounts of proteasome with greater input, using constant substrate and saturating ATP concentrations. Proteasome activity differed by piglet treatment group and by the amount of protein loaded into the proteasome activity assay. Figure 5A,B show examples of proteasome activity across the assay’s reaction time in the cortex and white matter with 100 µg of protein in the assay. We then examined proteasome activity across different protein amounts loaded into the assay.

The slope of proteasome activity over assay reaction time increased with greater amounts of cortical and white matter protein in the assay for piglets that received HI + OLE (cortex: *p* < 0.001, β = 2.753, 95% CI: 1.724, 3.781; white matter: *p <* 0.001, β = 1.512, 95% CI: 0.781, 2.243) or HI + vehicle (cortex: *p* = 0.023, β = 1.214, 95% CI: 0.190, 2.24; white matter: *p* = 0.013, β = 2.011, 95% CI: 0.476, 3.55; Figure 5C,D). Naïve piglets also showed greater proteasome activity with more protein (cortex: *p* = 0.002, β = 1.789, 95% CI: 0.767, 2.81; white matter: *p* = 0.007, β = 2.023, 95% CI: 0.650, 3.40). There were no differences between the HI + OLE, HI + vehicle, and naïve groups. Sham piglets, whether treated with OLE or vehicle, did not show a change in the proteasome activity slope with incrementally higher protein loads to drive the reaction (*p* > 0.05 for cortex and white matter).

Piglet treatment also affected the starting proteasome activity readout at t_0_. The cortical t_0_ proteasome activity level was higher with greater protein input in sham OLE (*p* < 0.001, β = 10.47, 95% CI: 6.098, 14.84) and naïve piglets (*p <* 0.001, β = 7.081; 95% CI: 3.608, 10.55; Figure 5E). The other treatment groups showed no effect on t_0_ activity from higher cortical proteins levels (*p* > 0.05). In white matter, the t_0_ proteasome activity increased with more protein among sham OLE (*p* < 0.001, β = 10.50, 95% CI: 7.512, 13.48), sham vehicle (*p* = 0.002, β = 6.567, 95% CI: 2.909, 10.23), and naïve piglets (*p* = 0.021, β = 9.501, 95% CI: 1.658, 17.34; Figure 5F). HI with neither OLE nor vehicle showed no change in white matter t_0_ proteasome activity (*p* > 0.05).

**Figure 6:**
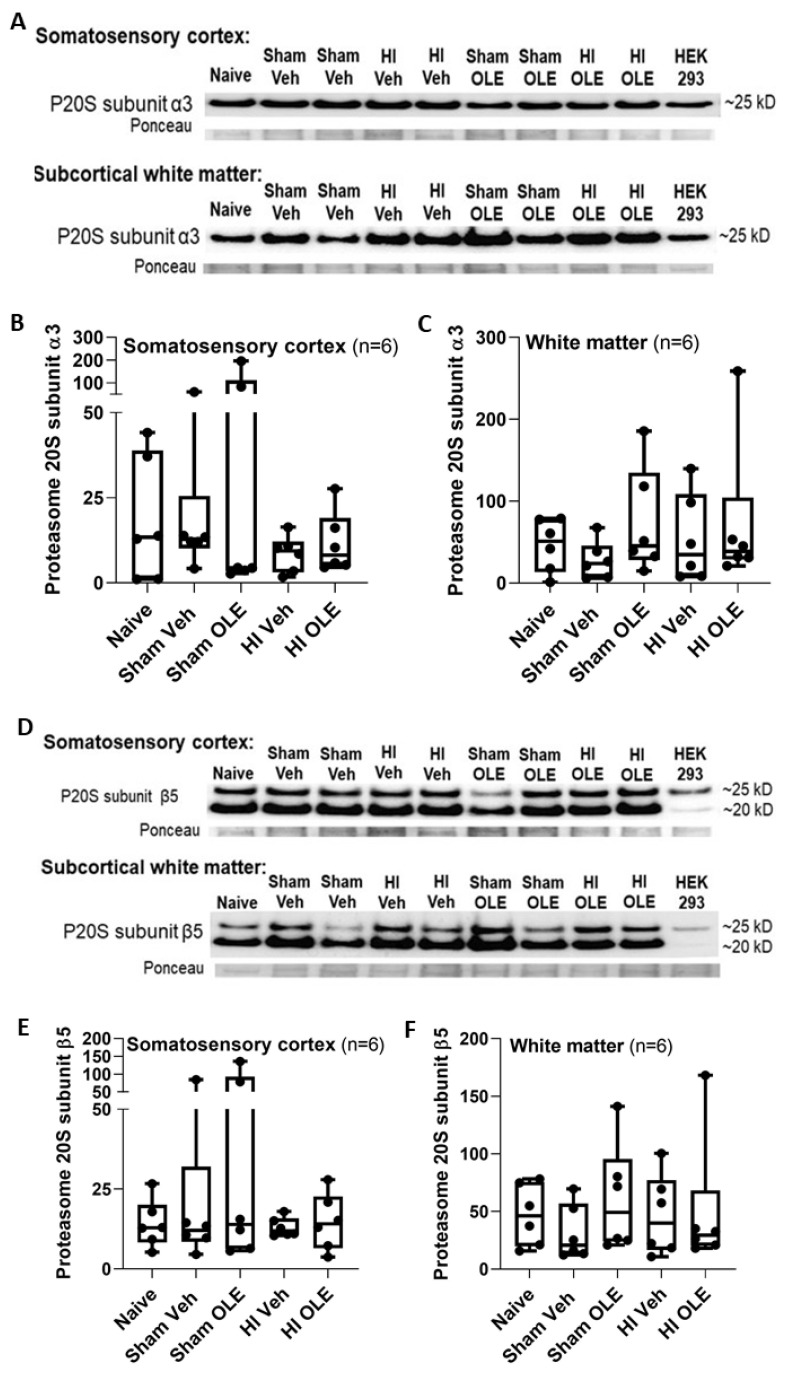
Western blotting was used to measure proteasome 20S (P20S) subunit immunoreactivity levels in piglets that received hypoxia-ischemia (HI) or sham procedure with oleuropein (OLE) or vehicle (Veh). Naïve, unanesthetized piglets were an additional control group. (**A**–**C**) P20S subunit α3 levels did not differ in the cortex (*p* = 0.806) or white matter (*p* = 0.594). (**D**–**F**) P20S subunit β5 levels also were not different in the cortex (*p* = 0.986) or white matter (*p* = 0.564). Ponceau S-stained membranes show protein loading (**A**,**D**). The box plot whiskers show the 5th and 95th percentiles.

Proteasome activity slope and t_0_ did not differ between freshly harvested tissue and tissue frozen at –80 °C for 3.5 years to 2 h in naïve neonatal piglets (cortex: *p >* 0.999 for slope and *p* = 0.898 for t_0_; white matter: *p* = 0.240 for slope and *p* = 0.797 for t_0_) (Supplemental Figure S1). Western blot analysis of the two P20S subunits showed that treatment did not affect α3 or β5 immunoreactivity levels in the cortex (α3: *p* = 0.806; β5: *p* = 0.986; n = 6) or white matter (α3: *p* = 0.594; β5: *p* = 0.564) (Figure 6).

### 3.3. Conditional Transgenic Manipulation in the Neonatal Piglet Brain

**Figure 7:**
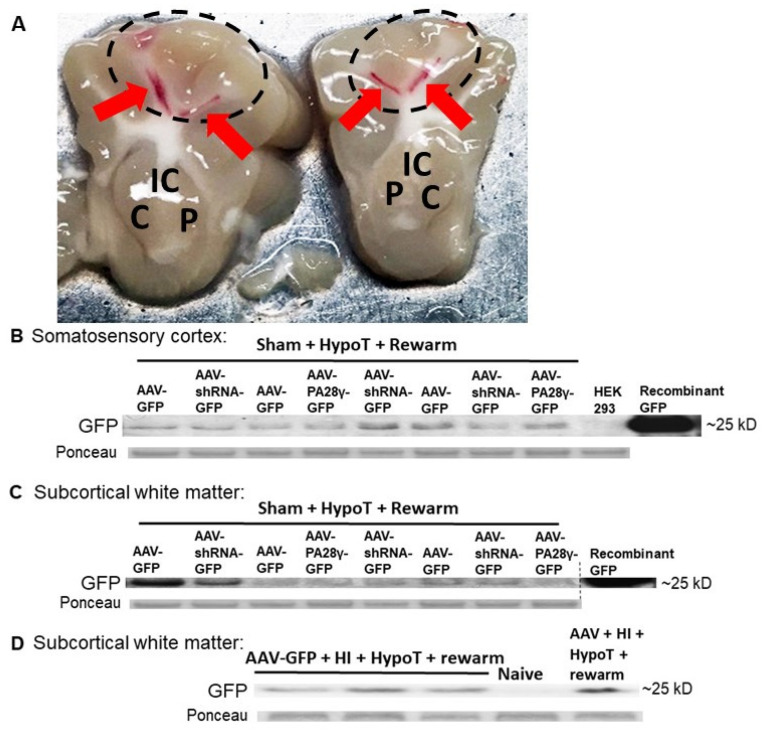
Western blots for green fluorescent protein (GFP) immunoreactivity confirmed adeno-associated virus (AAV) infectivity and transgene expression in piglets that underwent sham procedure and hypothermia or hypoxia-ischemia (HI) and hypothermia. (**A**) Examples of stereotaxically guided injection sites (red arrows) are shown in the subcortical white matter of the somatosensory gyri (dashed ovals). C, caudate; IC, internal capsule; P, putamen. (**B**,**C**) Piglets received stereotaxic intracerebral injections of AAV-GFP, AAV-shRNA to PA28γ-GFP, or AAV-PA28γ-GFP followed by sham procedure and hypothermia (HypoT). GFP protein levels confirmed virus transduction in somatosensory cortex (**B**) and white matter (**C**). A non-transfected HEK293 cell lysate served as a negative control, and recombinant GFP was a positive control. (**D**) To test whether cerebral AAV transduction is possible after piglet HI and hypothermia, we administered stereotaxic AAV-GFP injections to separate piglets followed by HI and hypothermia. GFP levels confirmed AAV transduction. A naïve piglet without AAV showed no GFP immunoreactivity. The approximate molecular weight, which was confirmed by recombinant GFP, is noted. Ponceau S-stained membranes show protein loading.

We tested the feasibility of genetically manipulating the neonatal piglet brain using conditional targeting techniques. Western blots revealed successful AAV transduction as GFP was detected in the somatosensory cortex and subcortical white matter of piglets that received AAV-GFP, AAV-shRNA to PA28γ-GFP, or AAV-PA28γ-GFP followed by sham procedure and hypothermia (Figure 7A–C). The molecular weight for virally expressed GFP was confirmed by purified recombinant GFP, and a non-transfected HEK293 cell lysate control confirmed immunoreactive band identity by the absence of GFP immunoreactivity. Separate piglets that received AAV, HI, and hypothermia also had AAV transduction with GFP immunoreactivity in the subcortical white matter. A naïve control piglet showed no GFP (Figure 7D).

**Figure 8:**
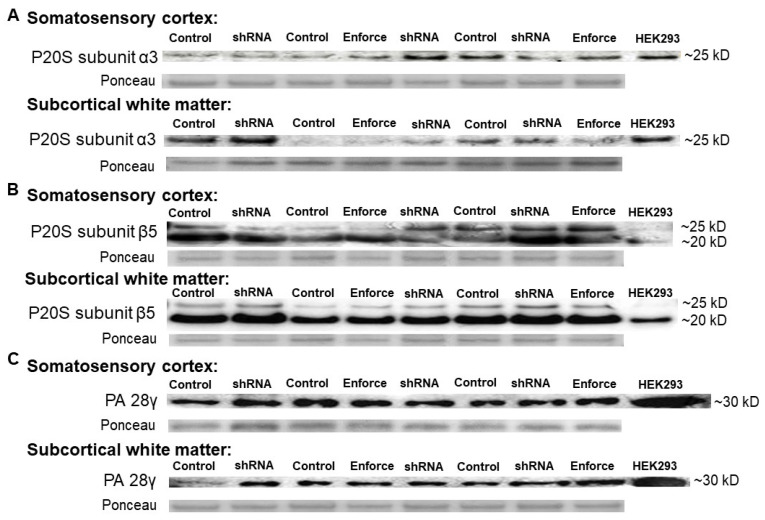
Western blotting for the proteasome 20S (P20S) subunits α3 (**A**), β5 (**B**), and PA28γ (**C**) in the piglet somatosensory cortex and subcortical white matter after intracerebral injections of adeno-associated virus (AAV). The different injections were AAV-green fluorescent protein (GFP, control), AAV with shRNA to PA28γ-GFP, or AAV-PA28γ-GFP (enforce). A non-transfected HEK293 cell lysate provided an additional control. Ponceau S-stained membranes show protein loading.

**Figure 9:**
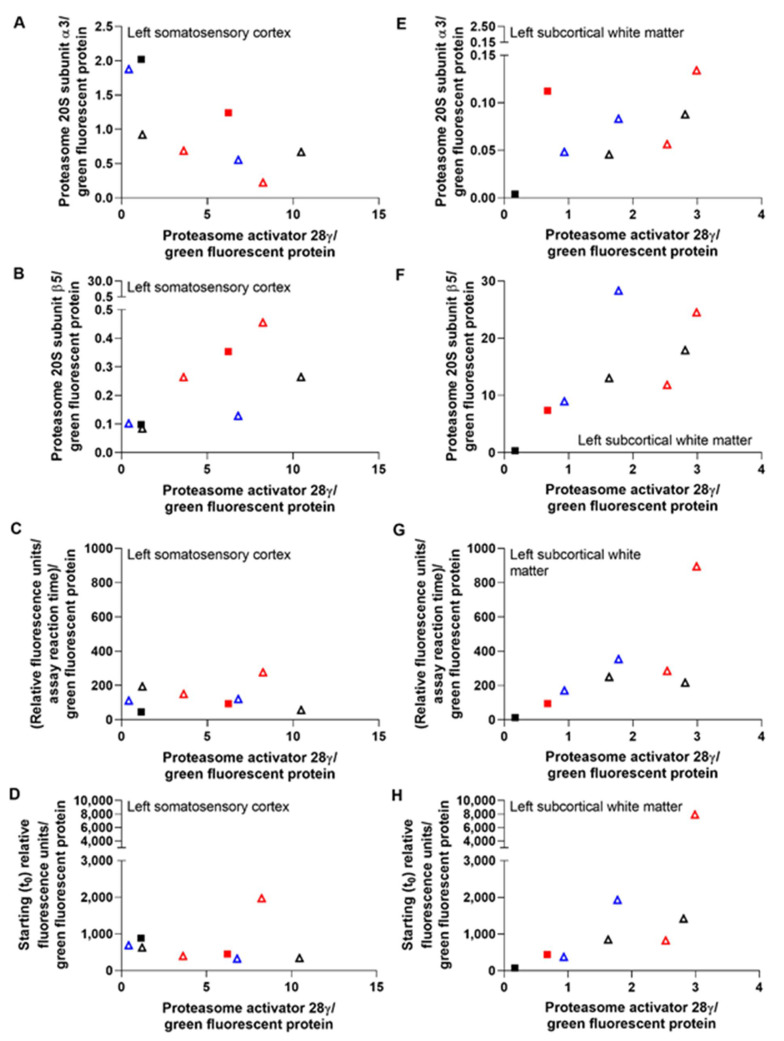
Correlations between immunoreactivity for PA28γ and proteasome 20S subunits α3 and β5 and between PA28γ and proteasome activity in piglets that received left intracerebral adeno-associated virus (AAV). Data from the somatosensory cortex (**A**–**D**) and subcortical white matter (**E**–**H**) are shown. PA28γ and α3 levels had a negative correlation in the cortex (r = −0.833, *p* = 0.015, **A**) but no relationship in white matter (r = 0.595, *p* = 0.132, **E**). PA28γ correlated positively with β5 in the cortex (r = 0.738, *p* = 0.046, **B**) and white matter (r = 0.810, *p* = 0.022, **F**). The PA28γ level also correlated with the slope of white matter proteasome activity over assay reaction time (r = 0.833, *p* = 0.015, **G**) and starting t_0_ activity (r = 0.857, *p* = 0.011, **H**). This relationship did not occur in the cortex (slope: r = 0.143, *p* = 0.752, **C**; t_0_: r = −0.405, *p* = 0.327, **D**). The shapes of the data points indicate the cerebral hemispheric AAV dose: square = 2×10^10^–2×10^11^ genome copies (gc) and triangle = 5×10^10^–5×10^11^ gc. Black: AAV-GFP control, red: AAV-shRNA to PA28γ-GFP, and blue AAV-PA28γ-GFP (enforce). Piglets represented by an open symbol also received the adjuvant (40 ng of F108 and 2.5 µg of polybrene).

P20S subunit immunoreactivity levels are shown by western blot in Figure 8. Co-migrating bands in the HEK293 cell lysate lanes verified the proteasome subunit immunoreactive band identities in piglet brain. The left cerebral hemisphere, which received the F108 and polybrene adjuvant and overall higher AAV doses, showed correlations between PA28γ, the P20S subunits α3 and β5, and proteasome activity. PA28γ negatively correlated with α3 subunit levels in the cortex (r = −0.833, *p* = 0.015) but not in the white matter (r = 0.595, *p* = 0.132; Figure 9A,E). PA28γ positively correlated with β5 in both the cortex (r = 0.738, *p* = 0.046) and white matter (r = 0.810, *p* = 0.022; Figure 9B,F). PA28γ was also related to the slope of proteasome activity over assay reaction time in white matter (r = 0.833, *p* = 0.015; Figure 9G) but not cortex (slope: r = 0.143, *p* = 0.752; Figure 9C). The starting t_0_ proteasome activity and PA28γ were also correlated in white matter (r = 0.857, *p* = 0.011, Figure 9H) but not cortex (r = −0.405, *p* = 0.327, Figure 9D). In the right hemisphere, which received overall lower AAV doses and no adjuvant, PA28γ was not related to subunit α3 or β5 levels or to proteasome activity slope or t_0_ (*p* > 0.05 for all comparisons; Supplemental Figure S2).

**Figure 10:**
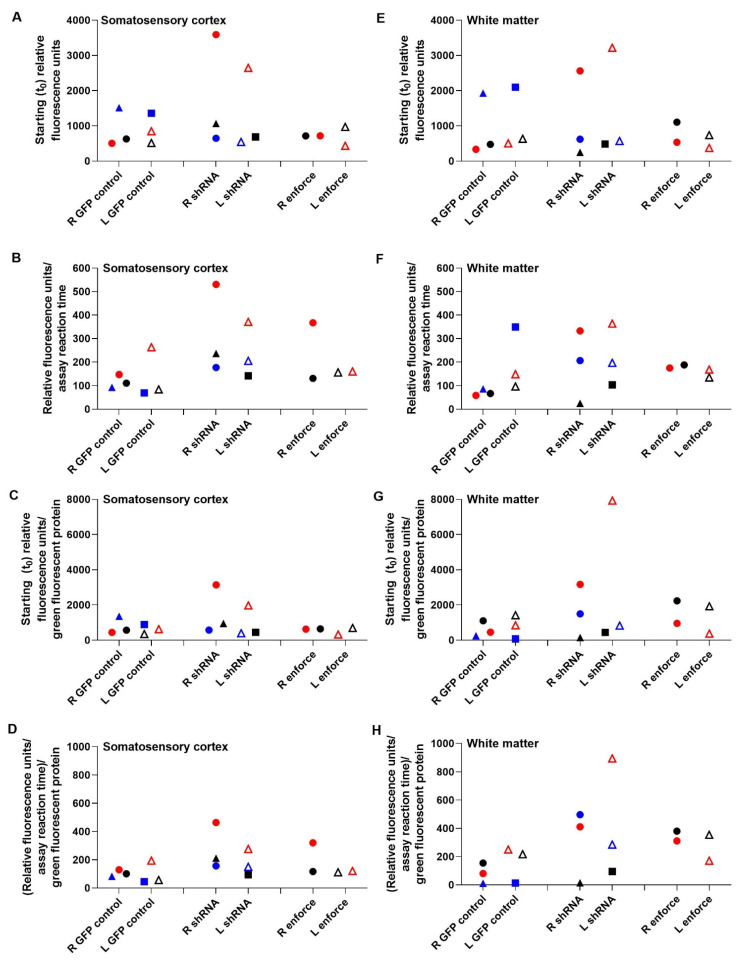
Proteasome activity in piglets that received intracerebral adeno-associated virus (AAV)-green fluorescent protein (GFP), AAV-shRNA to PA28γ, or AAV-PA28γ-GFP followed by sham procedure and hypothermia. Starting proteasome activity at t_0_ and the slope of proteasome activity over the assay reaction time are shown in the right (R) and left (L) somatosensory cortex (**A**–**D**) and subcortical white matter (**E**–**H**). Data in panels (**C**,**D**,**G**,**H**) are normalized to (divided by) the GFP immunoreactivity level from the piglet’s western blot as an indicator of virus transduction. The AAV-shRNA to PA28γ-GFP did not knock down proteasome activity, and AAV-PA28γ-GFP did not increase proteasome activity relative to AAV-GFP. Some piglets that received AAV-PA28γ shRNA-GFP had paradoxical increases in proteasome activity. R- and L-sided data from the same piglet are matched by color within each treatment group. The shapes of the data points indicate the cerebral hemispheric AAV dose: square = 2 × 10^10^–2 × 10^11^ genome copies (gc), circle = 4 × 10^10^–4 × 10^11^ gc, and triangle = 5 × 10^10^–5 × 10^11^ gc. Piglets represented by an open symbol also received 40 ng of F108 and 2.5 µg of polybrene.

**Figure 11:**
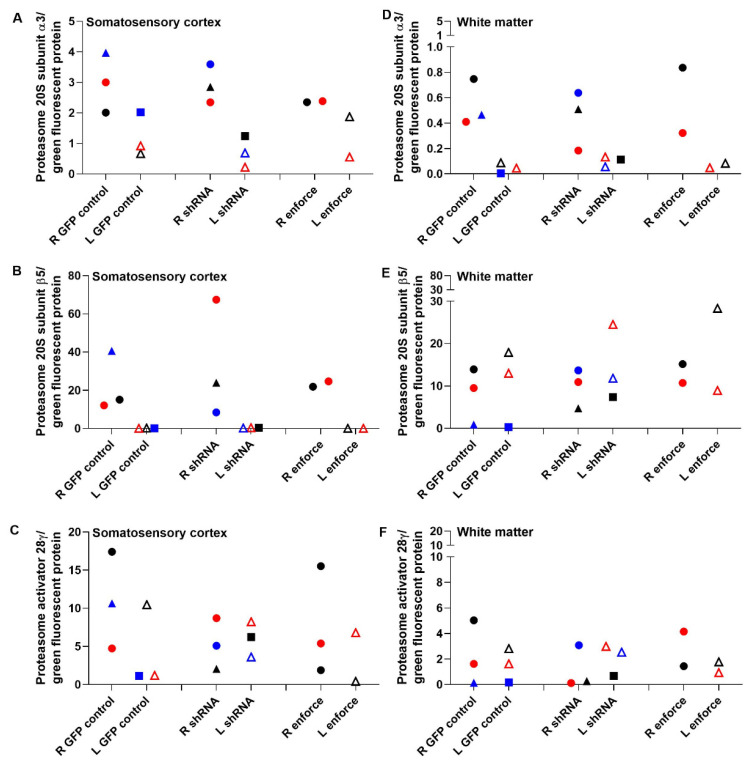
Western blot data for proteasome 20S subunits in piglets that received intracerebral adeno-associated virus (AAV)-green fluorescent protein (GFP), AAV-shRNA to PA28γ-GFP, or AAV-PA28γ-GFP and sham procedure with hypothermia. Data are normalized to (divided by) the piglet’s GFP level as a measure of AAV transduction in the right (R) and left (L) somatosensory cortex (**A**–**C**) and subcortical white matter (**D**–**F**). AAV-shRNA to PA28γ-GFP did not consistently decrease PA28γ levels or alter the levels of α3 and β5 subunits compared to AAV-GFP. AAV-PA28γ-GFP did not reliably raise the subunit levels. Data points from the same piglet are matched by color within each treatment group. The shapes indicate the AAV dose in each cerebral hemisphere: square = 2 × 10^10^–2 × 10^11^ genome copies (gc), circle = 4 × 10^10^–4 × 10^11^ gc, and triangle = 5 × 10^10^–5 × 10^11^ gc. Open symbols indicate piglets that also received adjuvant (40 ng of F108 and 2.5 µg of polybrene). Data were divided by a Ponceau-stained protein loading control.

The proteasome activity assay did not show cortical or white matter proteasome upregulation by AAV-PA28γ-GFP or inhibition by AAV-shRNA to PA28γ-GFP relative to that in AAV-GFP-treated piglets. Different AAV doses and the addition of the F108 and polybrene adjuvant did not enhance proteasome activity enforcement or knockdown when data were examined by treatment group (Figure 10). Western blots for proteasome subunits PA28γ, α3, and β5 did not reveal proteasome enforcement by AAV-PA28γ-GFP or knockdown by AAV-shRNA to PA28γ-GFP (Figure 11 and Supplemental Figure S3).

### 3.4. Alternate Mechanisms for OLE

**Figure 12:**
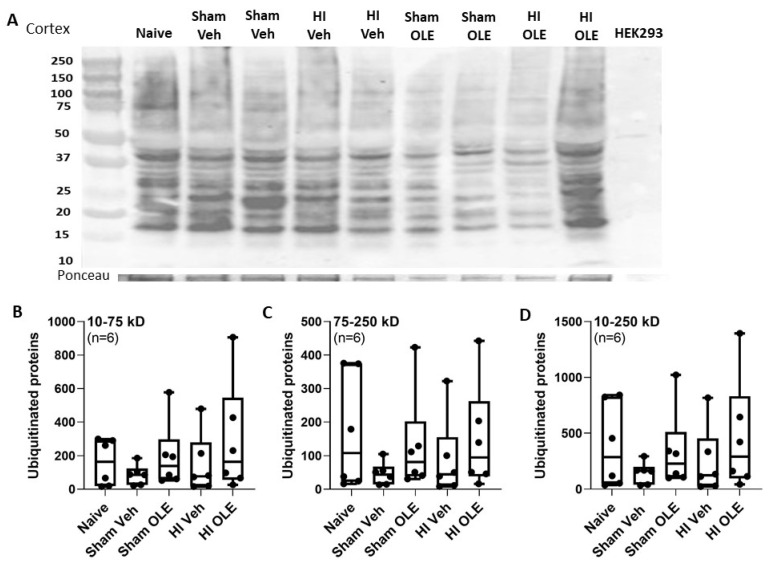
Western blots of ubiquitinated protein immunoreactivity in the somatosensory cortex of piglets that received hypoxia-ischemia (HI) or sham procedure with oleuropein (OLE) or vehicle (veh). (**A**) Full length western blots. (**B**–**D**) When proteins were analyzed according to molecular weight, the ubiquitinated protein levels did not differ in the 10–75 kD (*p* = 0.775), 75–250 kD (*p* = 0.485), or 10–250 kD (*p* = 0.653) ranges. The box plot whiskers show the 5th and 95th percentiles.

**Figure 13:**
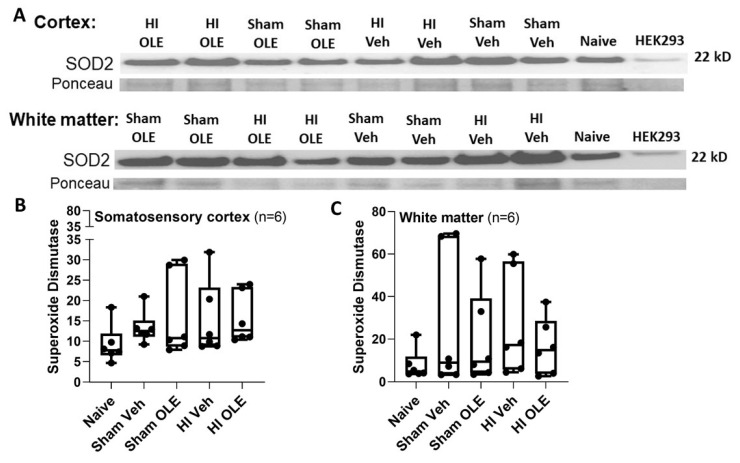
Western blots for manganese superoxide dismutase (SOD2) immunoreactivity in somatosensory cortex and white matter of piglets that received hypoxia-ischemia (HI) or sham procedure with oleuropein (OLE) or vehicle (veh, **A**). SOD2 levels did not differ in the cortex (*p* = 0.125, **B**) or in white matter (*p* = 0.609, **C**). The box plot whiskers show the 5th and 95th percentiles.

**Figure 14:**
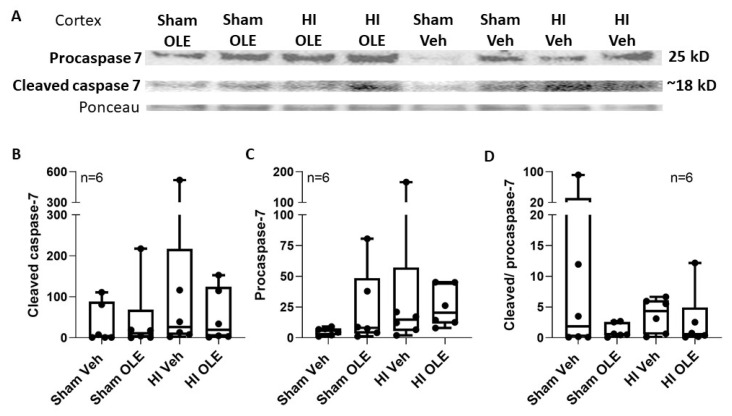
Caspase-7 immunoreactivity in somatosensory cortex 29 h after hypoxia-ischemia (HI) or sham procedure with hypothermia. Piglets also received oleuropein (OLE) or vehicle (veh). (**A**) Western blots did not show differences between groups for cleaved caspase-7 (*p* = 0.466, **B**), procaspase-7 (*p* = 0.082, **C**), or the cleaved-to-procaspase-7 ratio (*p* = 0.0631, **D**). The box plot whiskers show the 5th and 95th percentiles.

We evaluated whether OLE affected the oxidative stress response upstream from the proteasome or cell death. Ubiquitinated protein levels in the cortex did not differ among proteins with the molecular weights 10–75 kD (*p* = 0.775), 75–250 kD (*p* = 0.485), or 10–250 kD (*p* = 0.653; Figure 12). SOD2, a marker of the oxidative stress response, was detected in piglet brain at a molecular weight consistent with other species, including human [57,58]. OLE after HI did not affect SOD2 levels in somatosensory cortex (*p* = 0.125) or subcortical white matter (*p* = 0.609; Figure 13). The cell death related procaspase-7 and cleaved caspase-7 proteins were detected in piglet brain at the expected molecular weights [53] without a significant effect from OLE (*p* = 0.0466 for cleaved caspase-7, *p* = 0.082 for procaspase-7, and *p* = 0.0631 for cleaved/procaspase-7; Figure 14).

### 3.5. LC-MS/MS Identifies Pericentriolar Material 1 Protein and Triosephosphate Isomerase as Major Targets of Oxidative Stress in Neonatal Piglet White Matter after HI and Hypothermia

About 758 different proteins were identified by LC-MS/MS in piglet subcortical white matter. The top 25 proteins in the size range of 20–30 kDa are shown in Supplemental Table S1. Three of these proteins were oligodendrocyte-specific myelin-related proteins: myelin basic protein, myelin proteolipid protein, and myelin-oligodendrocyte glycoprotein. Other proteins enriched in piglet subcortical white matter belonged to the molecular chaperone gene ontology, including Parkinson’s disease protein DJ-1, α-crystalline, and protein 14-3-3. A newer, more recently discovered protein found enriched in piglet white matter was the calcium-sensing protein visinin-like protein 1 [59].

Two proteins were found to be consistently carbonylated in neonatal piglet white matter after HI and hypothermia (Supplemental Figure S4). They matched exclusively to pericentriolar material 1 protein (PCM1, Supplemental Figure S4A–C) and triosephosphate isoform X1 (TPI, Supplemental Figure S4D–F), with a 97–100% probability of correct identity. The total number of peptide fragments detected in the HI-normothermia piglets (8 peptides) and in the HI-hypothermia piglets (20 peptides), plus additional peptides (17 peptides) from piglets not included in this study because they received different treatments (data not shown), was sufficient to yield this high probability of specific identification (Supplemental Figure S4C,F). PCM1 (XP_005672708.1) was carbonylated at arginine-137 (Supplemental Figure S4A,B). TPI (XP_005652645.1) was carbonylated at proline-287 and threonine-293 (Supplemental Figure S4D,E,G). Lysine carbonylation was not seen in either protein in any peptides or in any novel 20–30 kDa protein from any piglet. One piglet in the HI-normothermia group had carbonylation in centrobin (XP_020921976.1) at arginine-95 (data not shown). One piglet in the HI-hypothermia group had carbonylation in phosphatidylinositol-glycan-specific phospholipase D (XP_001925944.5) at threonine-679 and arginine-680 (data not shown). Descriptively, there were no apparent differences in the relative levels of PCM1 arginine-137 carbonylation among the two piglet treatments (Supplemental Figure S5).

## 4. Discussion

We undertook this study to learn about proteasome activity, subunit distribution, and HI vulnerability and to test potential proteasome modulators in neonatal piglets. This interest stems from work showing that our piglet model mimics the clinical physiology, neuropathology, neuronal death, therapeutic responses, and long-term cognitive outcomes seen in human infants who receive therapeutic hypothermia for HIE [32,34,35,37,43,60,61,62,63,64]. Some infants who receive hypothermia for HIE still have persistent white matter injury on MRI [32,33,60,63,64,65,66,67,68]. We found in piglets that white matter proteinopathy and oligodendrocyte pathology are major phenotypes of hypothermia-resistant brain injury after HI [6]. Insufficient degradation of damaged proteins, including oxidized, nitrated, cleaved, misfolded, dityrosine cross-linked, and aggregated proteins alongside a loss of proteasome function, could contribute to HI brain injury even with therapeutic hypothermia. OLE can partly protect against HI-induced molecular and cellular pathology in piglet white matter [6,23]. Other than a few molecular cloning studies of swine proteasome subunit genes [69,70,71,72], there is scant information available on swine proteasomes. Little work has been done to characterize newborn piglet proteasomes with the exception of P20S immunolocalization in brain [6]. Therefore, we designed this study to examine proteasome activity in different neonatal piglet tissues, compare proteasome activity in newborn and juvenile brain, and determine the effects of overnight anesthesia and whole-body hypothermia on brain proteasome activity with and without an underlying HI injury because these latter goals are directly clinically relevant. We also tested OLE as a potential brain proteasome activator and sought to develop a virus-mediated strategy to transgenically manipulate brain proteasomes in neonatal piglets for therapeutic purposes.

### 4.1. Tissue Distribution of Proteasome Activity Varies in Neonatal Piglets

Proteasomes are multicatalytic proteinase complexes with three activity classifications: chymotrypsin-like, trypsin-like, and caspase-like peptidyl-glutamyl-peptide hydrolyzing [73]. We assayed for chymotrypsin-like proteasome activity in the piglet cerebral cortex and white matter, liver, and kidney. The β5 subunit possesses the chymotrypsin-like activity; it cleaves the fluorogenic substrate SUC-LLVY-AMC and other peptide bonds at the C-terminal side of hydrophobic amino acid residues [48]. We focused on the proteasome’s chymotrypsin-like peptidase activity given its association with brain injury, including its decline after traumatic brain injury in adult rats [74].

Chymotrypsin-like proteasome activity was higher in piglet brain than in liver and kidney. Rat proteasome subunit concentration and proteolytic function also vary by organ [73,75]. Piglets and rats appear to have more proteasome chymotrypsin-like activity in brain than in kidney [76]. However, in contrast to our piglet data, adult rat proteasome activity is higher in the liver than in the brain [76,77]. We have, thus, identified a species difference in proteasome tissue distribution. It has been theorized that the higher proteasome activity in rat liver might enable better damage control for oxidative stress, whereas the rat brain’s lower proteasome activity could render it more vulnerable to oxidative stress [77]. Our data suggest that this theory from rat data probably does not apply to all species. Longer-lived gyrencephalic mammals, such as pigs, seemingly have a higher proteasome capacity in the brain than in peripheral organs, but we have not studied proteasomes in human tissue. A greater proteasome capacity could be an evolutionary trait for managing aging or may be related to environmental factors, including diet. Species differences must be considered when designing and interpreting brain proteasome studies.

The proteasome’s native state and vulnerability at different maturational stages must be clarified before neuroprotective strategies can be developed to reduce proteinopathy and neural dysfunction throughout the lifespan. We found that neonatal piglets had greater proteasome activity in the subcortical white matter than did juvenile pigs. It appears that neonatal white matter in its uninjured state has a high proteasome activity level that declines with development. The high neonatal baseline proteasome requirement suggests that proteasome impairments early in life could put subsequent white matter development at risk.

We also measured proteasome subunits by western blot in naïve, unanesthetized piglets. The proteasome α3 subunit is part of the α1–7 structural rings that flank the double β ring core with the proteolytically active β subunits. The β5 subunit level in subcortical white matter exceeded that of somatosensory cortex, whereas α3 subunit levels did not differ between gray and white matter. The finding that the β5 level, but not activity, was higher in uninjured white matter suggests that the subcortical white matter has a baseline reserve of β5 subunits that is not engaged in proteinase activity.

### 4.2. Brain Proteasome Activity with Hypothermia, HI, and Anesthesia

In our past work, we identified an association between ubiquitinated and carbonylated protein accumulation and myelin injury in piglets with HI and hypothermia. This pathology was related to a reduced number of P20S immunoreactive glia in the subcortical white matter and internal capsule. Moreover, subcortical white matter P20S levels declined between 20 and 29 h of hypothermia after HI [6]. OLE prevented the loss of myelinating oligodendrocytes and myelin after HI and hypothermia [23]. This information led us to further examine proteasome activity after HI and hypothermia and to test whether OLE modulated proteasomes as a potential mechanism for its white matter protection.

Because the proteasome assay is a kinetic assay, we varied the brain homogenate input while keeping the substrate concentration constant. This allowed us to examine the assay’s sensitivity, identify relationships between the proteasome load into the assay and proteasome activity, and test whether piglet treatment affects this relationship. The western blots showed that β5 subunit levels did not differ among treatment groups. Thus, β5 content at similar protein loads can be assumed to be equivalent among the treatments. We found that the starting t_0_ proteasome activity increased with greater protein input from cerebral cortex and white matter of naïve piglets. The OLE- and vehicle-treated sham groups also had higher white matter t_0_ proteasome activity levels with more protein. However, in sham hypothermic piglets that received vehicle, there was no relationship between assay protein input and cortical t_0_ activity. OLE restored the expected increase in cortical t_0_ proteasome activity with sham hypothermia. Interestingly, HI + hypothermia piglets with and without OLE failed to show augmented t_0_ activity in either cortex or white matter with greater protein input.

Proteasomes undergo multiple complex steps to degrade proteins. The t_0_ proteasome activity may represent the proteasome’s initial substrate binding, docking, and conformational changes that open the gate and pass the substrate through the catalytic core [78]. The P20S proteasome gate opening is controlled by tight allosteric coupling to a tetrahedral transition state regulated by α-ring subunit N-terminal regions that trigger the conformational switch [79]. Our data suggest that these initial processes are slowed by hypothermia in neonatal cortex and restored by OLE.

HI may reduce the proteasome’s substrate binding affinity or slow the conformational changes [79] that are required to rapidly initiate proteolysis. Alternatively, existing damaged proteins in the HI brain tissue homogenates could compete for AMC substrate binding and slow the initial fluorescence signal. Nonetheless, cortical and white matter proteasome activity increased during the entire assay reaction with higher protein input in HI piglets that received OLE or vehicle. Naïve piglets showed the same increase in proteasome activity over reaction time. The absent change in proteasome activity in sham piglets suggests that hypothermia per se alters proteasome function.

One possible explanation for our data is that neonatal HI and hypothermia induce proteasome changes, whereby the P20S core and proteasome activating subunits form large proteasome structures that ultimately accelerate catalytic activity. Proteasomes exist in multiple configurations [80]. These include the free P20S catalytic core, P26S proteasome assembly with the P20S core and 19S regulatory particle, P20S core with proteasome activator subunits, and other hybrids. Several diseases alter proteasome structure and activity [78], including cardiac ischemia [81].

Neonatal HI may cause a dynamic interplay among individual proteasomes to form polyribosome-like complexes. The large proteasome structure’s greater complexity could slow substrate binding, potentially by reducing substrate access to the docking site, and cause a lower t_0_ activity level. Alternatively, HI might induce proteasome complexes that preferentially bind damaged, misfolded, or intrinsically disordered proteins rather than artificial synthetic substrates. Additionally, hypothermia alone may reduce the formation of large proteasome structures that promote rapid proteolysis, similar to how polyribosomes can undergo disaggregation with cerebral ischemia [82]. We found that a high assay protein input (100 µg) was required to identify high proteasome activity after HI and in naïve piglets. Perhaps hybrid proteasome structures assemble only beyond a proteasome subunit concentration threshold, which 100 µg of protein surpassed. Though western blots for the proteasome subunits α3 and β5 in HI and sham piglets showed no differences, we did not measure proteasome activator subunits or search for the formation of proteasome complexes. Future studies are needed to examine how neonatal HI and hypothermia affect the formation of large proteasome structures, substrate binding, and conformational change relative to catalytic rate.

We additionally explored peri-insult blood gas and hemodynamic biomarkers that might relate to subsequent proteasome activity to look for a clinically accessible physiologic metric that might prognosticate brain proteasome activity. Lower arterial pH 1 h after resuscitation correlated with greater proteasome activity in the cortex, but not white matter, at 29 h of hypothermia and rewarming. This finding links the systemic physiologic perturbation to a cerebral response. Acidosis causes amino acid oxidation and triggers the ATP-dependent, proteasome-ubiquitin pathway [83]. Changes in pH as small as 0.2 increase proteolysis in the liver [84]. The piglets’ 1-h pH ranged from 7.06 to 7.42. We previously showed that piglets undergoing the same HI and hypothermia protocol have significant oligodendrocyte and myelin damage with little cortical neuron cell body injury after HI [23]. It is unclear if the high cortical proteasome activity was protective or a response to cortical protein damage. The white matter proteasome response to piglet HI also lags behind that of cortex [6], and we only studied the subacute period. No relationship between early blood pressure and brain proteasome activity was found.

It is also possible that other proteasome catalytic activities were affected by HI. These include trypsin-like, peptidyl-glutamyl peptide hydrolase, branched chain amino acid preferring, and small neutral amino acid preferring proteolysis [76]. We will measure these proteasome functions in future studies.

### 4.3. OLE Is a Neocortical Gray Matter Proteasome Activator

OLE has shown potential for the treatment of cancer, cardiovascular disease, and Alzheimer’s disease [85,86,87,88]. We also found that OLE protects myelinating oligodendrocytes and myelin after piglet HI and hypothermia [23]. Reports indicate that OLE can modulate oxidative stress [25,26], autophagy [24], and inflammation [25]. These features make OLE an appealing therapeutic candidate for HIE because it targets multiple pathways [89]. It is also suggested that OLE modulates the proteasome [16].

We confirmed that OLE activated cortical proteasomes at t_0_ during hypothermia in piglets that underwent the sham procedure. Because cortical and white matter proteasome activity increased over the entire assay reaction in HI piglets that received OLE and vehicle, HI itself activates neonatal brain proteasomes. Moreover, OLE did not rescue the t_0_ proteasome activity after HI. Thus, white matter protection from hypothermia and OLE [23] is not related to the proteasome’s chymotrypsin-like activity.

OLE did not change the antioxidant enzyme SOD2 [54] protein levels in the cortex or white matter, though we did not assess its enzymatic activity. OLE also did not affect cortical ubiquitinated protein levels after HI by western blot. In piglet white matter, ubiquitinated protein levels significantly increase whereas cortical ubiquitinated proteins decline between 20 and 29 h of hypothermic recovery after HI [6]. We examined only one time point in the current study. The modest changes in cortical proteasome activity did not affect global protein ubiquitination. We did not study the ubiquitination of specific protein targets or whether ubiquitination changed across time.

The 29-h recovery may have been too early for cell death to evolve with sufficient cleaved caspase-7 that could be detected by western blot in crude homogenate. However, we designed our study to interrogate the proteasome independent of major neurodegeneration, which could confound proteasome measures. Alternatively, hypothermia may have protected the cortex. Future studies on OLE’s effects at different recovery time points, normothermia, and the possible roles of autophagy, inflammation, and other proteasome peptidase functions are needed.

### 4.4. Conditional Transgenic Modification of the Neonatal Piglet Brain with AAV

To complement our pharmacologic approach to activate the proteasome, we developed a protocol for stereotaxic AAV intracerebral injections and tested the feasibility of modulating proteasome gene expression and activity in neonatal piglet brain. Transgenic pigs are currently an impractical experimental model for HIE. We used AAV serotype 9, which is under clinical therapeutic investigation for spinal muscular atrophy [29]. We focused on the PA28γ subunit (PSME3) to avoid potential cell lethality confounders and because PA28γ activates P20S in brain cells [52]. Moreover, PA28γ augments the 20S proteasome’s function in degrading oxidized proteins [90], which we found to accumulate in neonatal piglet white matter after HI, as shown here with protein resolution and globally before [6]. Our paradigm used whole-body hypothermia because this is the standard of clinical care for HIE [32,33].

GFP western blotting verified successful AAV transduction in the targeted somatosensory cortex and subcortical white matter of cooled sham piglets. We also showed that AAV-mediated GFP transduction can be achieved after HI and hypothermia. Thus, AAV is a tool that can be used to genetically modify piglet gray and white matter after HI and during hypothermia in future studies.

Though PA28γ modulation was variable, higher subcortical white matter PA28γ levels correlated with greater proteasome activity across assay reaction time, higher starting t_0_ activity, and higher P20S β5 subunit levels. The cortex also showed a direct correlation between PA28γ and β5 levels, but without a difference in proteasome activity. Interestingly, lower cortical P20S α3 subunit levels were associated with higher PA28γ. Proteasome-PA28γ complexes are highly enriched in the nucleus, where they are known to function in nuclear substructure, nuclear protein turnover, and mRNA splicing [91]. The transcriptional regulation of individual proteasome subunit genes is under tight control with positive and negative influence of their promoters by zinc-finger proteins Zif268 (Egr-1) and CCAAT box-binding nuclear factor Y [92]. The α3 subunit gene promoter is activated by nuclear factor Y [93]. It is, therefore, possible that higher PA28γ downregulates nuclear transcription factors that activate the α3 subunit gene promoter in a tissue-specific and context-dependent manner. The dynamic interplay of the proteasome within the nucleus and transcription factor biology of swine requires further study.

The significant relationships between PA28γ and proteasome activity, β5, and α3 occurred only in the cerebral hemisphere that received the F108 and polybrene adjuvants in cocktails with higher AAV doses. However, when data were analyzed by protocol, the AAV shRNA knockdown or enforcement vectors for PA28γ neither consistently altered proteasome chymotrypsin-like peptidase activity nor modulated PA28γ, α3, and β5 subunit levels. PA28γ shRNA paradoxically increased proteasome activity in some piglets. Mammalian cell culture experiments, including those in human and non-human primate cell lines, show that proteasome inhibition can actually upregulate transcriptional proteasome subunit expression and promote the *de novo* formation of new proteasomes [94]. This autoregulatory feedback mechanism sustains the proteasome in the face of proteasome inhibition, and it may have prevented our efforts to knock down the proteasome using AAV. The low gene targeting efficacy may also have been related to shRNA targeting based on human sequences and use of the human open reading frame from cDNA for overexpression, as it has several intragenic CpGs that could mediate silencing through host DNA methylation mechanisms. The apparent GFP transduction in the cortex and white matter ruled out an immunologic response to AAV that could have prevented proteasome modulation. Thus, the proteasome in piglet brain is difficult to genetically modify using AAV.

### 4.5. Proteomic Profiling of White Matter in HI Piglets

We used a protein carbonyl detection system in combination with LC-MS/MS to identify oxidative damage to the proteome of subcortical white matter of neonatal HI piglets. In this pilot study, we limited the analysis to proteins within the 20–30 kDa range. The global proteome profile within this size range showed relative enrichment of three well-known white matter proteins, including myelin basic protein, myelin proteolipid protein, and myelin-oligodendrocyte glycoprotein (Supplemental Table S1). This finding is a validation of our microdissection approach [23]. We found with high probability that PCM1 and TPI in HI piglet white matter are oxidatively damaged by carbonyl modification. We are uncertain if this post-translation modification alters the function of these proteins. PCM1 could be important in our neonatal brain injury model because, in addition to its roles in cytoskeletal biology, it functions in DNA damage response [95] and in glial cell death [96]. Another protein that was identified potentially as oxidatively damaged only in some HI piglets was centrobin. Centrobin also functions in DNA damage response [97]. Oxidative proteinopathy in TPI is relevant to our brain injury model because of its function in glucose metabolism [98], which is critical for metabolically active oligodendrocytes [99]. Moreover, loss of function of TPI from autosomal recessive genetic mutations cause multisystem disease with neurological phenotypes, leading to death in early childhood [98].

### 4.6. Strengths and Limitations

Because the proteasome properties of the human brain are more similar to those of the piglet than to those of the rodent, piglets have high potential as a clinically relevant model for proteasome research [12,14,15]. The piglet model also provides an excellent opportunity to study oxidative proteinopathy by proteomics and differences in regional gray and white matter responses to HI and hypothermia with continuous hemodynamic and core temperature monitoring. Humans and piglets have analogous perinatal brain development, including prenatal and extended postnatal myelination and similar brain development levels at term gestation. They also have analogous connectivity and are both gyrencephalic [23,100,101,102,103,104,105,106,107,108,109]. Importantly, piglet HI causes regional cerebral injury that mimics the neuropathology of full-term newborns with HIE [17]. We used whole body cooling for a duration that protects piglet neurons [23,36] and the clinical rewarming rate of 0.5 °C/h [32,33,60,63].

We focused only on the chymotrypsin-like activity of proteasomes, but HI or OLE could have affected other catalytic activities. We did not examine how longer durations of hypothermia affect brain proteasomes, even though 72 h of hypothermia is standard treatment for HIE [32,33,60]. We also did not test whether proteasome activity declines during normothermic recovery from HI, and we did not measure the bioavailability of OLE in the brain. We conducted the AAV experiments in a limited number of piglets to determine feasibility of transgenic manipulation in the relevant setting of hypothermia. Though we successfully induced AAV transduction of GFP in cooled piglet brain, more work is needed to explore whether genetic strategies for proteasome knockdown or enforcement in piglet brain can be achieved using recombinant virus-encoding swine gene sequences. The OLE and AAV studies were conducted in hypothermic piglets because clinical adjuvant treatments must be delivered with therapeutic hypothermia. Future studies will include normothermic controls. The small number of normothermic HI piglets used for proteomics shows feasibility, but this pilot proteomic work requires elaboration. Only males were studied, but we will compare both sexes in the future.

### 4.7. Conclusions

We identified several key findings that advance knowledge of the proteasome and oxidative proteinopathy in the neonatal, large-animal brain. The greater chymotrypsin-like proteasome activity in the piglet brain relative to that in the liver shows that species differences in proteasome distribution must be considered when designing and interpreting proteasome studies. Proteasome activity in white matter decreases as brain development progresses. The activity in cortical gray matter is more responsive to early systemic acidosis and hypothermia than that in subcortical white matter. In progress reaction curves, HI appears to slow chymotrypsin-like proteolysis initially in white matter and the cortex, but proteasome activity then amplifies. The neonatal piglet brain is amenable to pharmacologic proteasome manipulation, including OLE activation of cortical proteasomes at t_0_ during hypothermia. However, proteasome chymotrypsin-like peptidase activity is not involved in the mechanisms of OLE-mediated white matter protection after HI [23]. HI per se, not OLE or vehicle, appears to activate brain proteasomes. AAV can be harnessed to genetically modify piglet brain, including after HI and during hypothermia, though proteasome modulation by AAV is difficult to achieve and requires refinement. We also discovered that PCM1 and TPI in neonatal piglet white matter are targets of oxidative carbonyl modification after HI. We conclude that the piglet is a relevant model for studying pharmacologic proteasome manipulation, conditional, AAV-mediated genetic modification, and proteomics in the neonatal brain during whole-body hypothermia and rewarming.

## Data Availability

The data presented in this study are available on request from the corresponding author.

## Supplementary Materials

The following are available online at https://www.mdpi.com/article/10.3390/cells10082120/s1, Figure S1: Comparison of proteasome activity levels in freshly harvested tissue and frozen tissue, Figure S2: The effects of adeno-associated virus injections into the right cerebral hemisphere, Figure S3: Western blot data of proteasome 20S subunits in piglets that received intracerebral adeno-associated virus, Figure S4: LC-MS/MS identifies pericentriolar material 1 and triosephosphate isomerase as targets of oxidative stress in neonatal piglet white matter after hypoxia-ischemia, Figure S5: Relative levels of PCM1 arginine-137 carboxylation in subcortical white matter of hypoxic-ischemic piglets with hypothermia and normothermia, Table S1: Top 25 20–30 kDa proteins enriched in neonatal piglet white matter.
