# Supplementary material for: Oleuropein Activates Neonatal Neocortical Proteasomes, but Proteasome Gene Targeting by AAV9 Is Variable in a Clinically Relevant Piglet Model of Brain Hypoxia-Ischemia and Hypothermia
Source: Cells. 2021 Aug 18;10(8):2120. doi: 10.3390/cells10082120 (PMC8391411; doi:10.3390/cells10082120)

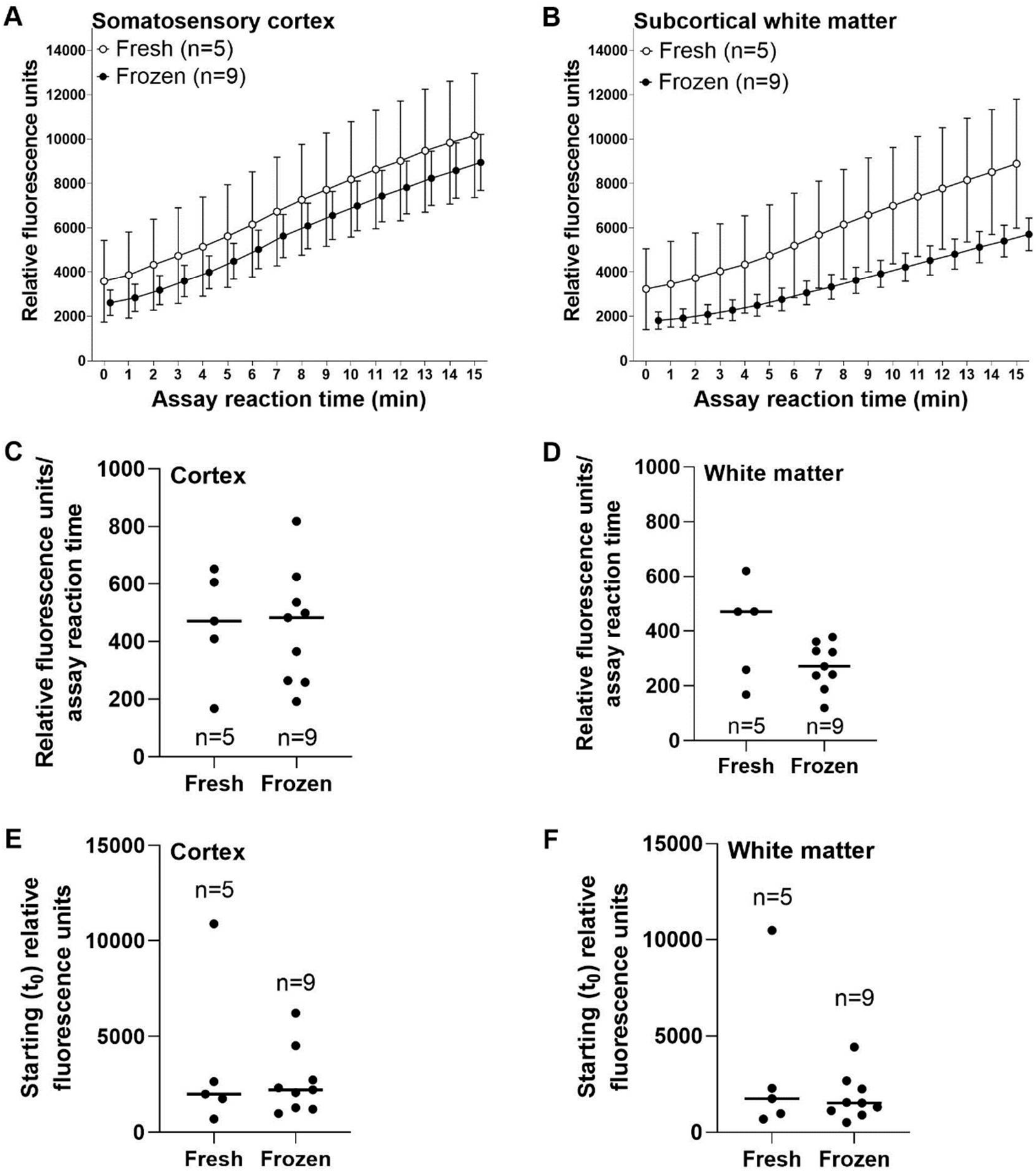

Supplemental Figure S1. Comparison of proteasome activity levels in freshly harvested tissue and tissue frozen for 3.5 years to 2 h at −80 °C from neonatal piglets. (**A**,**B**) Proteasome activity curves across the entire 15-min reaction time. Data are shown as means with standard errors of the mean. (**C**,**D**) The slope of proteasome activity over assay reaction time did not differ in somatosensory cortex ($p > 0.999$) and subcortical white matter ($p = 0.240$). (**E**,**F**) The starting activity level at t_0 also did not differ in cortex ($p = 0.898$) and white matter ($p = 0.797$). For panels (**C–F**), each circle represents one piglet and the line shows the median.

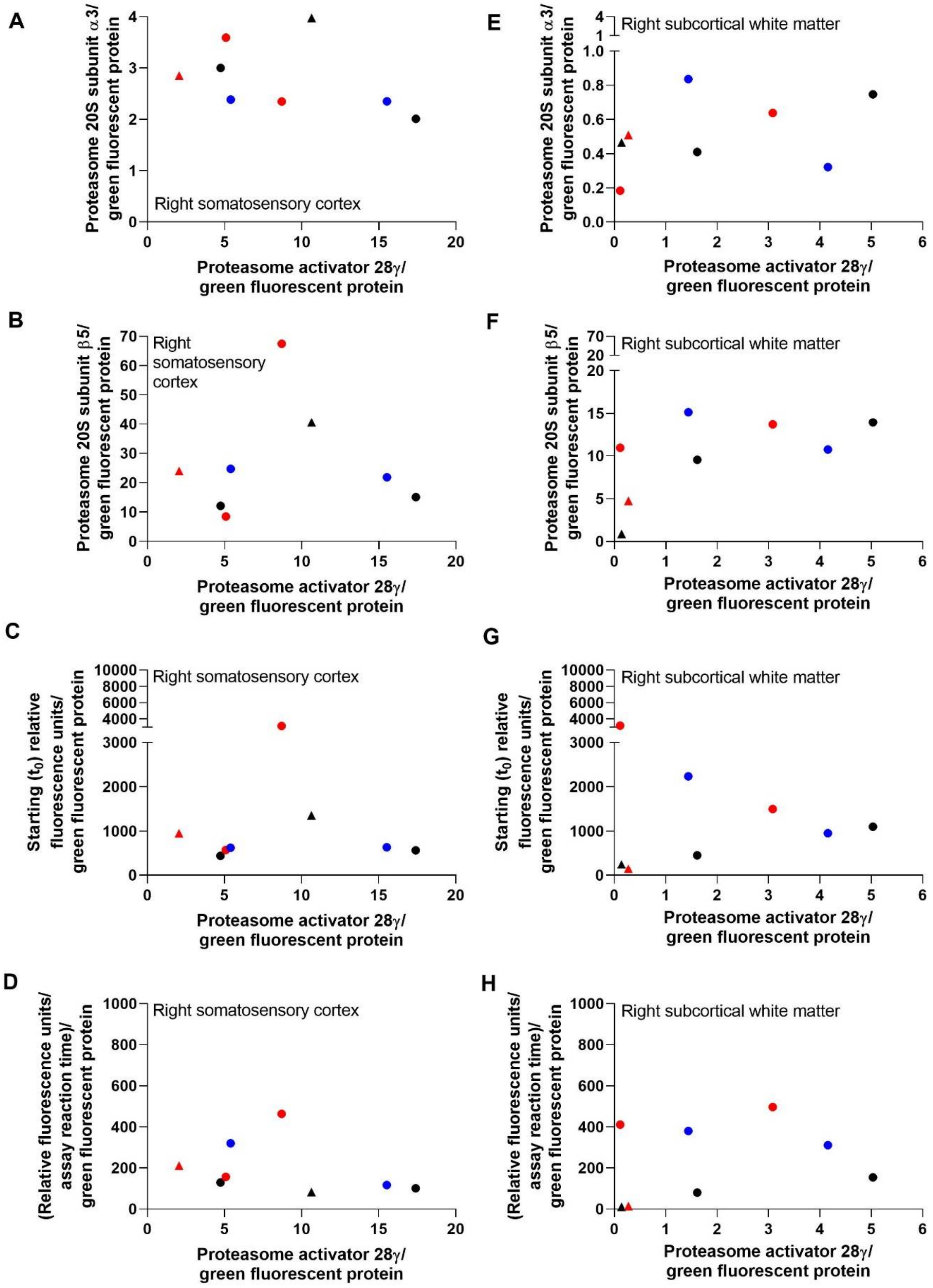

Supplemental Figure S2. In the right cerebral hemisphere, which received intracerebral adeno-associated virus (AAV) injections, proteasome activator 28γ (PA28γ) levels did not correlate with proteasome subunits α3 or β5 or with the slope of proteasome activity over assay reaction time or starting activity at t_0 ($p > 0.05$ for all comparisons). The shapes of the data points indicate the cerebral hemispheric AAV dose: circle = $4 \times 10^{10} – 4 \times 10^{11}$ genome copies (gc), and triangle = $5 \times 10^{10} – 5 \times 10^{11}$ gc. Black: AAV-green fluorescent protein (GFP) control, red: AAV-short hairpin RNA to PA28γ-GFP, and blue: AAV-PA28γ-GFP (enforce). None of these piglets received the F108 or polybrene adjuvant.

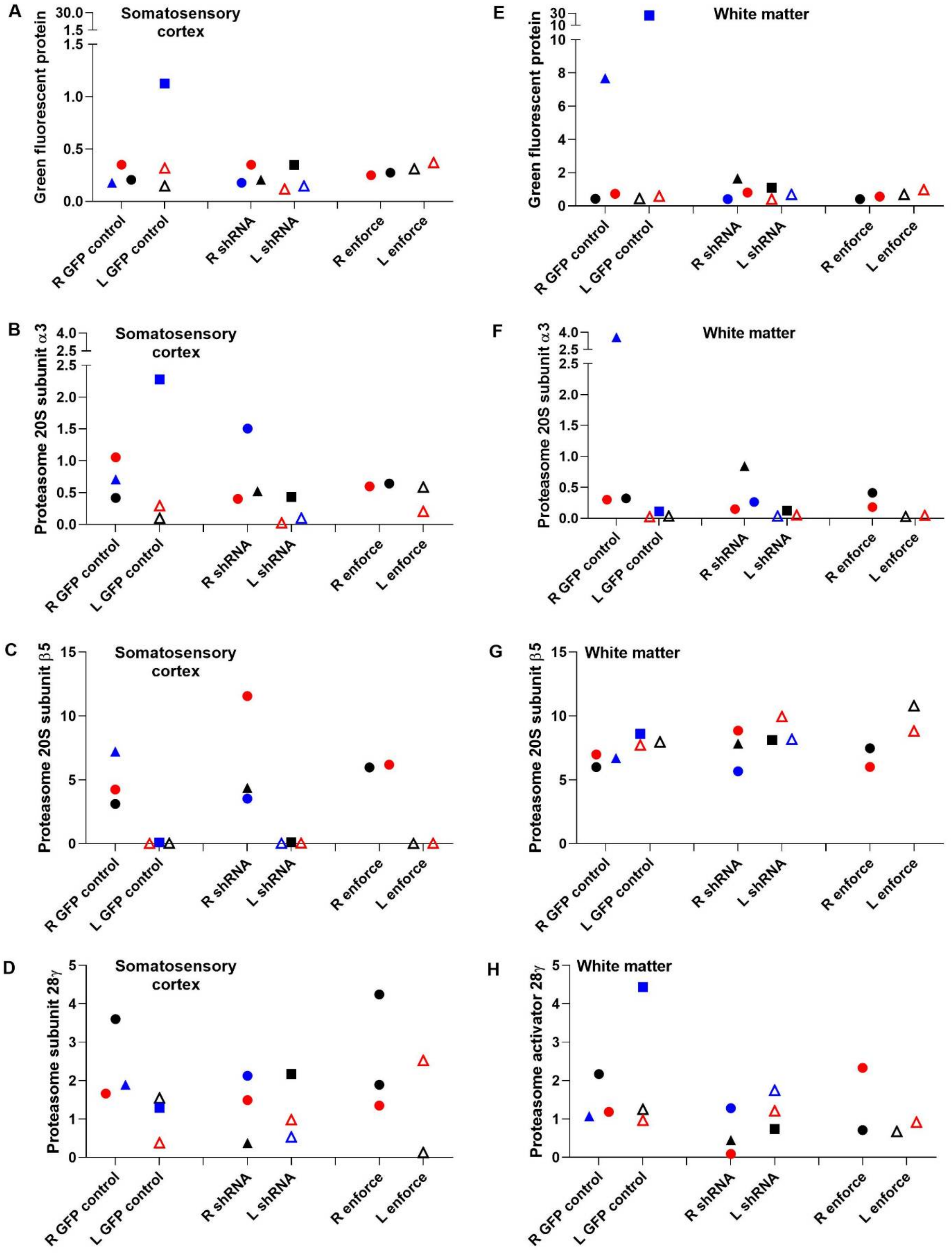

Supplemental Figure S3. Western blots of proteasome 20S subunits in piglets that received intracerebral adeno-associated virus (AAV)-green fluorescent protein (GFP), AAV-short hairpin RNA (shRNA) to proteasome activator 28γ (PA28γ), or AAV-PA28γ-GFP and sham procedure followed by hypothermia. The right (R) and left (L) somatosensory cortex (**A**–**D**) and white matter (**E**–**H**) are shown. (**A**,**E**) GFP levels confirmed successful virus transduction. The F108 and polybrene adjuvant did not increase transduction. (**B**–**D**,**F**–**H**) When compared to AAV-GFP, the AAV-shRNA to PA28γ-GFP did not consistently decrease PA28γ levels or alter the levels of α3 and β5 subunits. AAV-PA28γ-GFP did not increase subunit immunoreactivity. Data points from the same piglet are matched by color within each treatment group. The shapes indicate the cerebral hemispheric AAV dose: square = $2 \times 10^{10} – 2 \times 10^{11}$ genome copies (gc), circle = $4 \times 10^{10} – 4 \times 10^{11}$ gc, and triangle = $5 \times 10^{10} – 5 \times 10^{11}$ gc. Piglets represented by an open symbol also received adjuvant (40 ng of F108 and 2.5 μg of polybrene). Data were divided by a Ponceau-stained protein loading control.

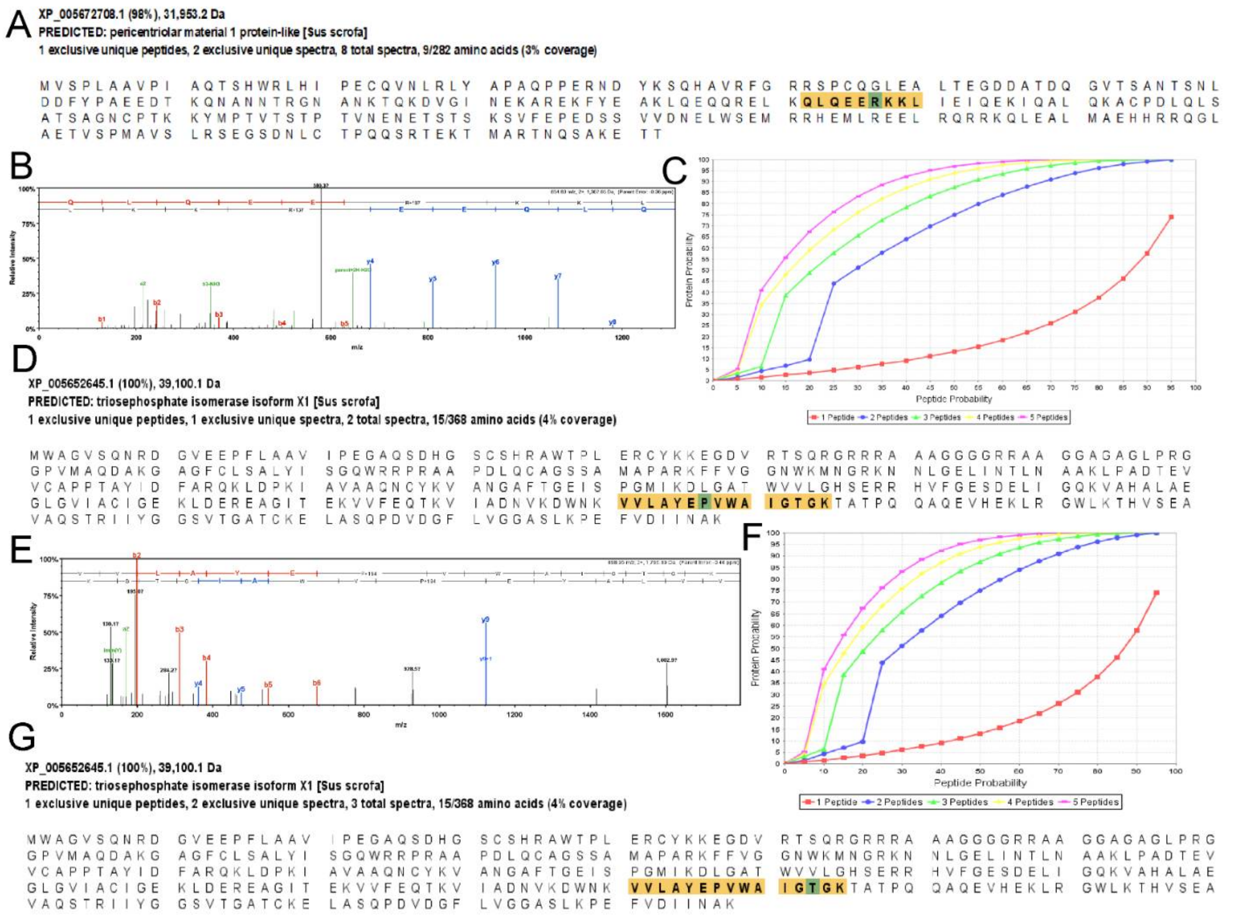

Supplemental Figure S4. Mass spectroscopy identification of pericentriolar material protein 1 (PCM1) and triosephosphate isomerase 1 (TPI) as targets of oxidative damage in neonatal piglet subcortical white matter after hypoxia ischemia and hypothermia. (**A**). Protein sequence of PCM1. Gold shading highlights the tryptic peptide mass spectroscopy fingerprint shown in (**B**). Green shading highlights the arginine-137 DNP-carbonylation within this peptide. (**B**). MS/MS spectrum of a tryptic peptide (9 amino acids in length) derived from oxidized PCM1 used to identify sequence and localize the mono-oxidation site of Arg137. X-axis is mass (m/z). Annotated MS/MS spectrum was exported from Scaffold where b (red)- and y (blue)-type ions represent location of the amino acids shown in the sequences above the MS/MS spectrum. Green identifies amidated and hydrated ionic forms. (**C**). Graph of protein probability determination for PCM1 identity. More than 28 peptides were used to identify PCM1. (**D**). Protein sequence of TPI. Gold shading highlights the tryptic peptide mass spectroscopy fingerprint shown in (**E**). Green shading highlights the proline-287 DNP-carbonylation within this peptide. (**E**). MS/MS spectrum of a tryptic peptide (15 amino acids in length) derived from oxidized TPI used to identify sequence and localize the oxidation site of proline-287. X-axis is mass (m/z). Annotated MS/MS spectrum, exported from Scaffold, identifies b (red)- and y (blue)-type ions registering with location of the amino acids shown in the sequences above the MS/MS spectrum with the prominent y9 (blue) peak corresponding to proline-287 DNP-carbonylation. (**F**). Graph of protein probability determination for TPI identity. The proline-287 oxidation and the identification of this amino acid modification within TPI was determined with 14 different tryptic peptides. (**G**). Protein sequence of TPI. Green shading highlights the threonine-293 DNP-carbonylation within this peptide.

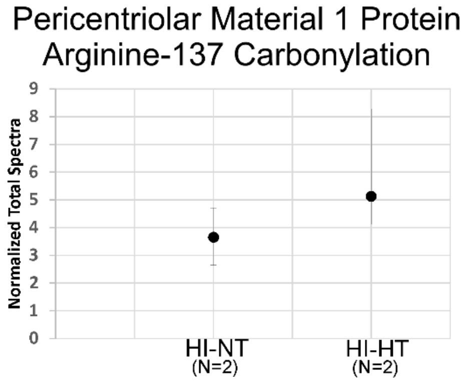

Supplementary Figure S5. Graph of the relative levels of arginine-137 in pericentriolar material 1 protein in a small sample size of hypoxia-ischemia (HI) piglets recovered for 29 hours with normothermia (NT) or hypothermia (HT). Arginine-137 DNP-carbonylation peaks were normalized to total peaks found in tryptic peptides. Values are median and standard deviations.

Supplementary Table S1. Top 25 Unmodified Identified Proteins Enriched in Piglet Subcortical White Matter Found in the 20-30 kDa Size Range.

**Protein**	**Accession Number**	**Molecular Weight (kDa)**
Myelin basic protein	NP_001001546.1	19
Trypsinogen precursor	NP_001156363.1	26
PREDICTED: Triosephosphate isomerase isoform X1	XP_005652645.1	39
PREDICTED: Phosphatidylethanolamine-binding protein 1	XP_003132938.1	21
PREDICTED: 14-3-3 protein zeta/delta isoform X1	XP_001927263.3 (+1)	28
Ubiquitin carboxyl-terminal hydrolase isozyme L1	NP_998928.1	25
Tubulin alpha-1B chain	NP_001038009.1	50
Protein DJ-1	NP_001072131.1	20
PREDICTED: 14-3-3 protein theta isoform 1	XP_003481338.1 (+1)	28
PREDICTED: Phosphoglycerate mutase 1	XP_003483583.1	29
PREDICTED: Visinin-like protein 1 isoform 1	XP_003125391.1	22
PREDICTED: glutathione S-transferase P	XP_005660679.1	22
Myelin proteolipid protein	NP_999139.1	30
Peroxiredoxin-2	NP_001231403.1	22
PREDICTED: Ras-related protein Rab-14 isoform X1	XP_005660460.1	24
Cofilin-1	NP_001004043.1	19
PREDICTED: Alpha-crystallin B chain isoform X1	XP_005667376.1 (+1)	20
PREDICTED: Myelin-oligodendrocyte glycoprotein-like isoformX1	XP_003128291.2	28
RAB11B, member RAS oncogene family	NP_001231804.1	24
ATP synthase, H+ transporting, mitochondrial Fo complex, subunit d	NP_001231613.1	19
PREDICTED: ATP synthase subunit b, mitochondrial	XP_003355292.1	29
PREDICTED: Keratin, type I cytoskeletal 10 isoform X1	XP_003131508.1 (+1)	63
PREDICTED: Ras-related protein Rab-1A isoform X1	XP_005662566.1 (+1)	26
PREDICTED: Synaptosomal-associated protein 25 isoform 2	XP_003483940.1	23

Common Data Element File:
All Western Blots Used for Study

PSMB5
Full-Length Western Blots

Ponceau membrane 1- Somatosensory cortex (SMC)

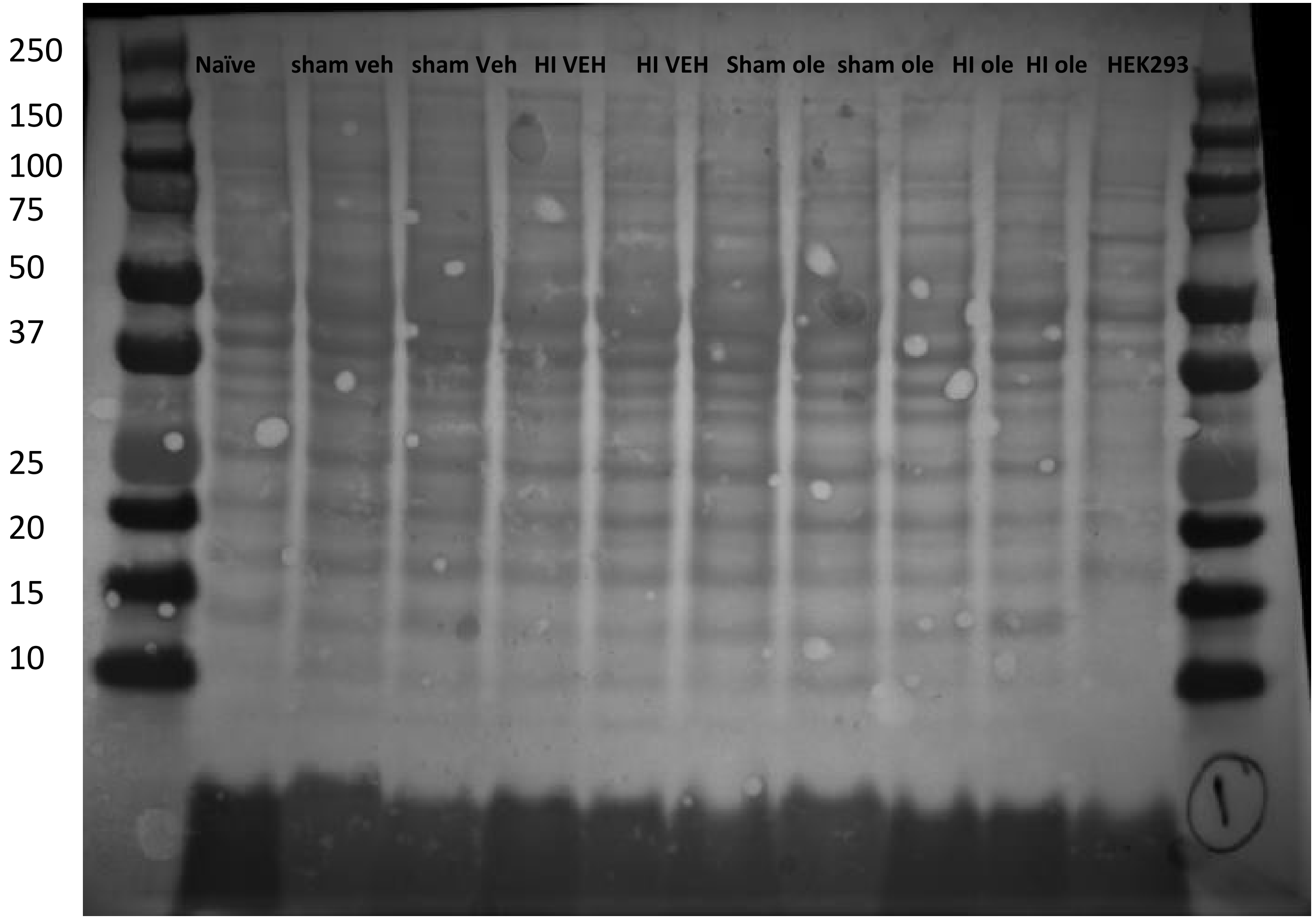

Protein load: SMC 50 µg

Ab . Primary . PSMB5, 1:1000

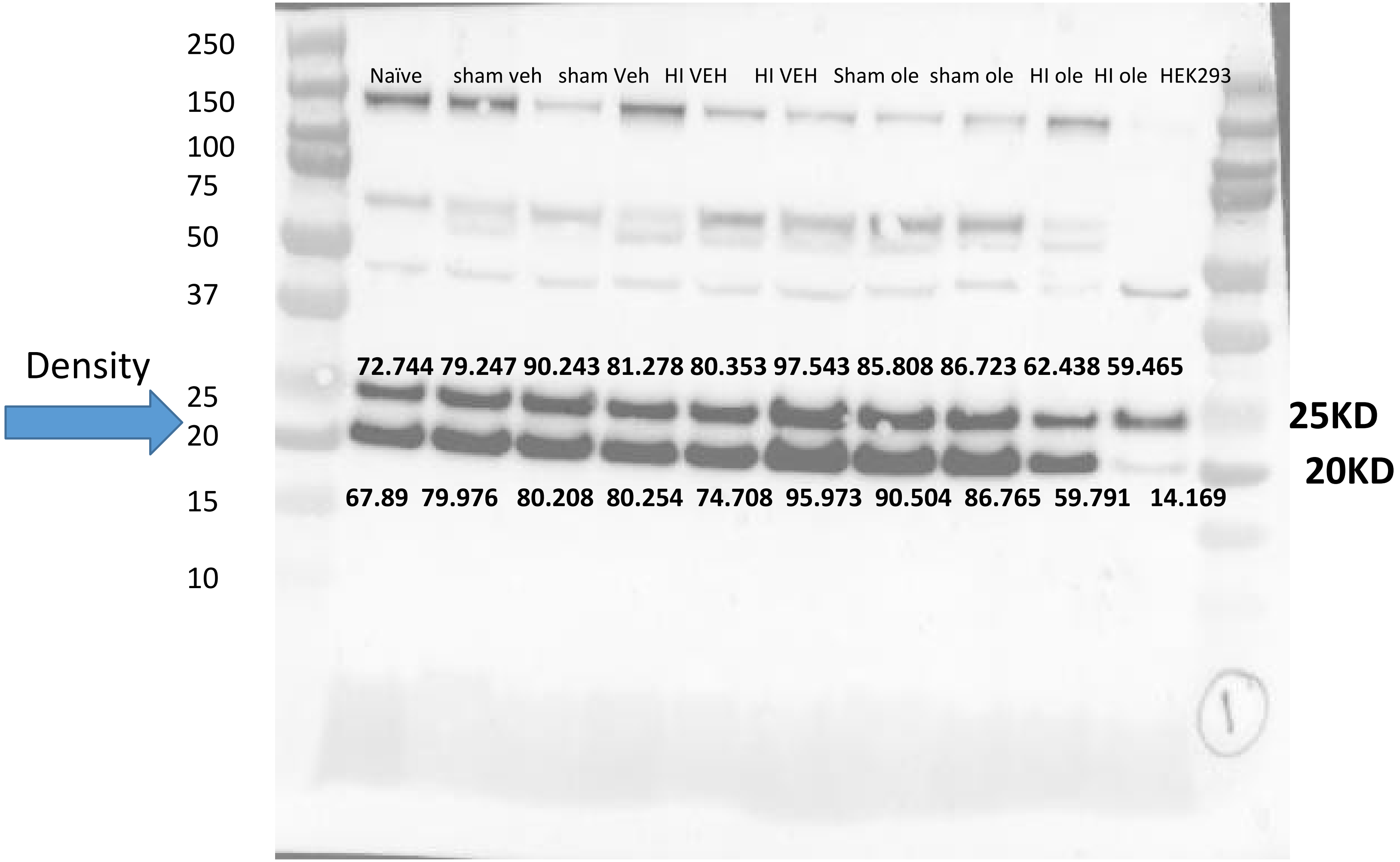

Ponceau membrane 2- white matter (WM)

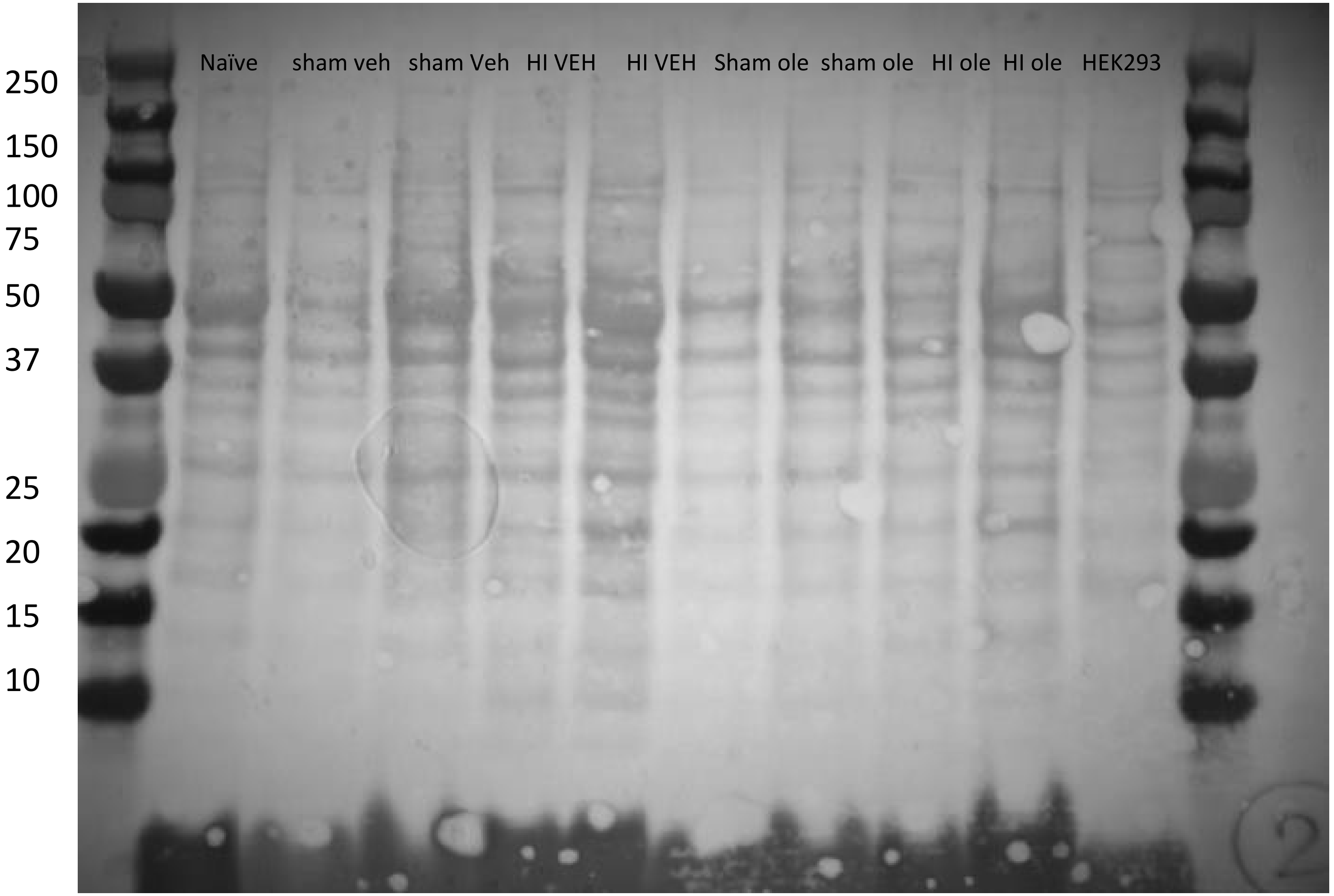

Protein load: WM 50 µg
Ab . Primary . PSMB5 . 1:1000

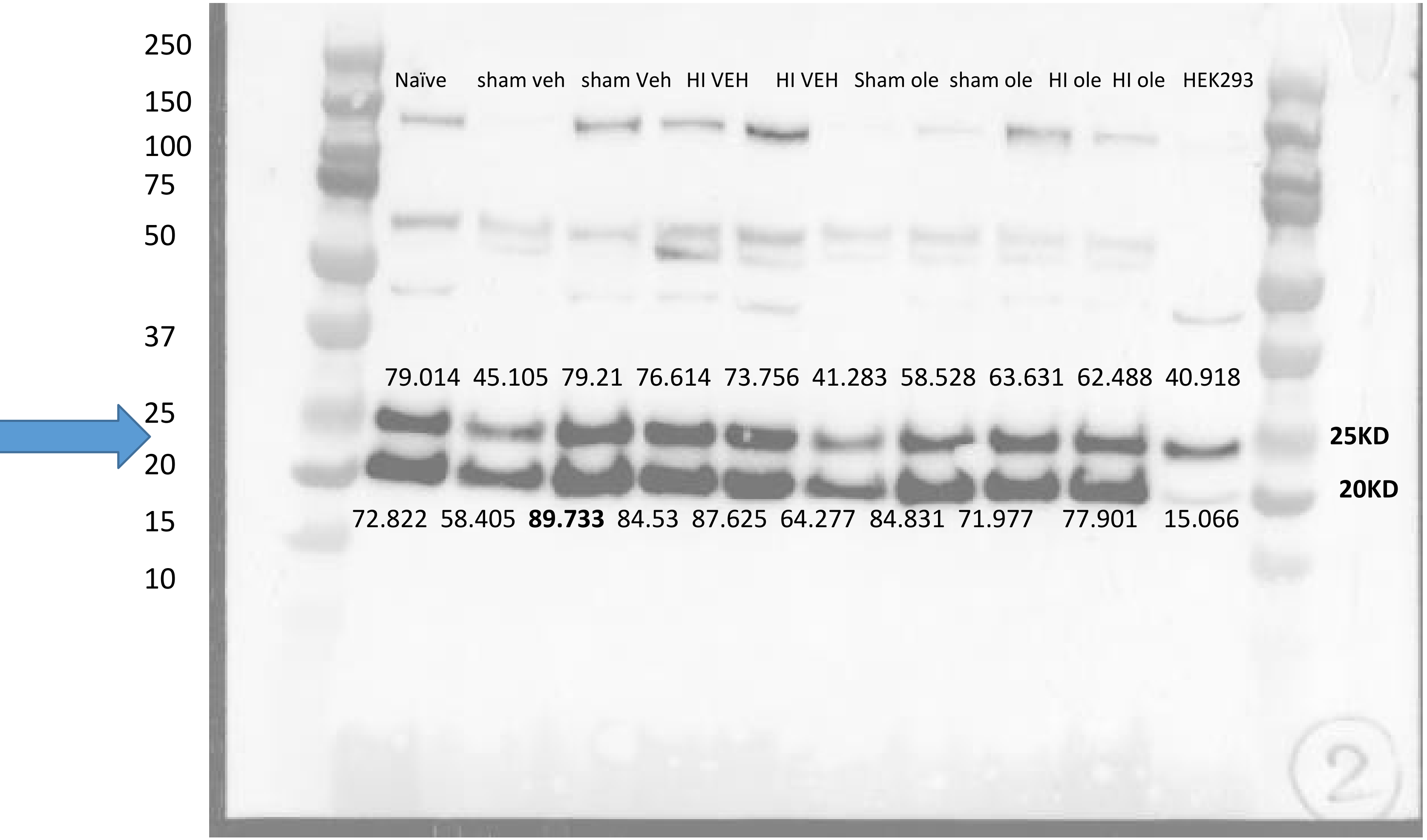

Ponceau membrane 3- SMC

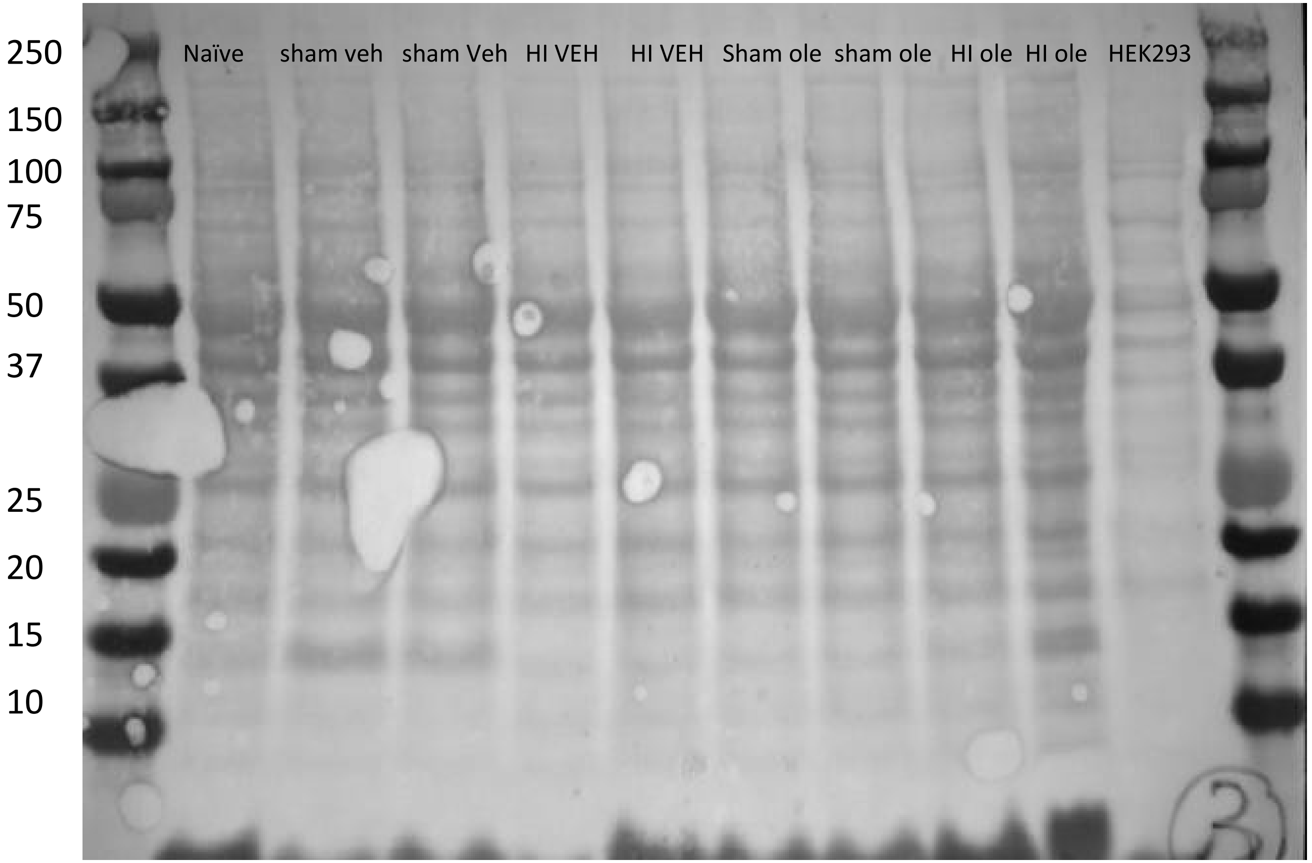

Protein load: SMC 50 µg
Ab . Primary . PSMB5 . 1:1000

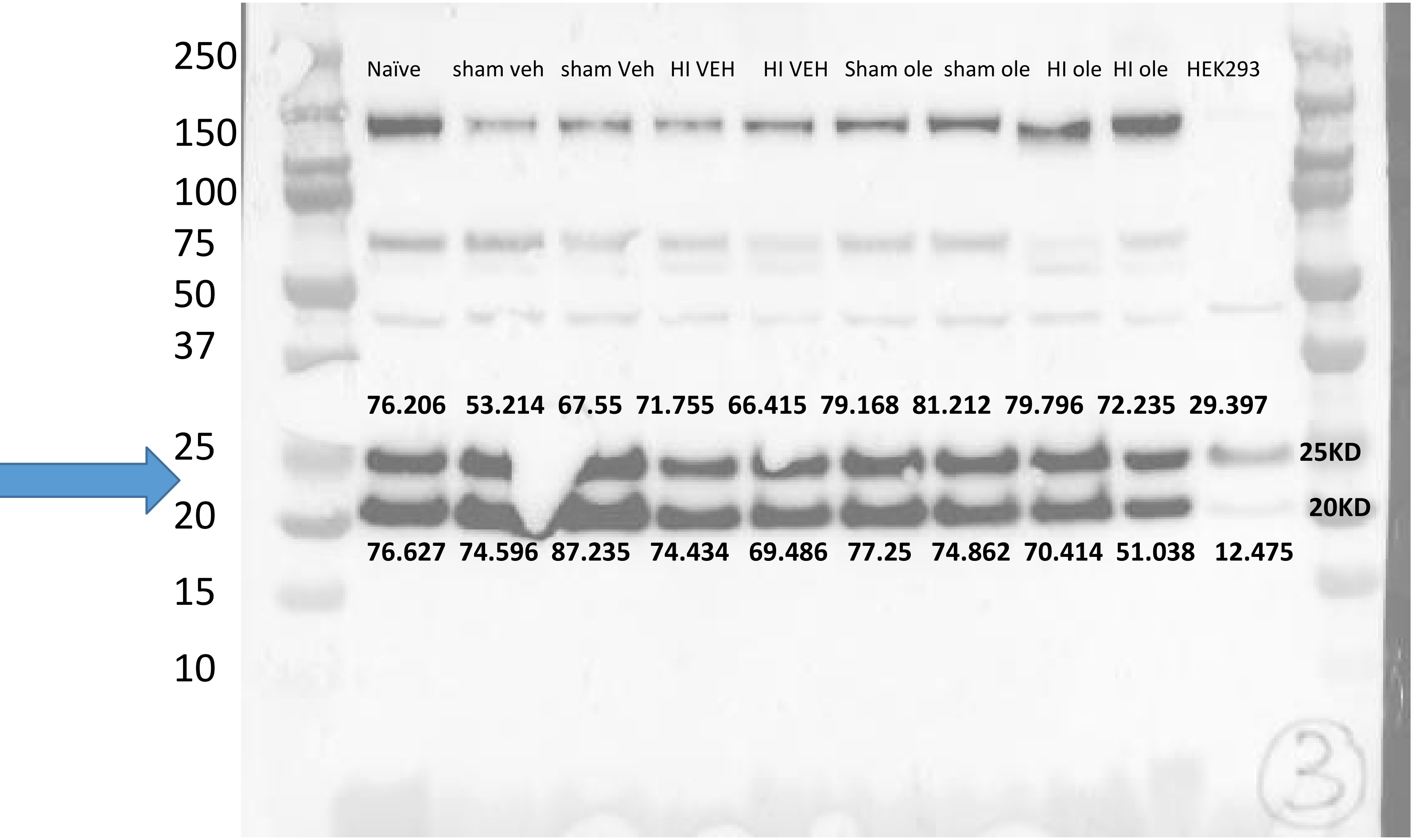

Ponceau membrane 4 - WM

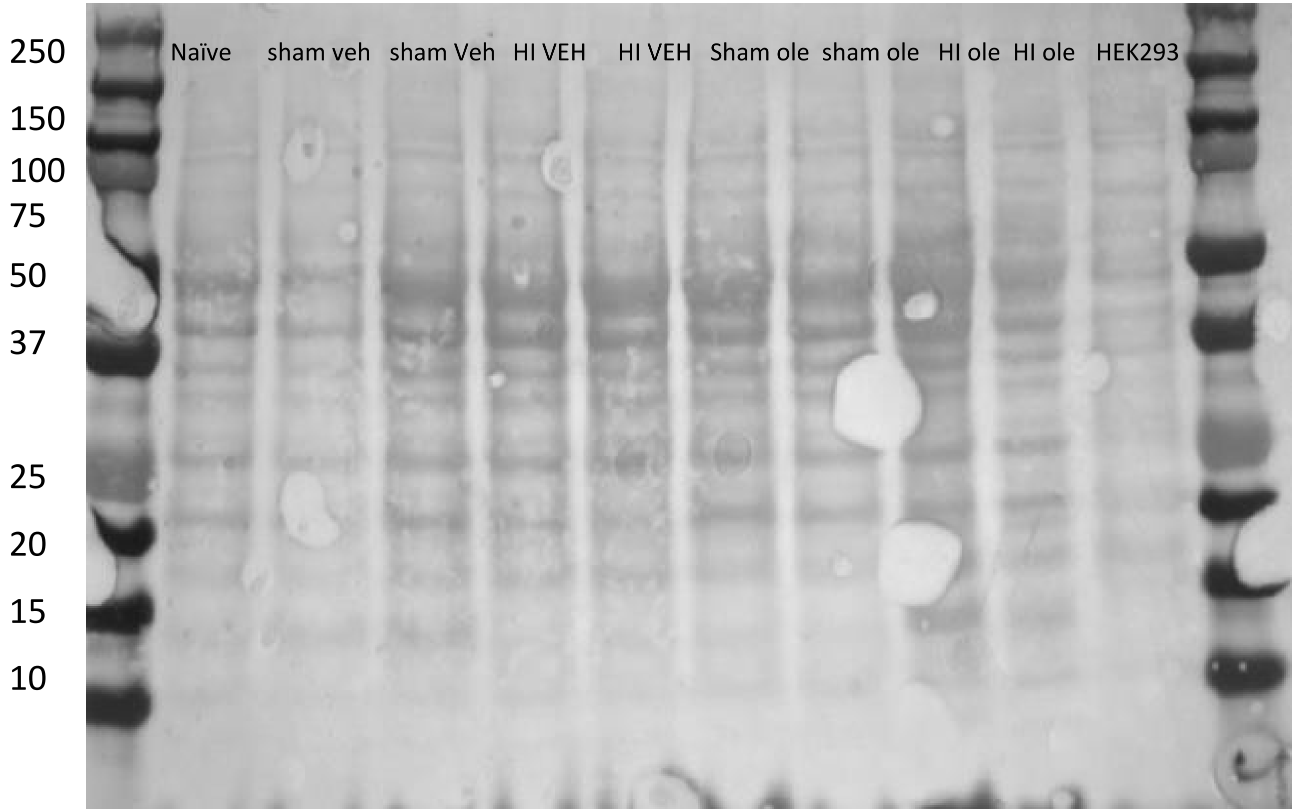

Proteiin load: WM 50 µg
Ab . Primary . PSMB5 . 1:1000

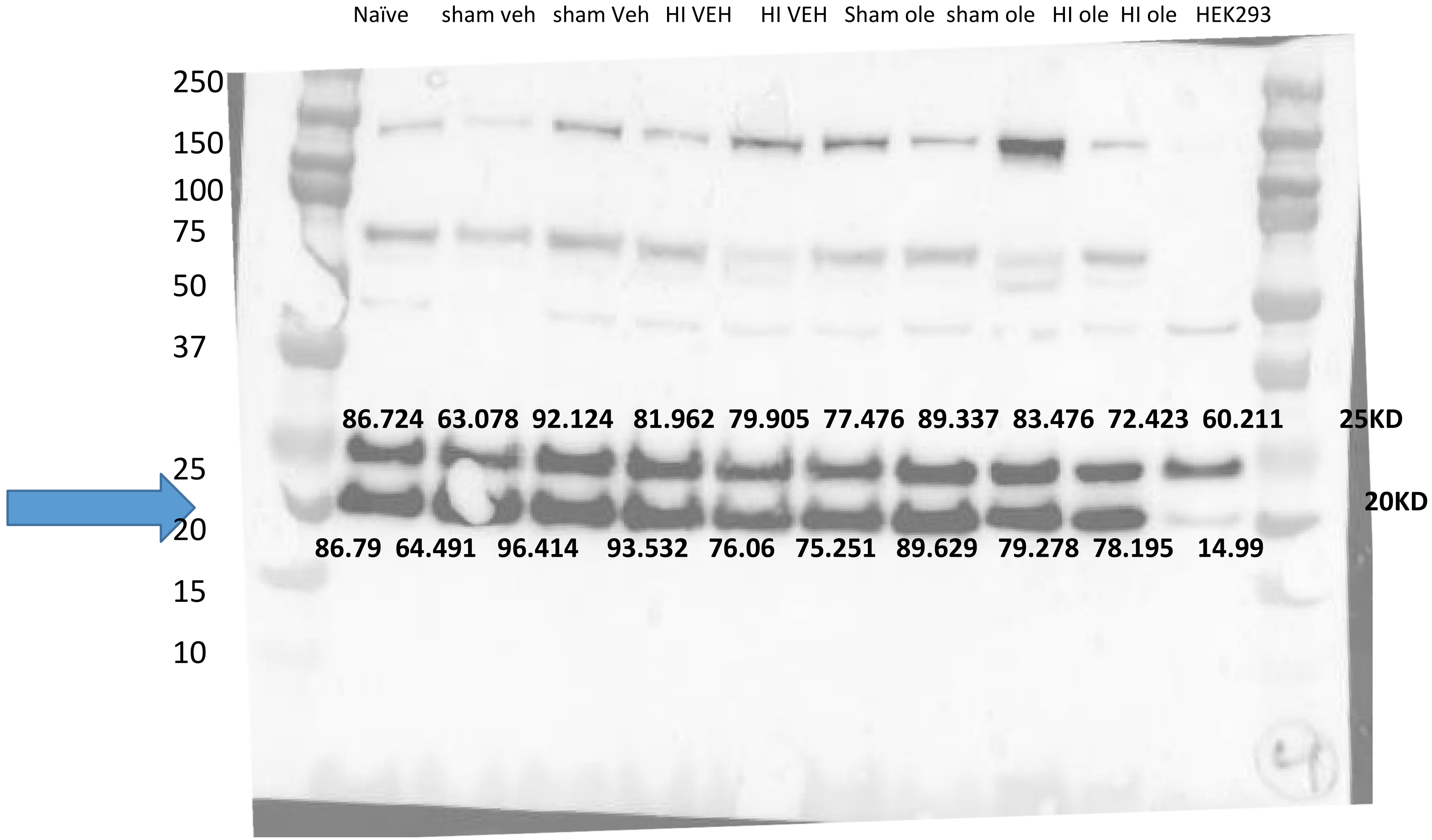

Ponceau membrane 5- SMC

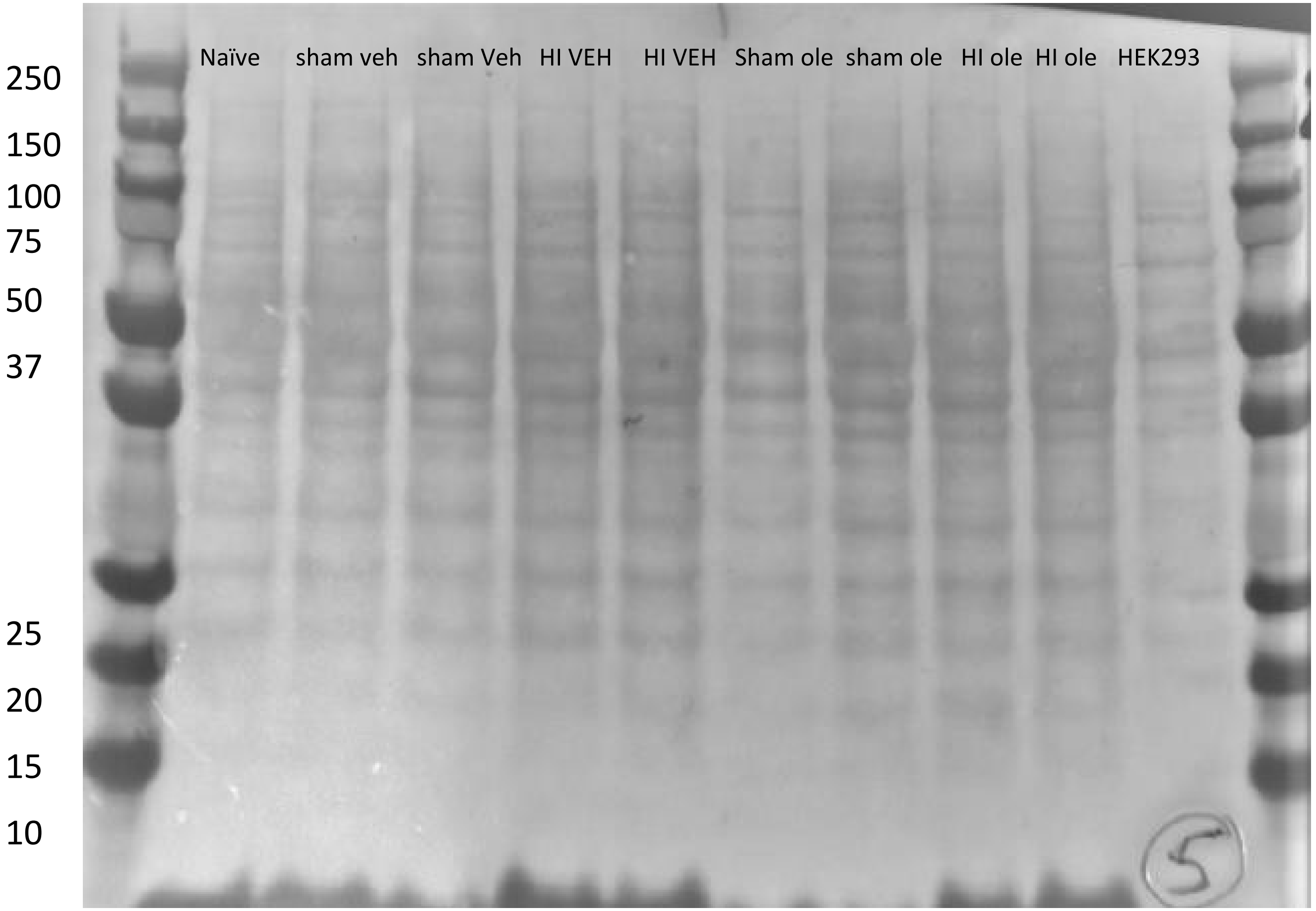

Protein load: SMC, 50 μg
Ab . Primary . PSMB5 . 1:1000

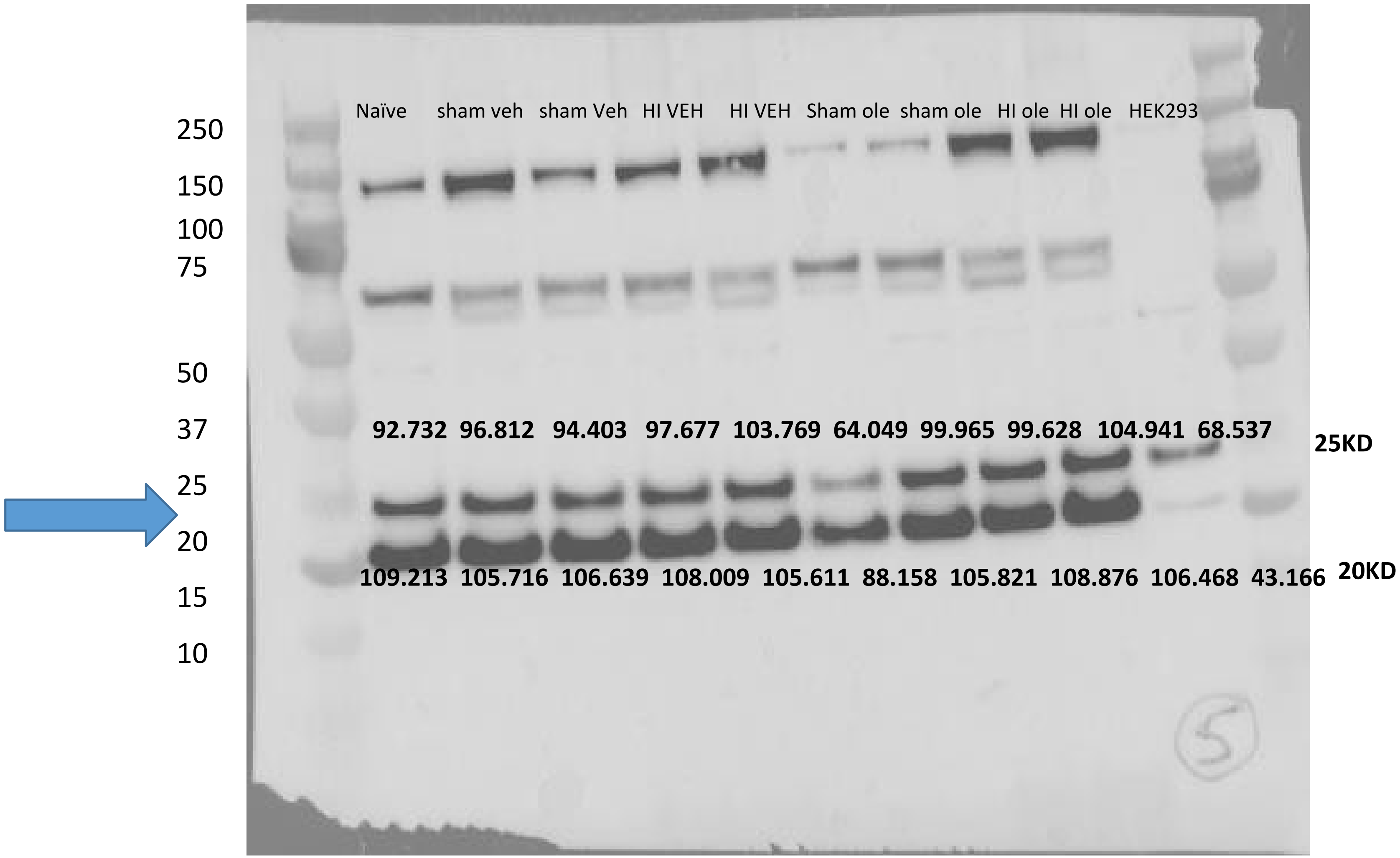

Ponceau membrane 6 WM

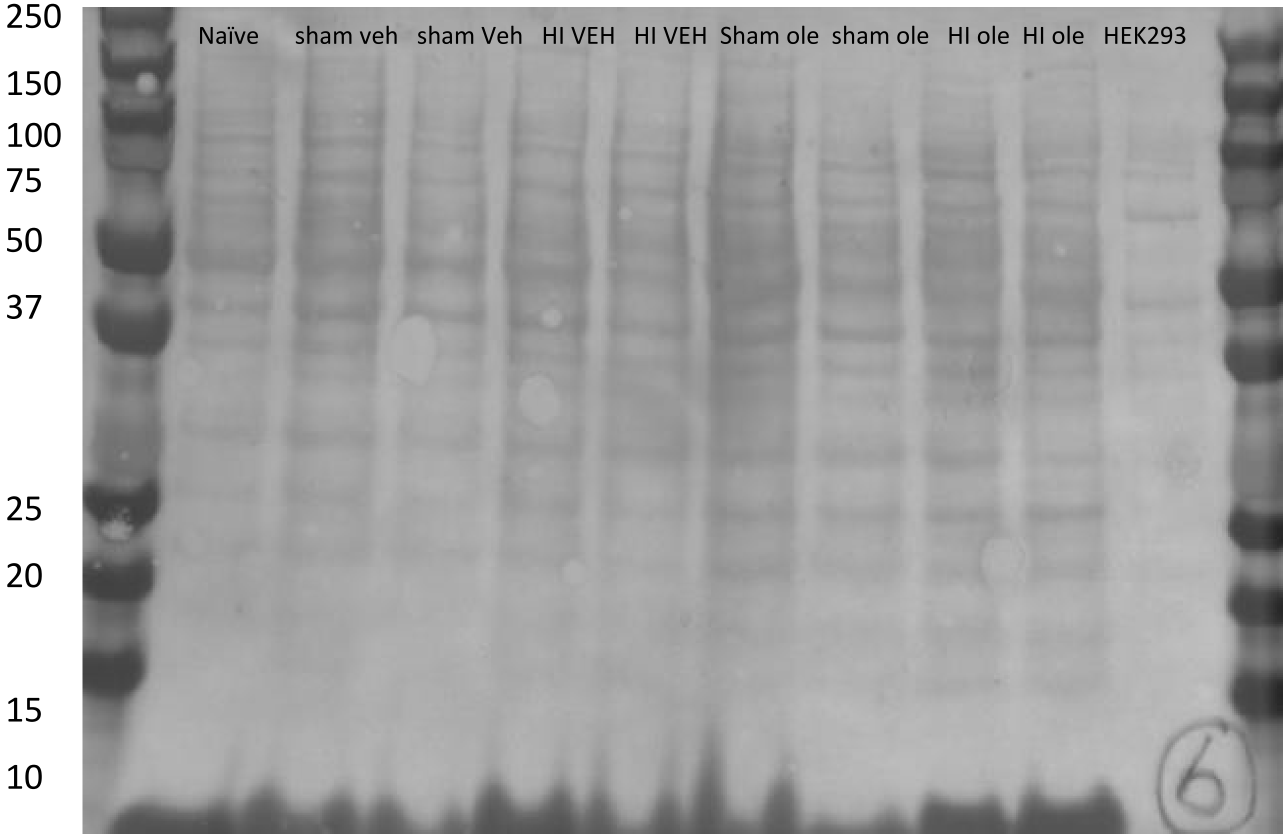

Protein load: WM, 50 μg
Ab . Primary . PSMB5 . 1:1000

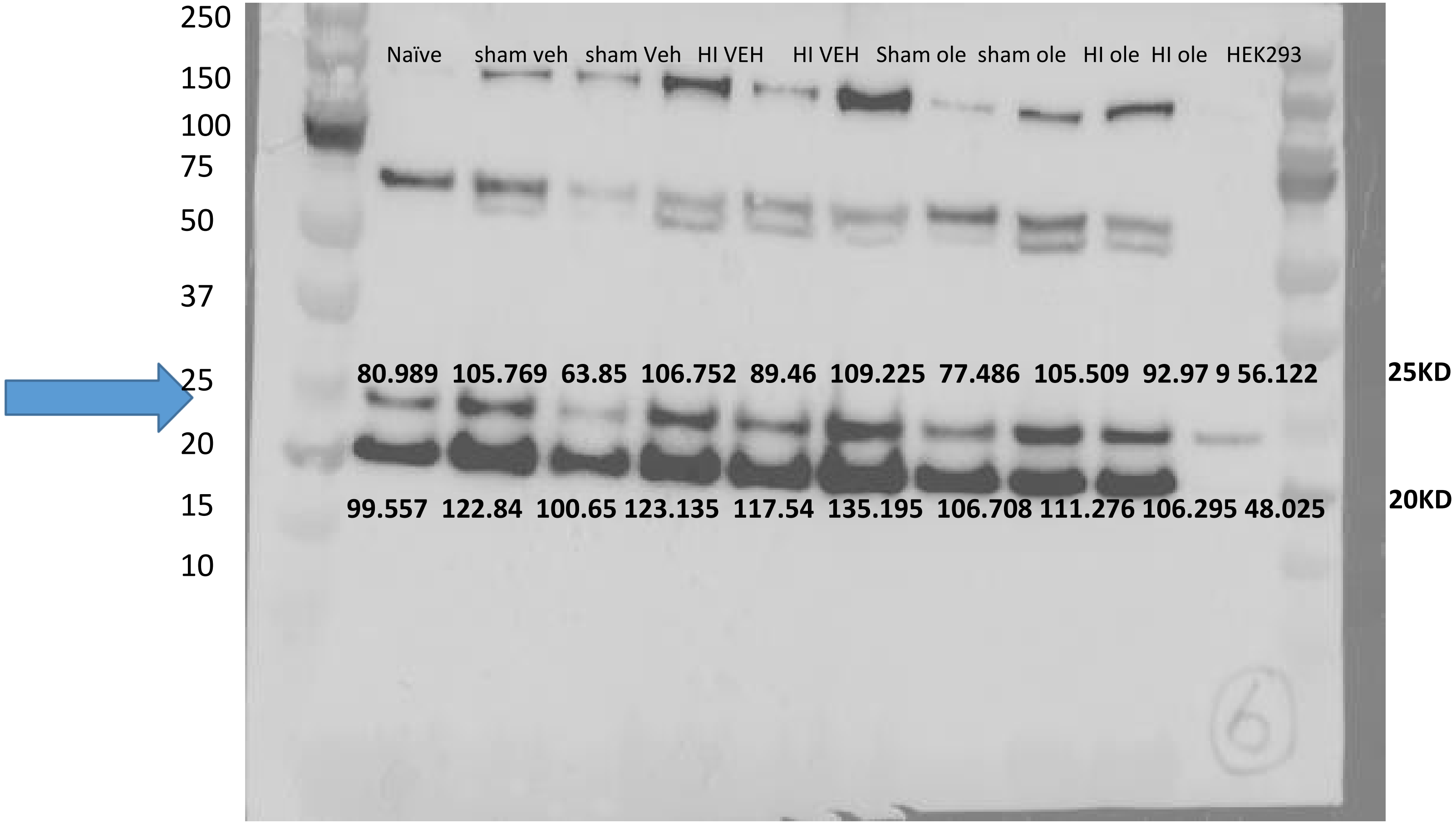

Ponceau membrane 7 SMC

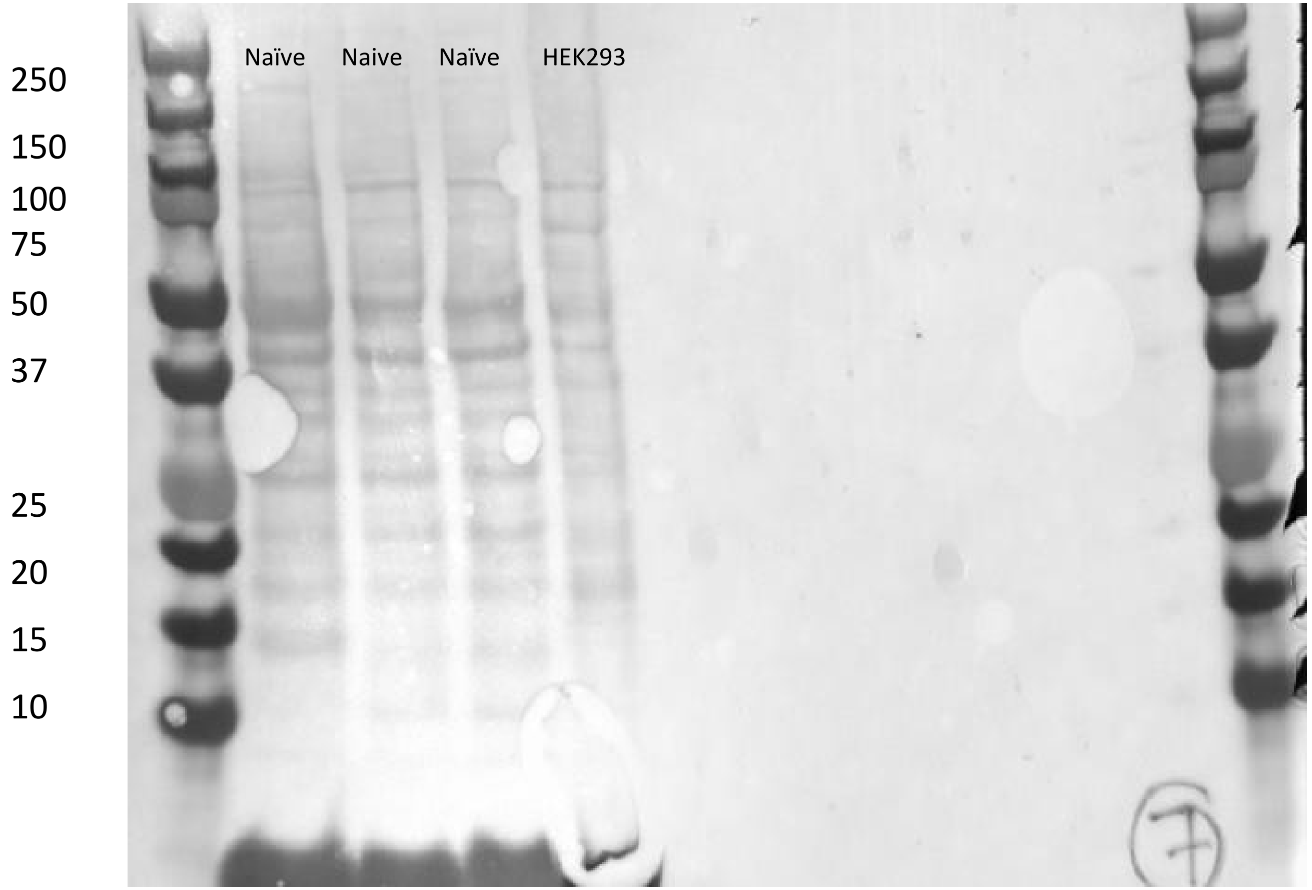

Protein load: SMC, 50 µg
Ab . Primary . PSMB5 . 1:1000

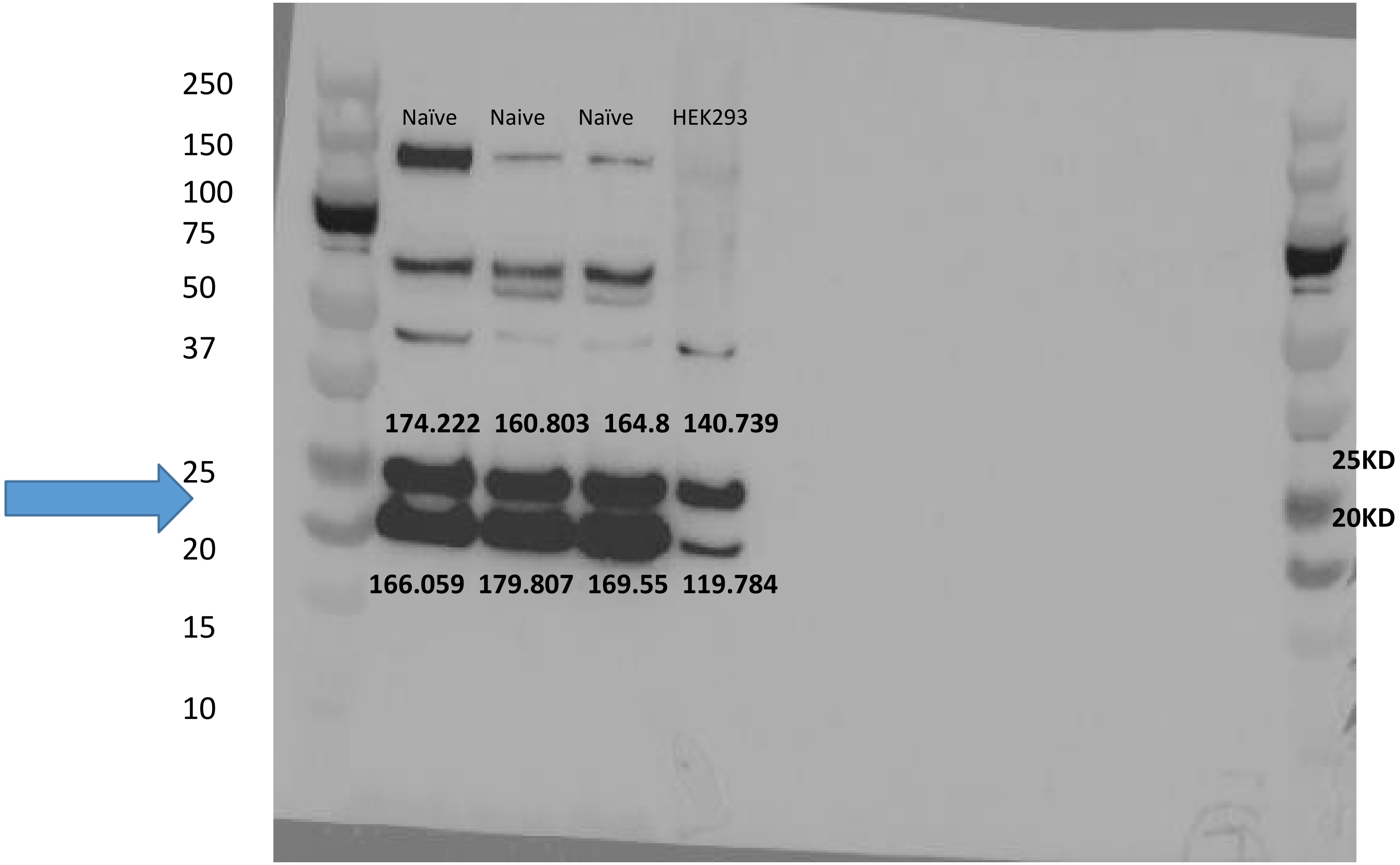

Ponceau membrane 8- WM

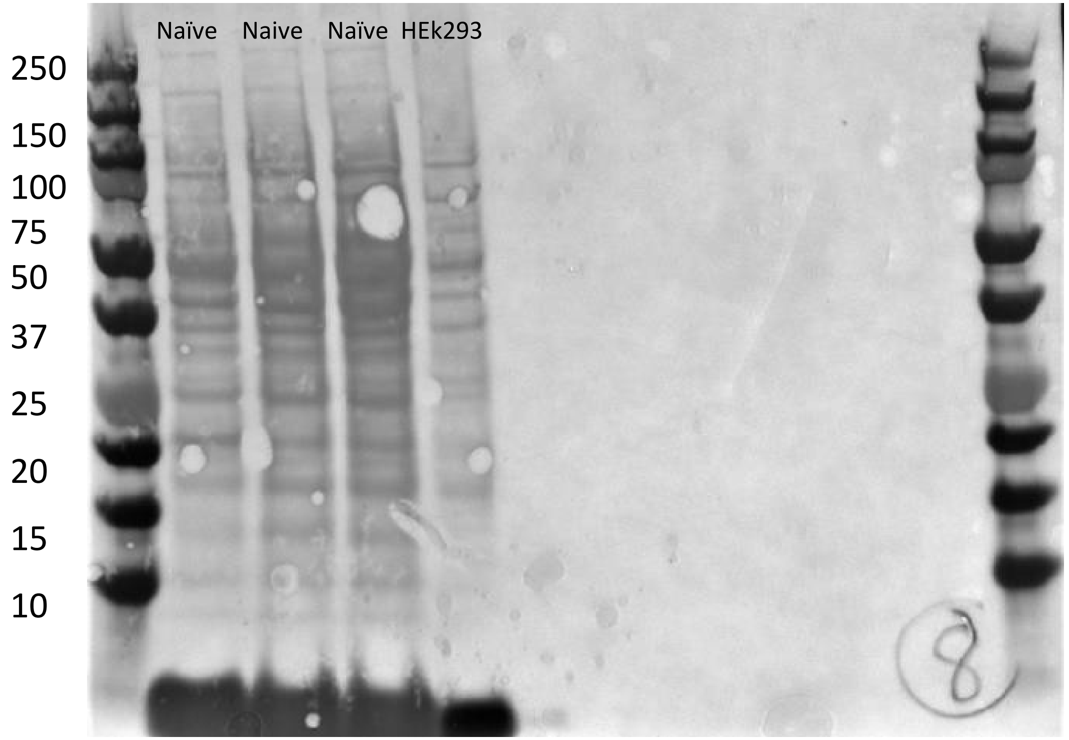

Protein load: WM, 50 µg
Ab . Primary . PSAB5 . 1:1000

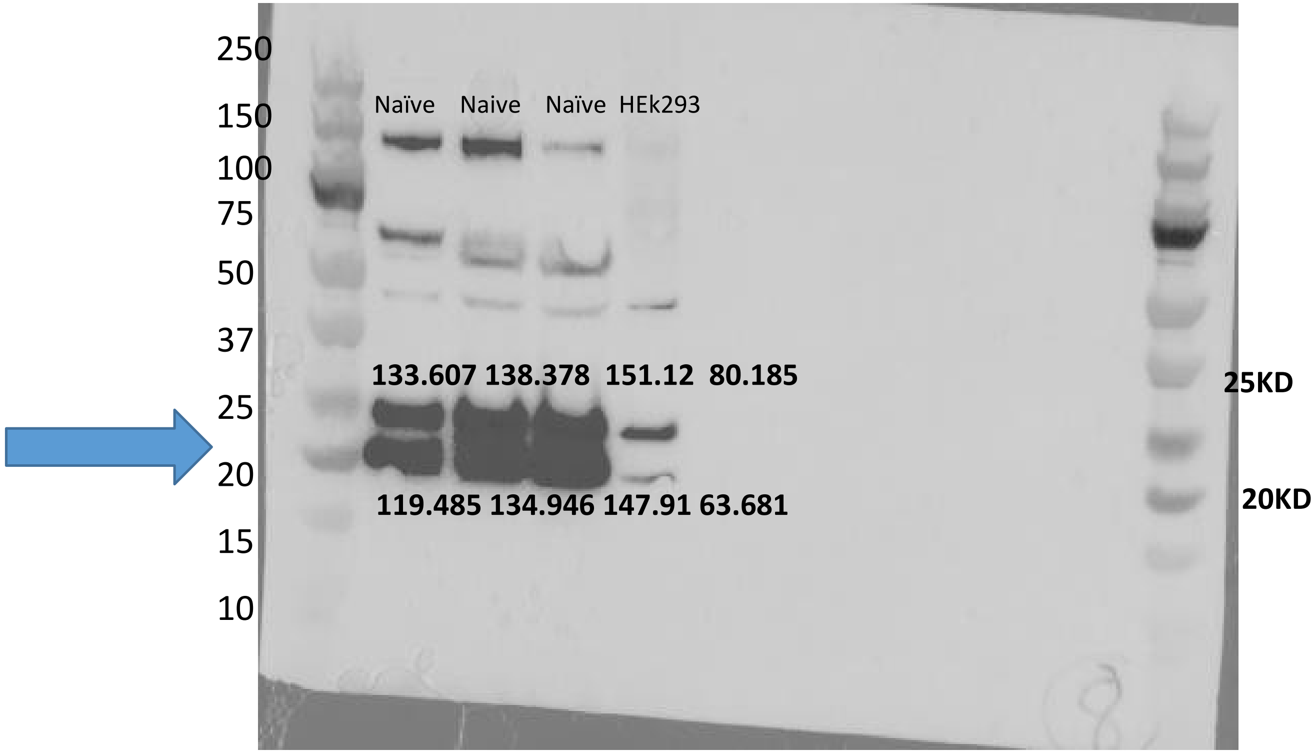

PSMA3
Full-Length Western blots

Poceau membrane 1- SMC

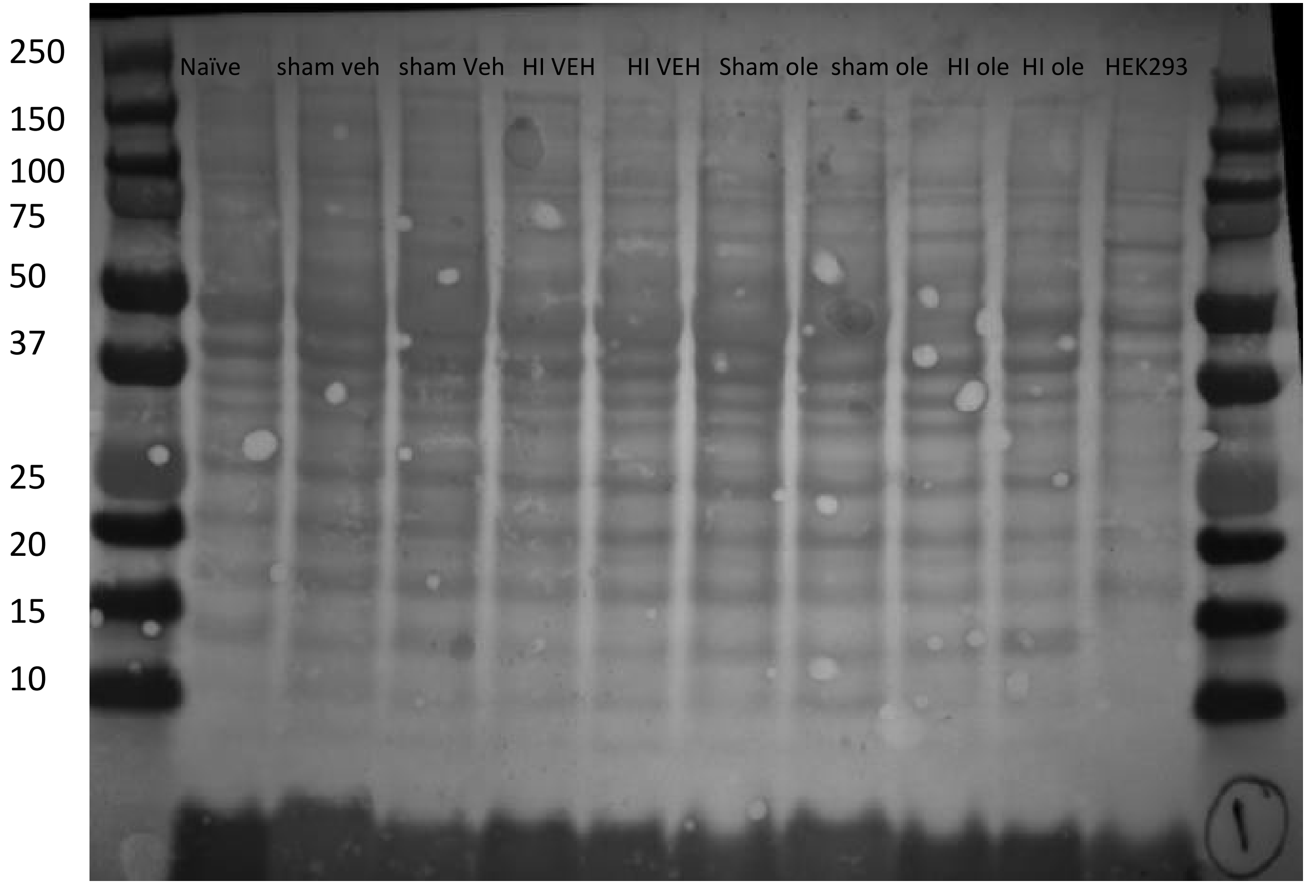

Protein load: SMC, 50 μg
Ab . Primary . PSMA3 . 1:1000

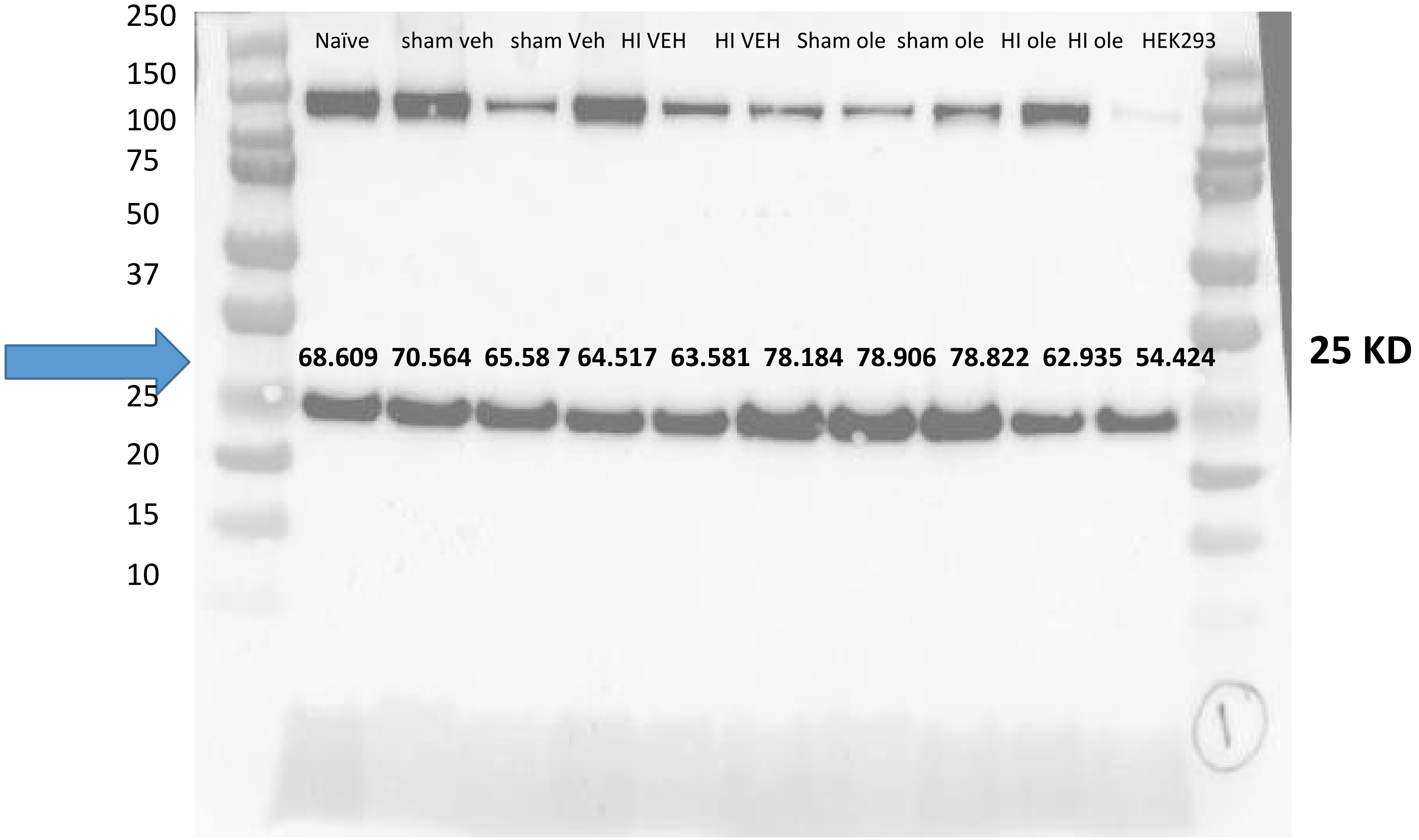

Ponceau membrane 2- WM

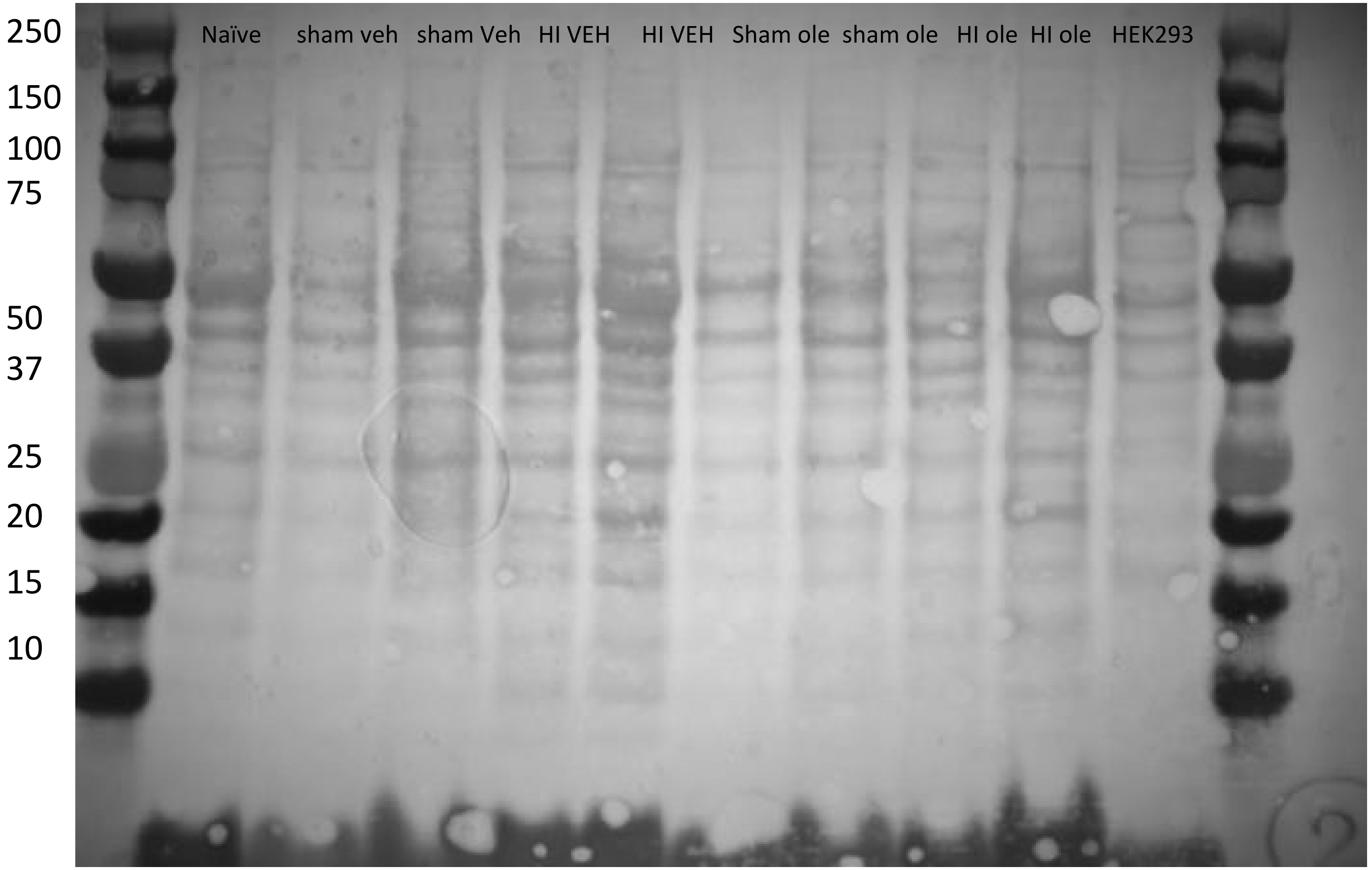

Protein load: WM, 50 µg
Ab . Primary . PSMA3 . 1:1000

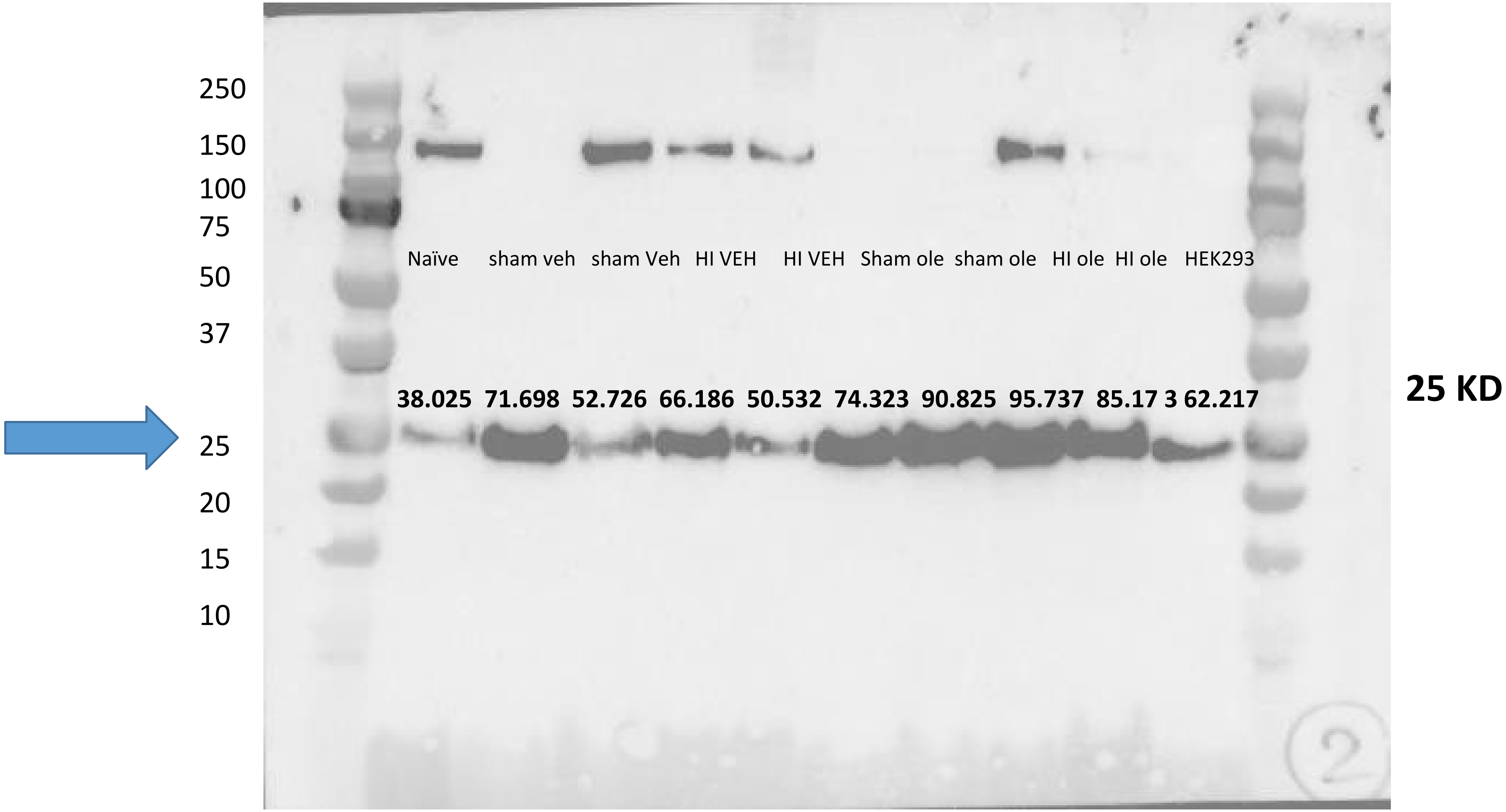

Ponceau membrane 3- SMC

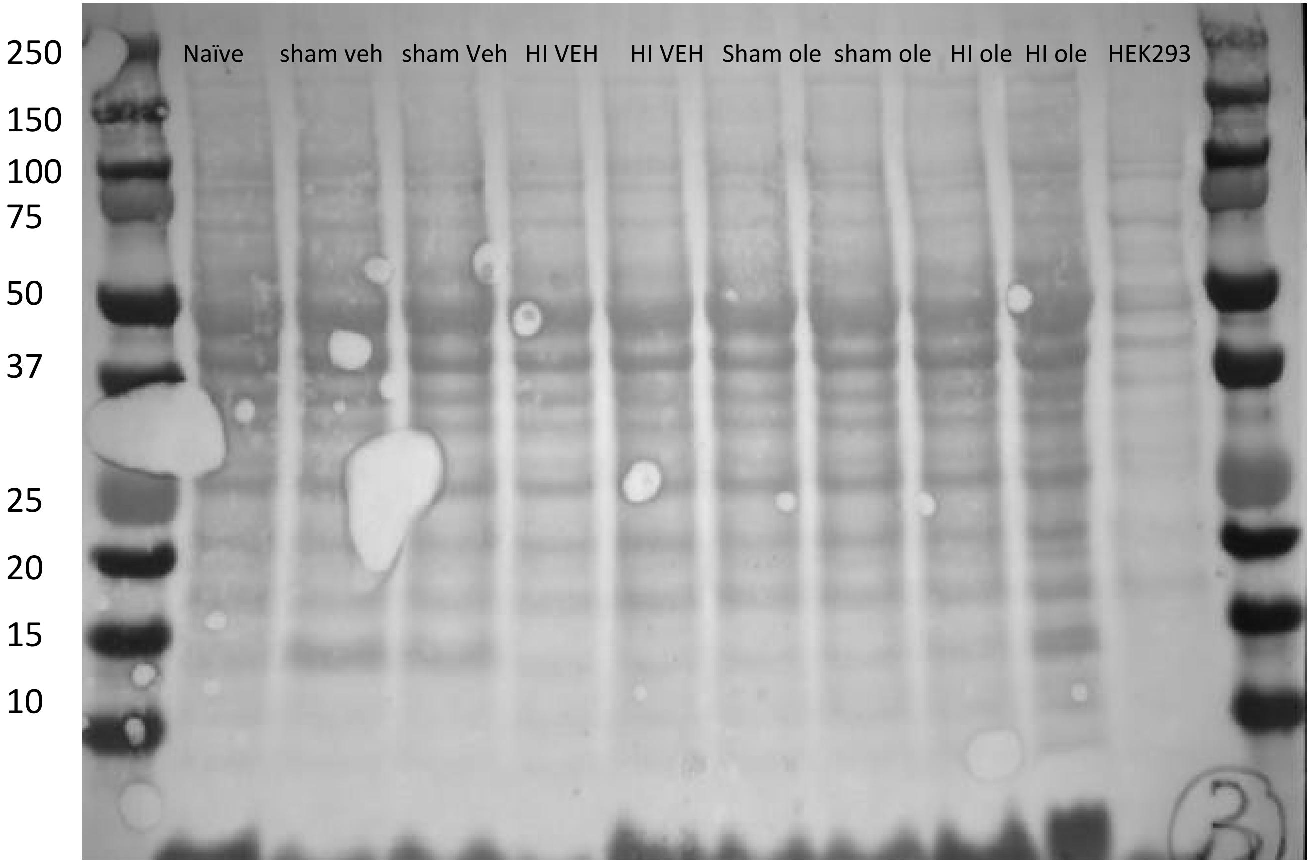

Protein load: SMC, 50 µg
Ab . Primary . PSMA3 . 1:1000

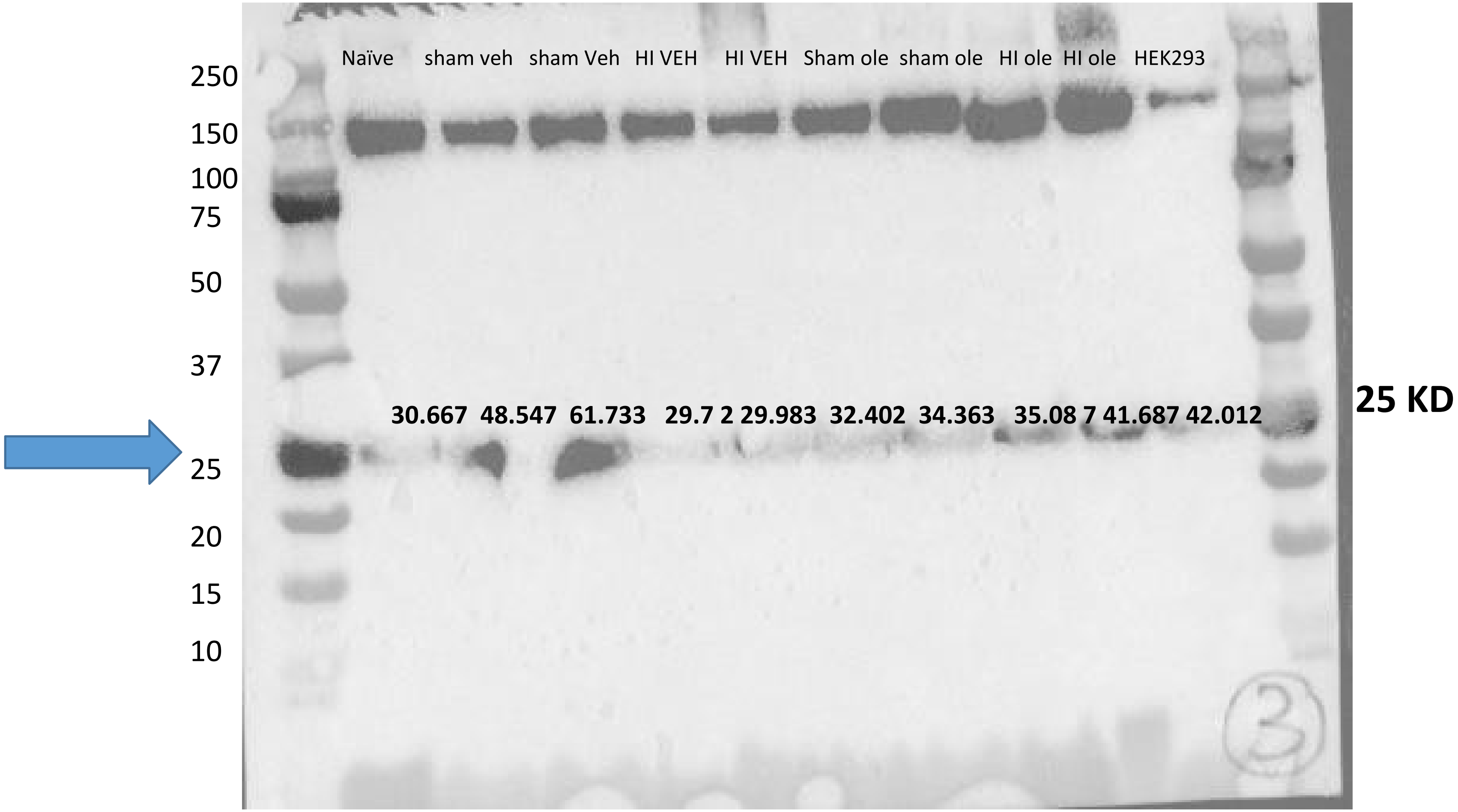

Ponceau membrane 4 - WM

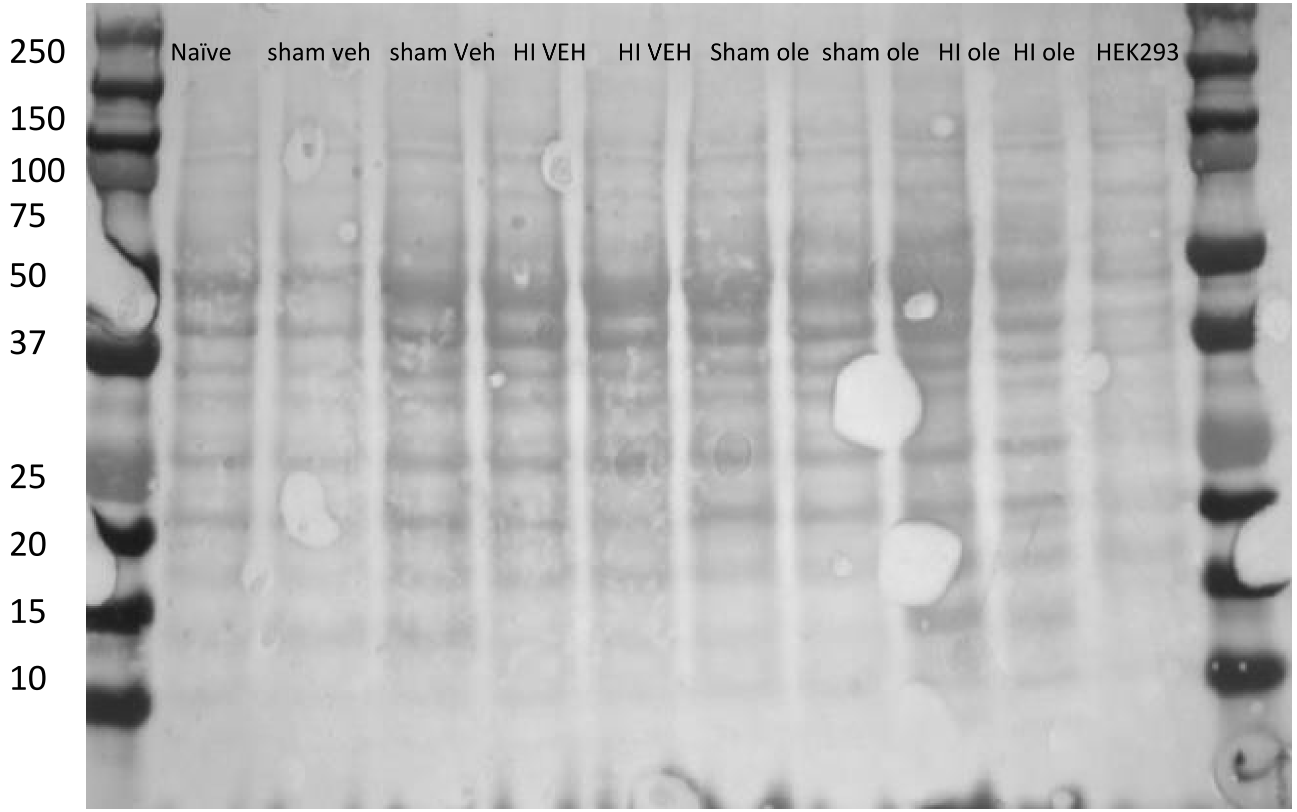

Protein load: WM, 50 µg
Ab . Primary . PSMA3 . 1:1000

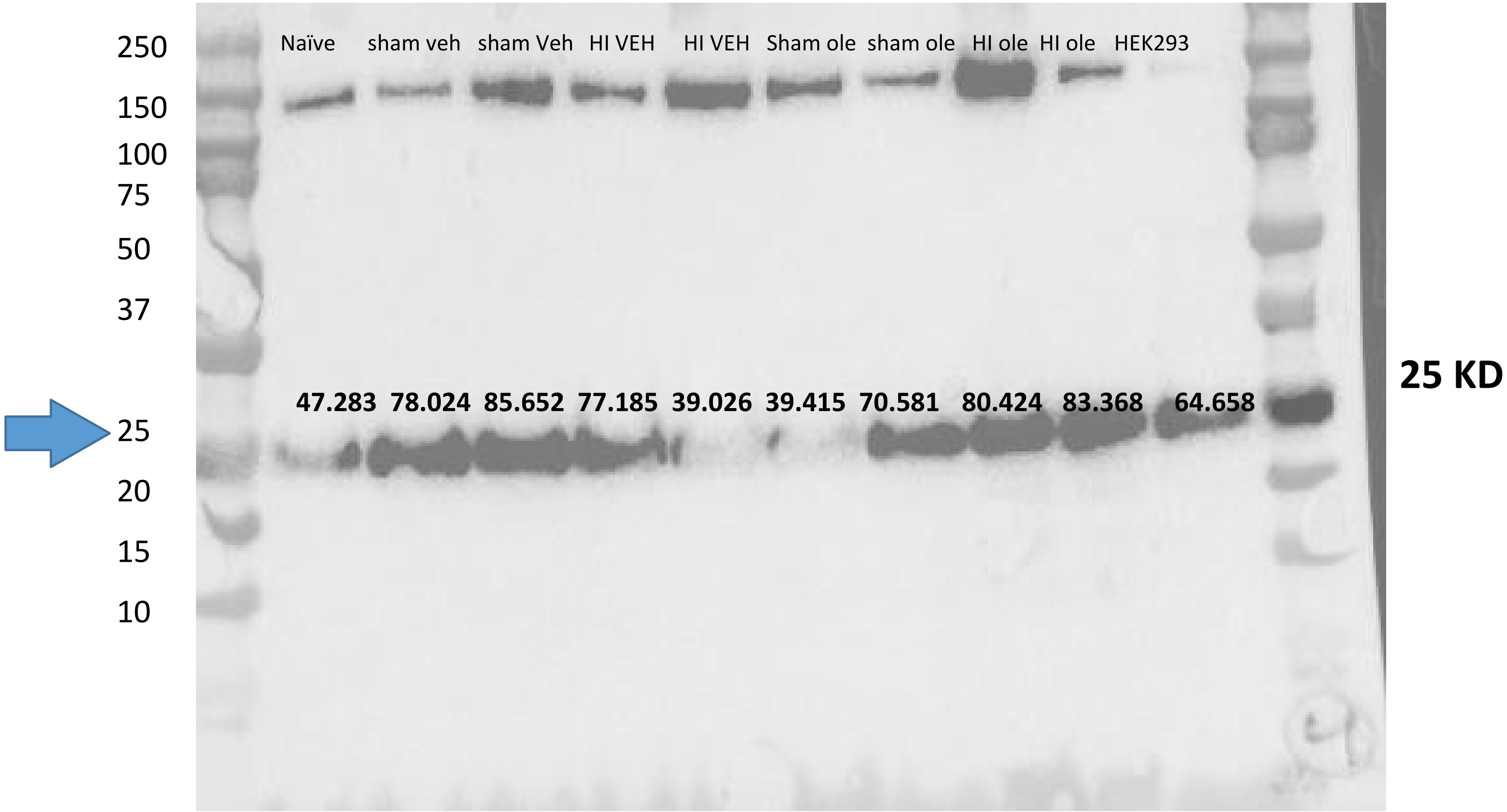

Ponceau membrane 5- SMC

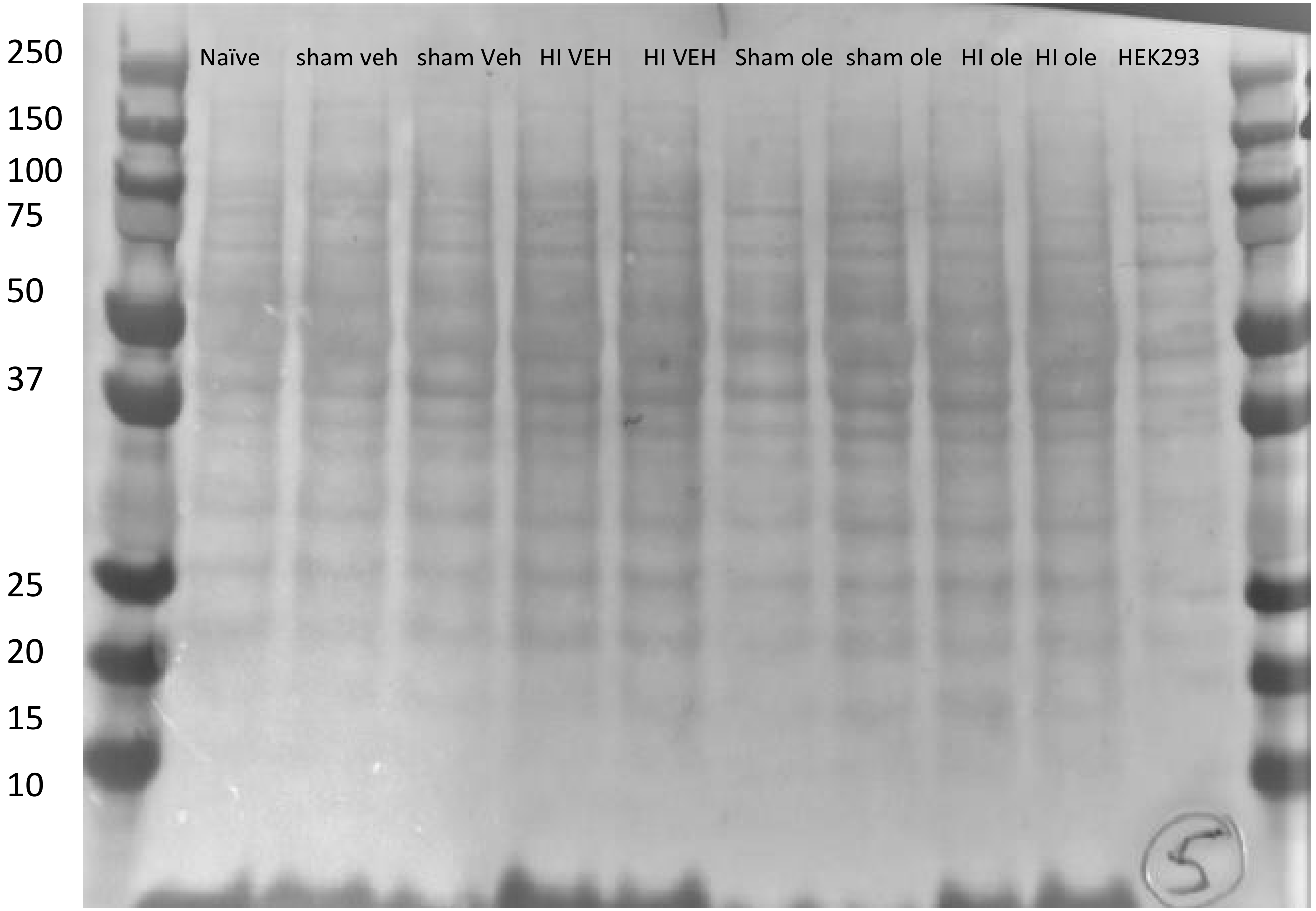

Protein load: SMC, 50 µg
Ab . Primary . PSMA3 . 1:1000

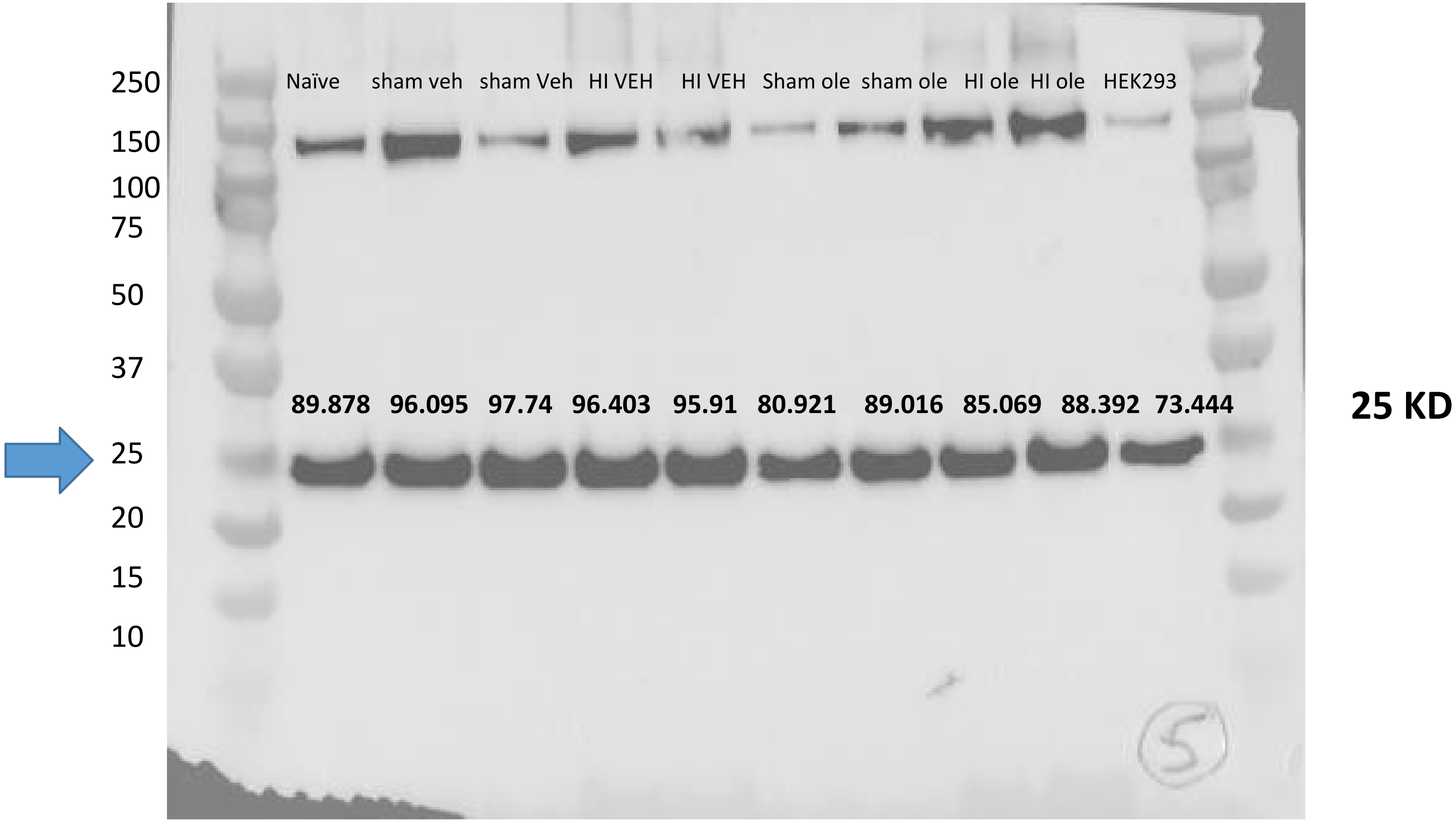

Ponceau membrane 6 WM

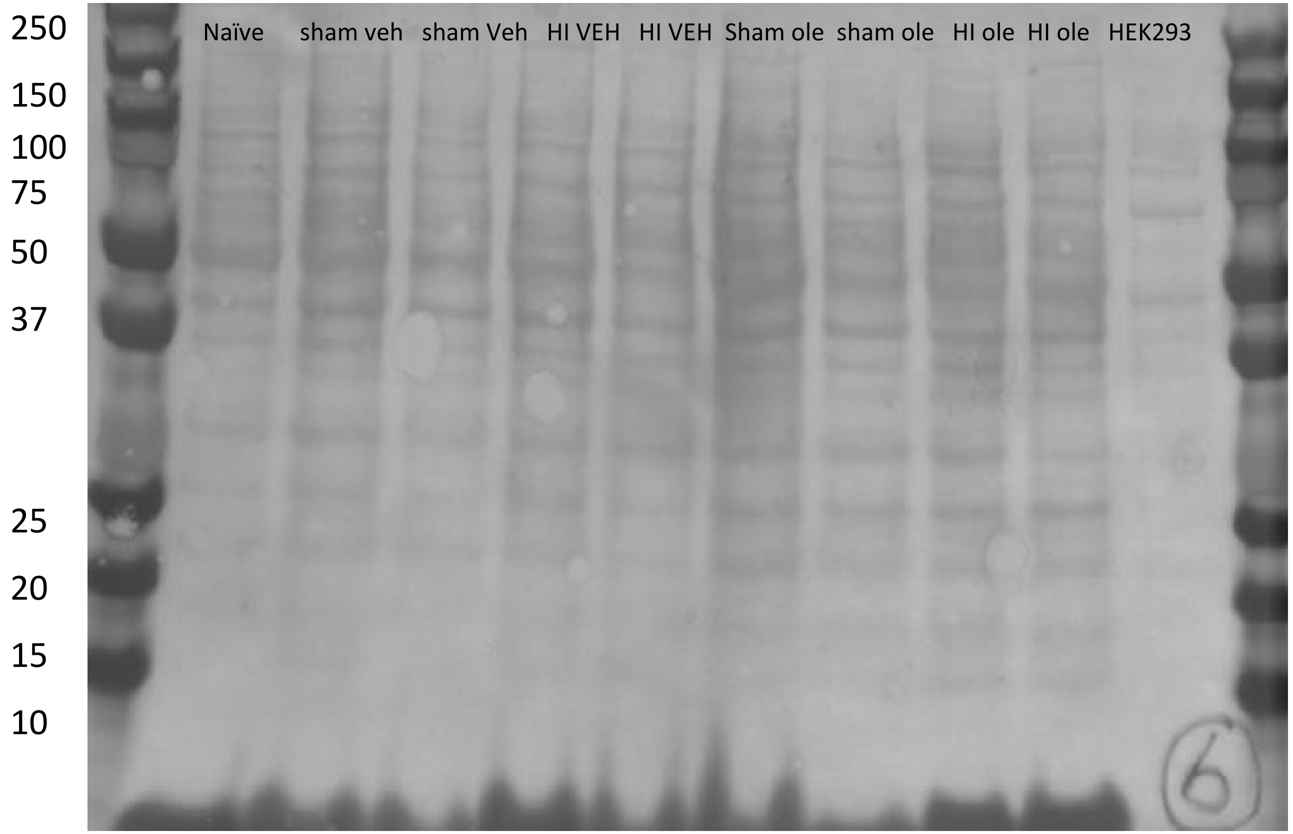

Protein load: WM, 50µg
Ab . Primary . PSMA3 . 1:1000

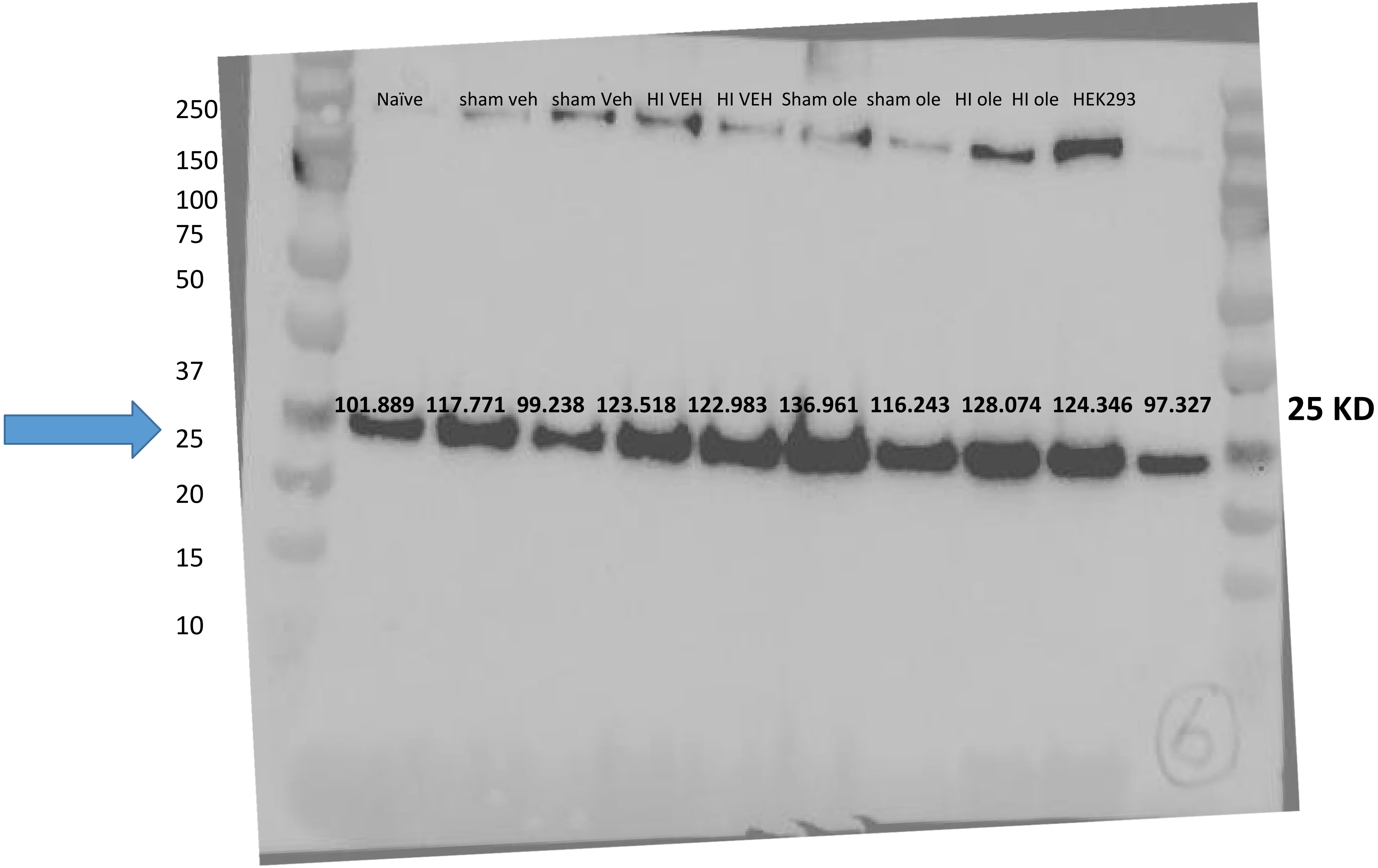

Ponceau membrane 7 SMC

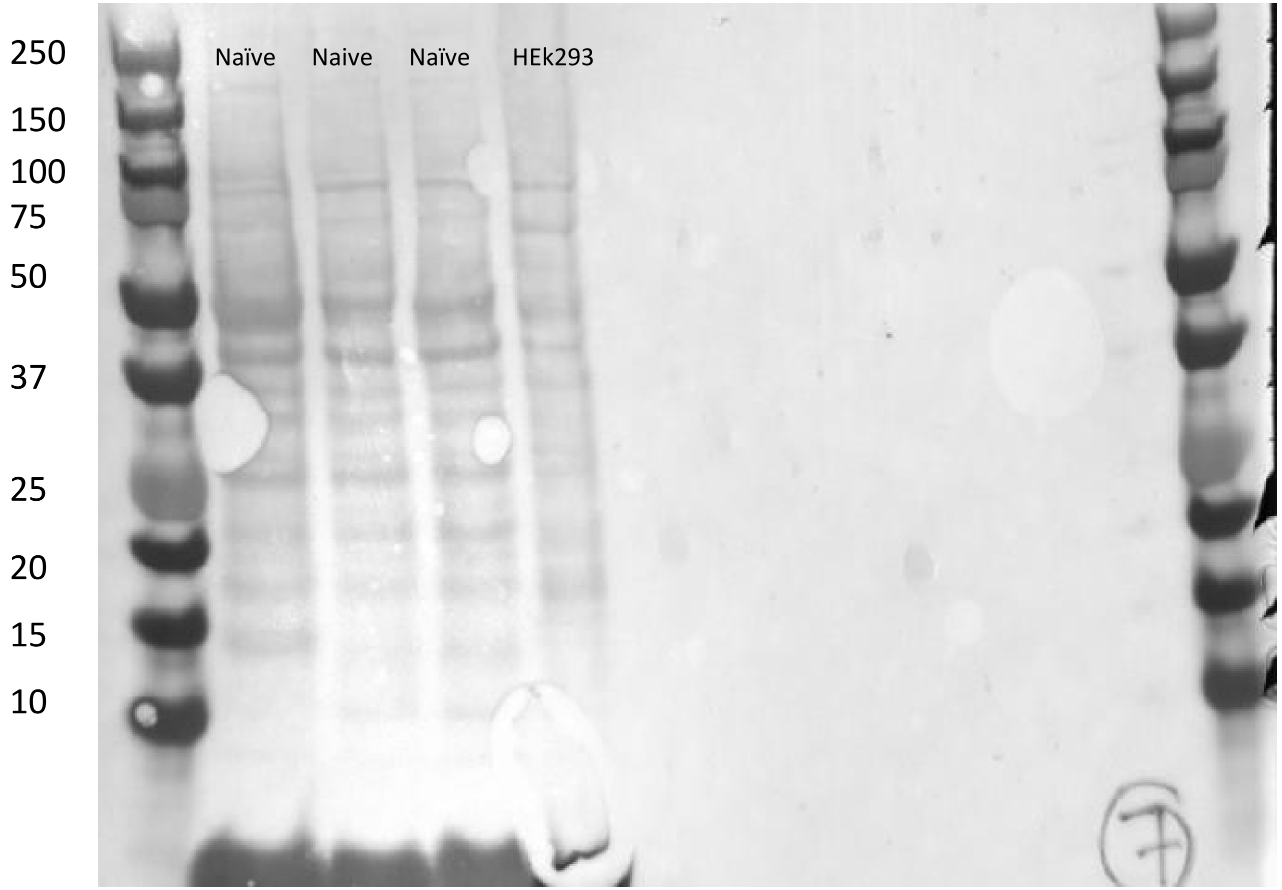

Protein load: SMC, 50 µg
Ab . Primary . PSMA3 . 1:1000

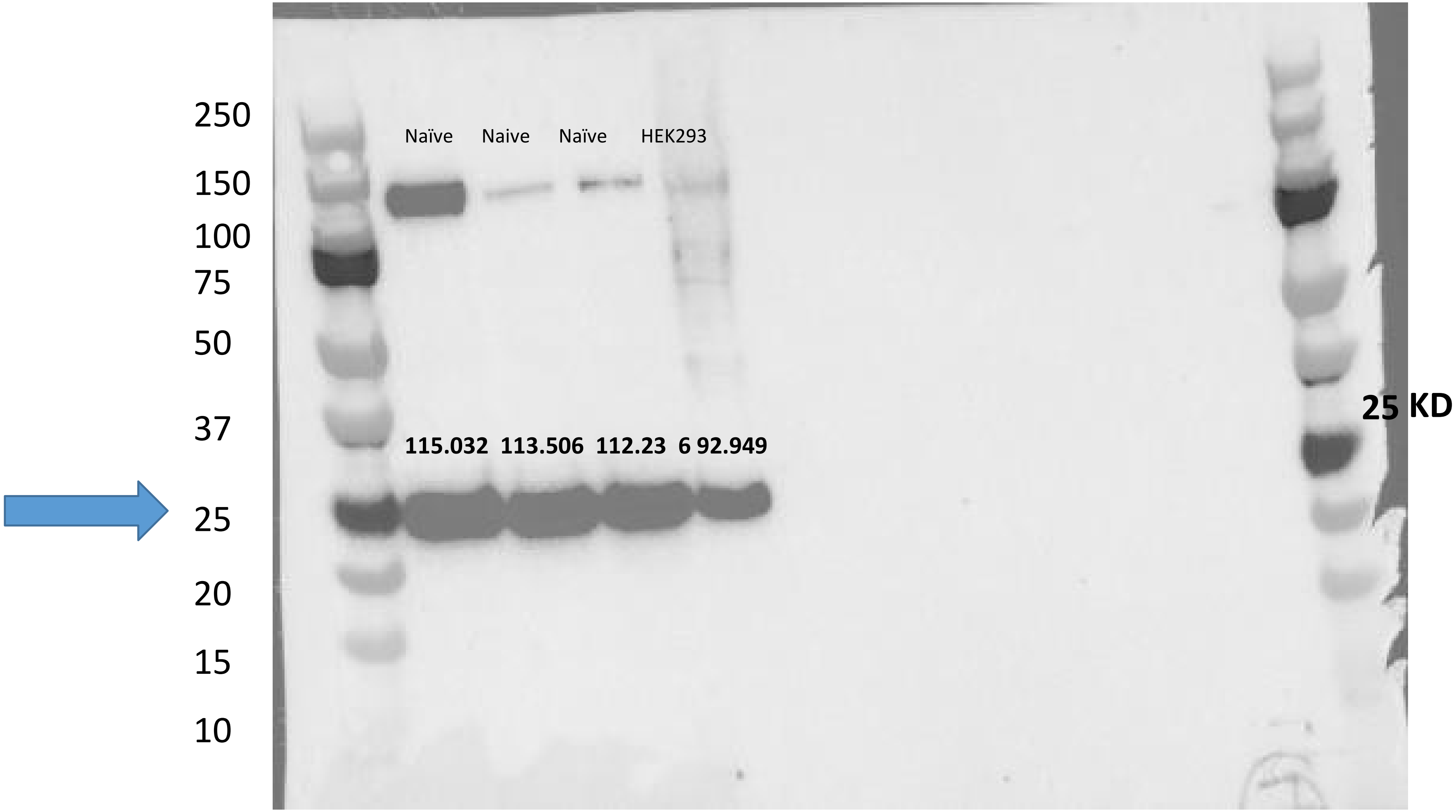

Ponceau membrane 8- WM

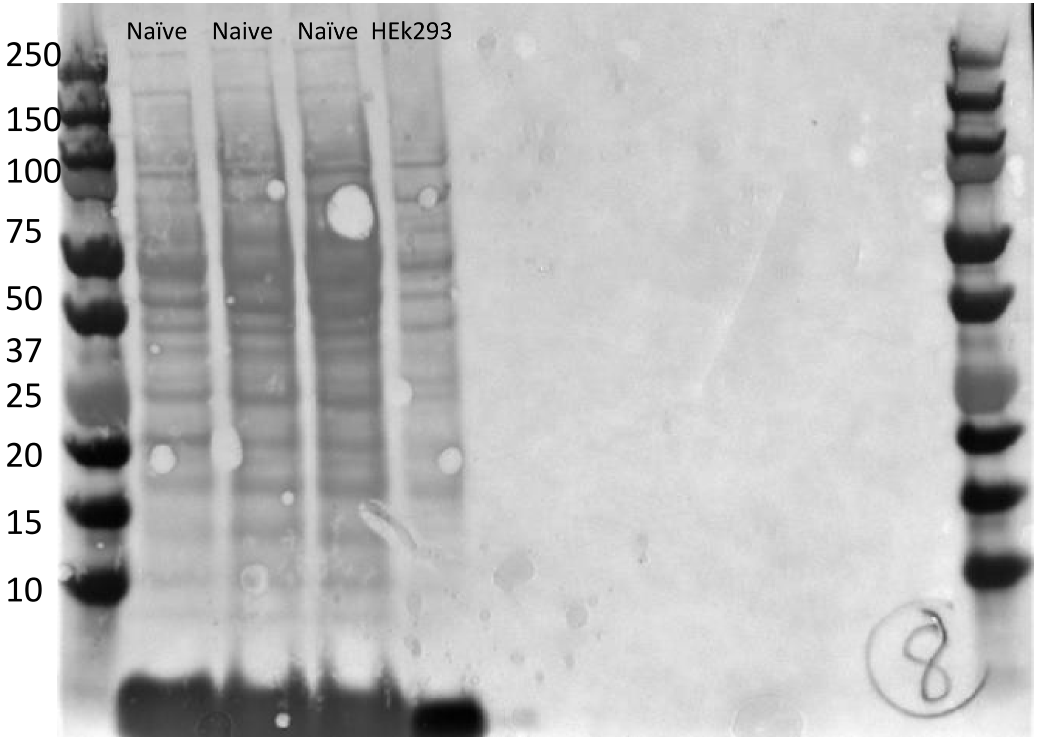

Protein load: WM, 50 µg
Ab . Primary . PSMA3 . 1:1000

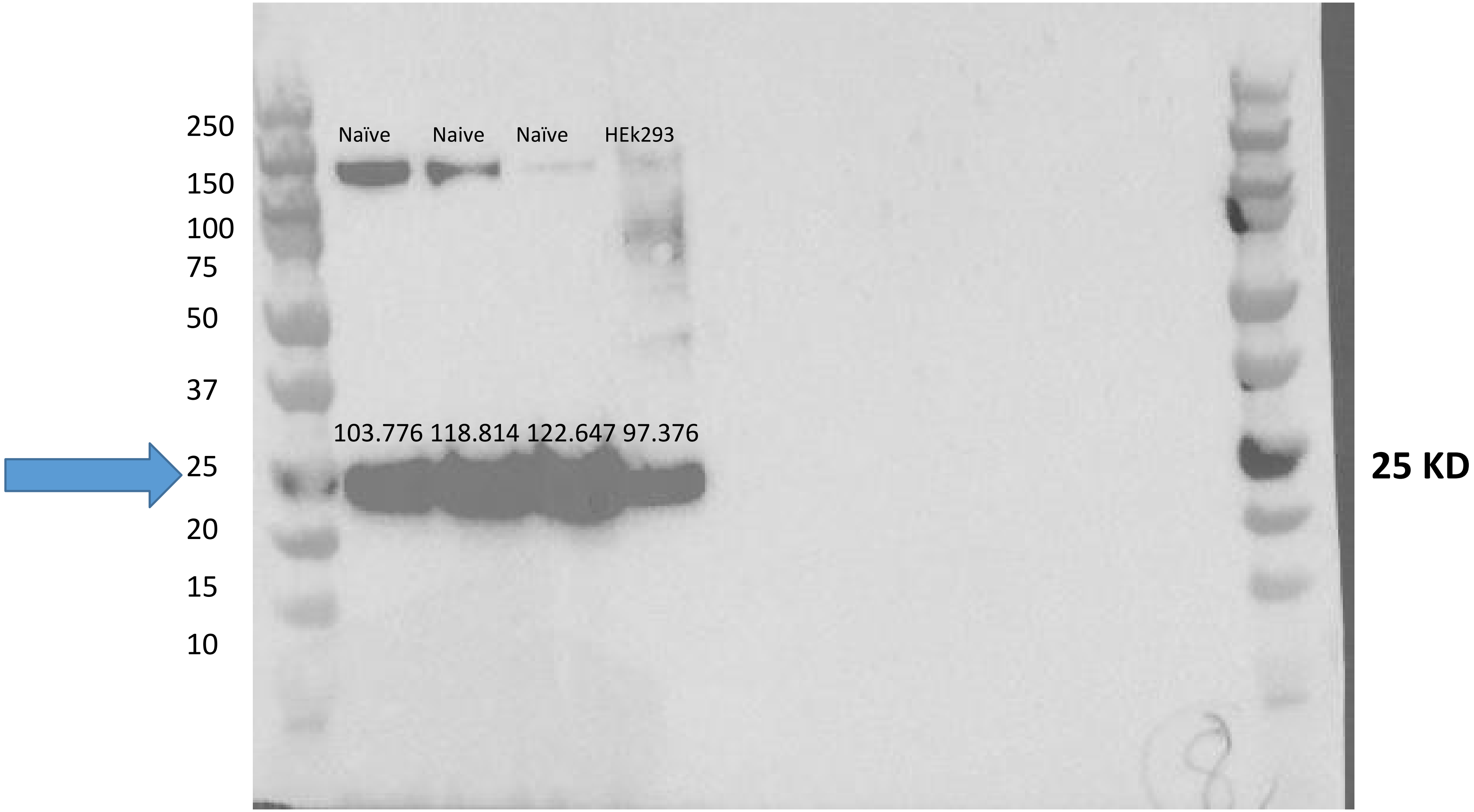

SOD2
Full-Length Western blots

Membrane 1- Ponceau

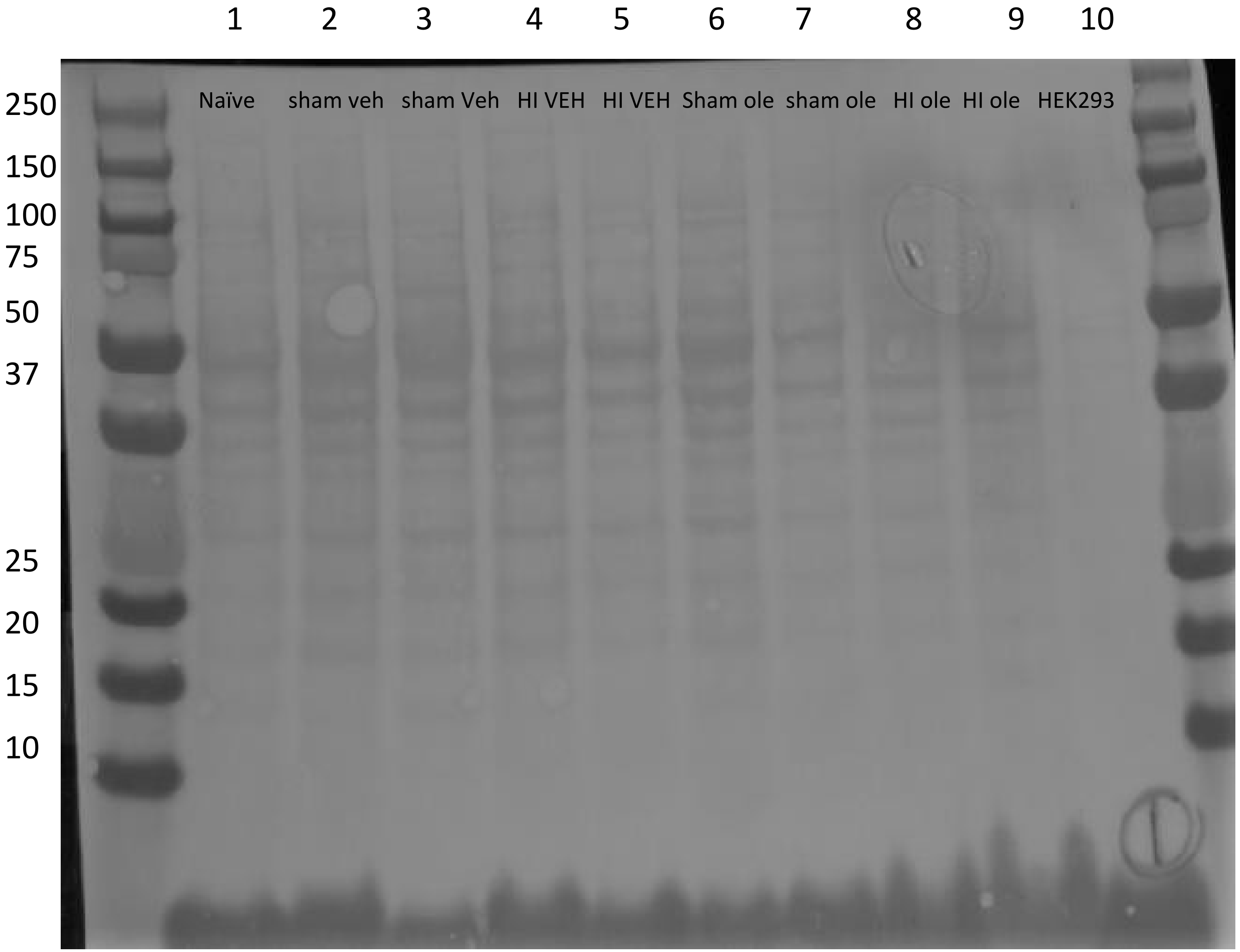

Protein load: SMC, **10 µg**
Ab . Primary . SOD2 ENZO. 1:1500

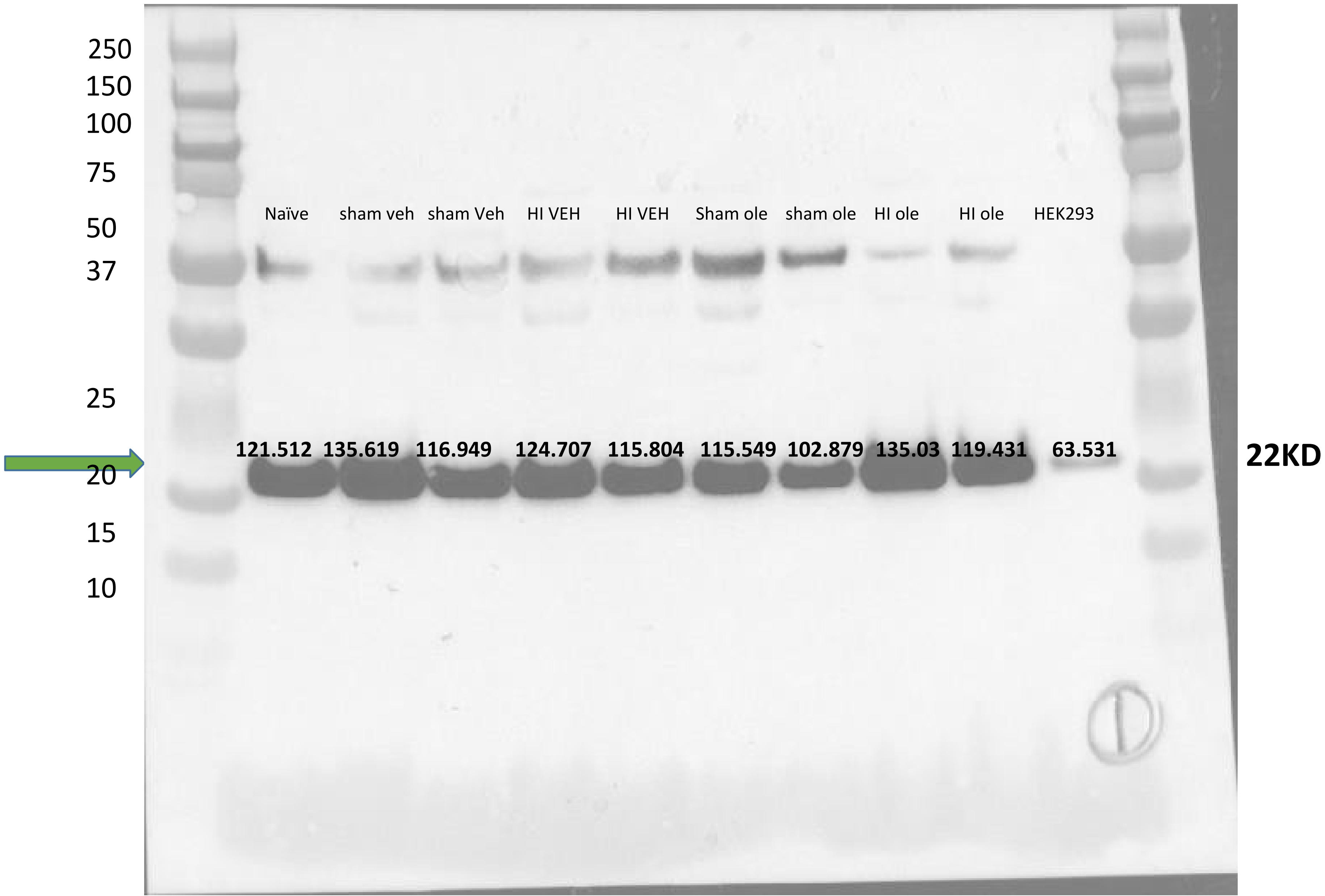

Membrane 2- Ponceau

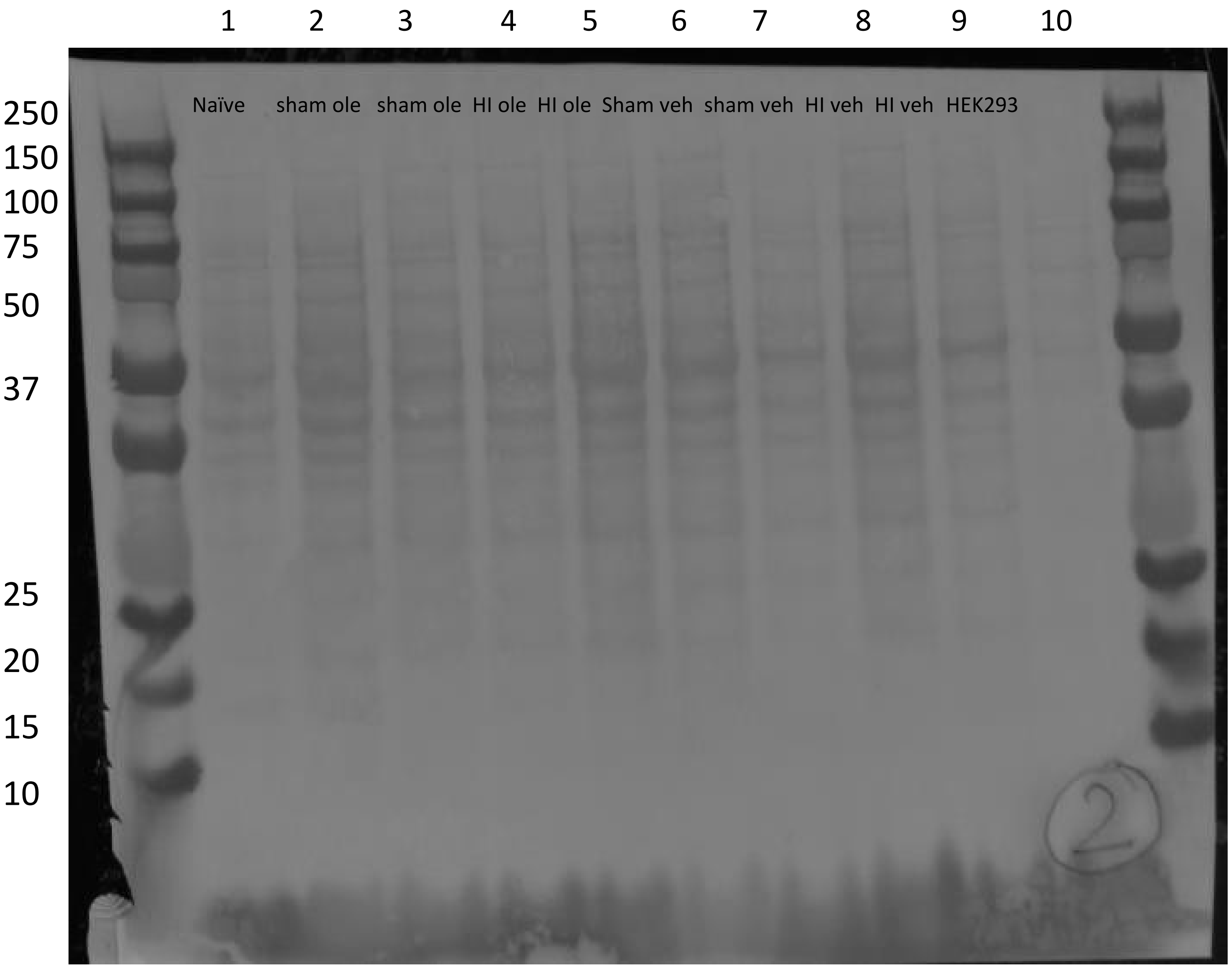

Protein load: SMC, **10 µg**
Ab . Primary . SOD2 ENZO.

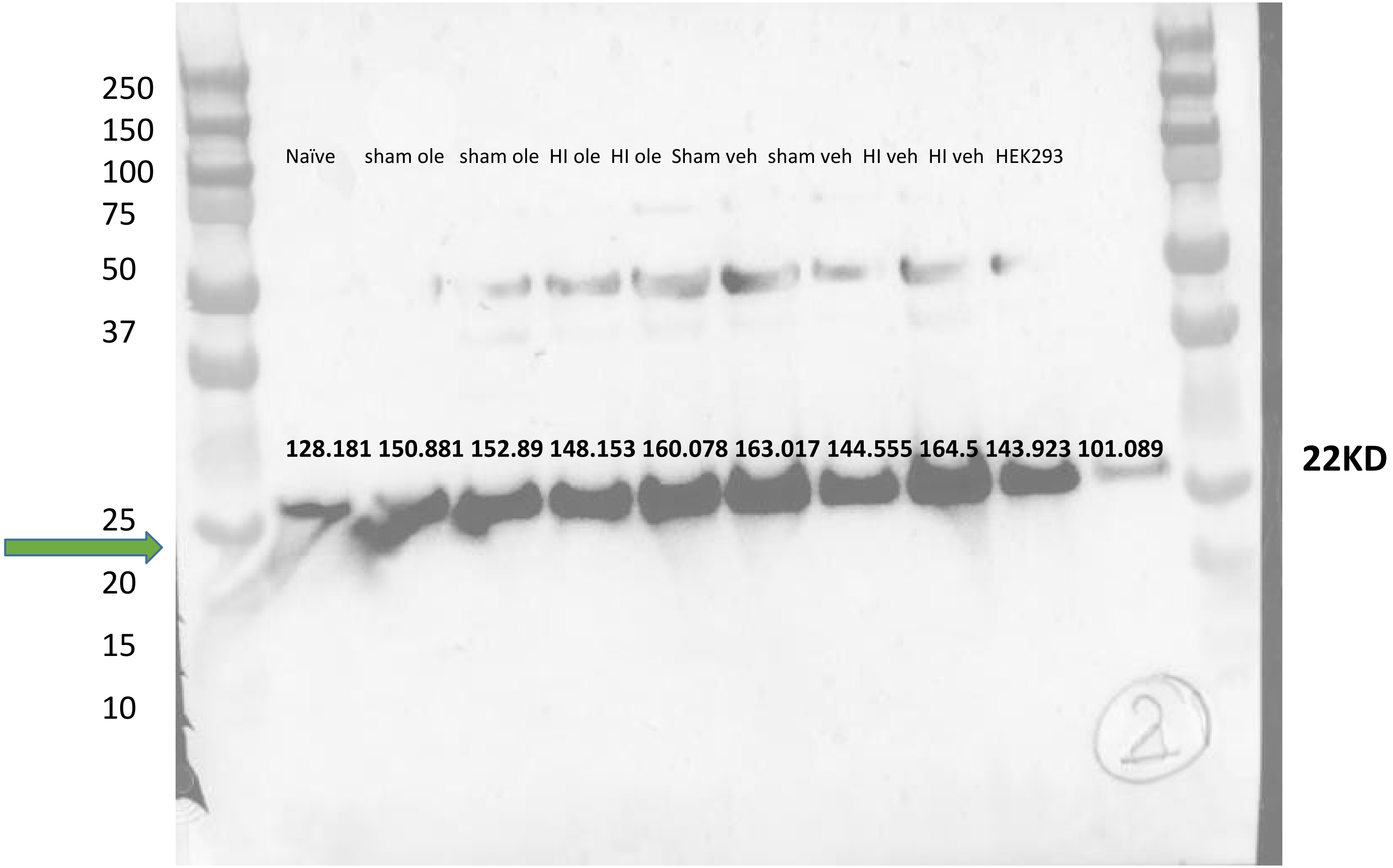

Membrane 3- Ponceau

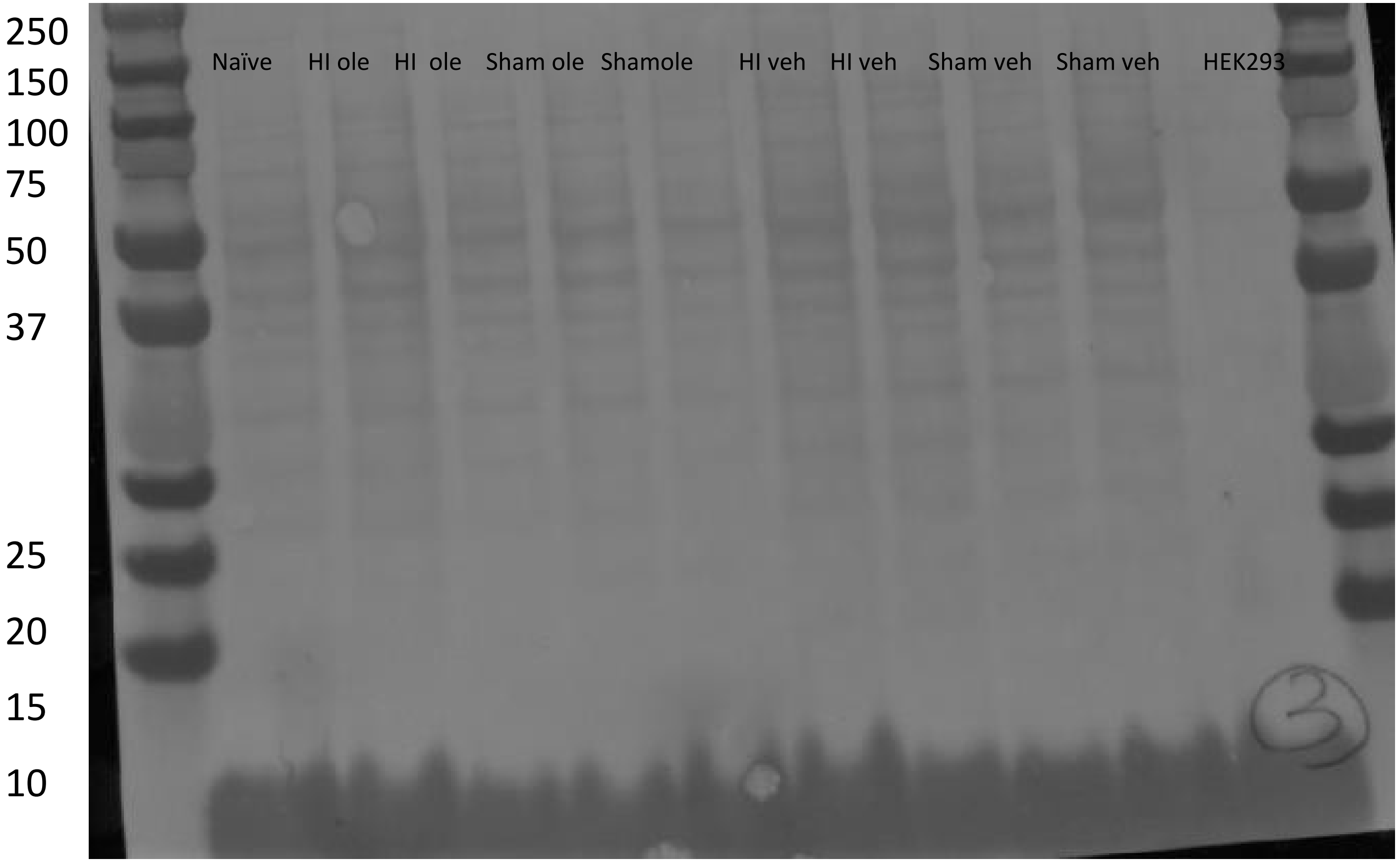

Protein load: SMC, **10 µg**
Ab . Primary . SOD2 ENZO. 1:1500

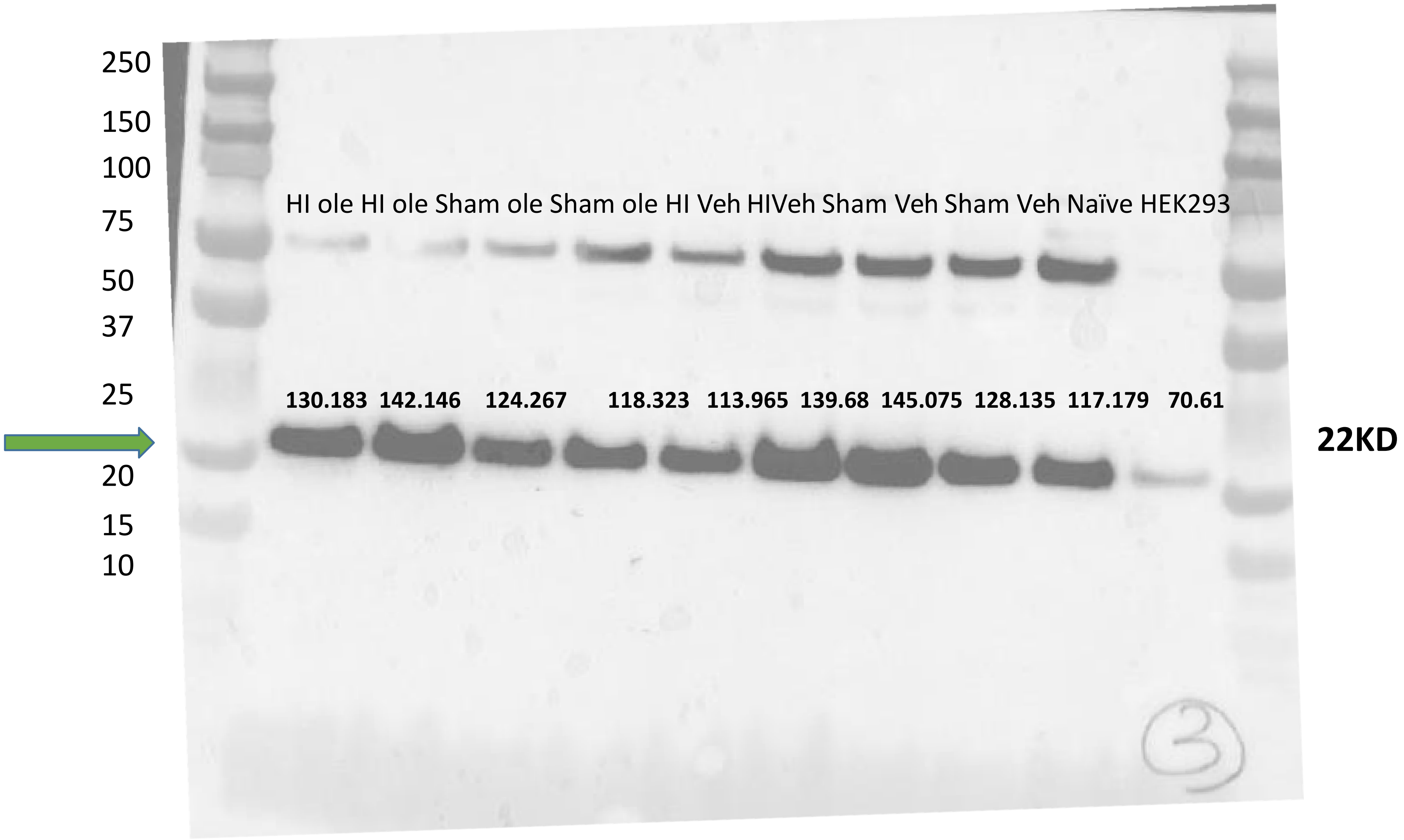

Membrane 4- Ponceau

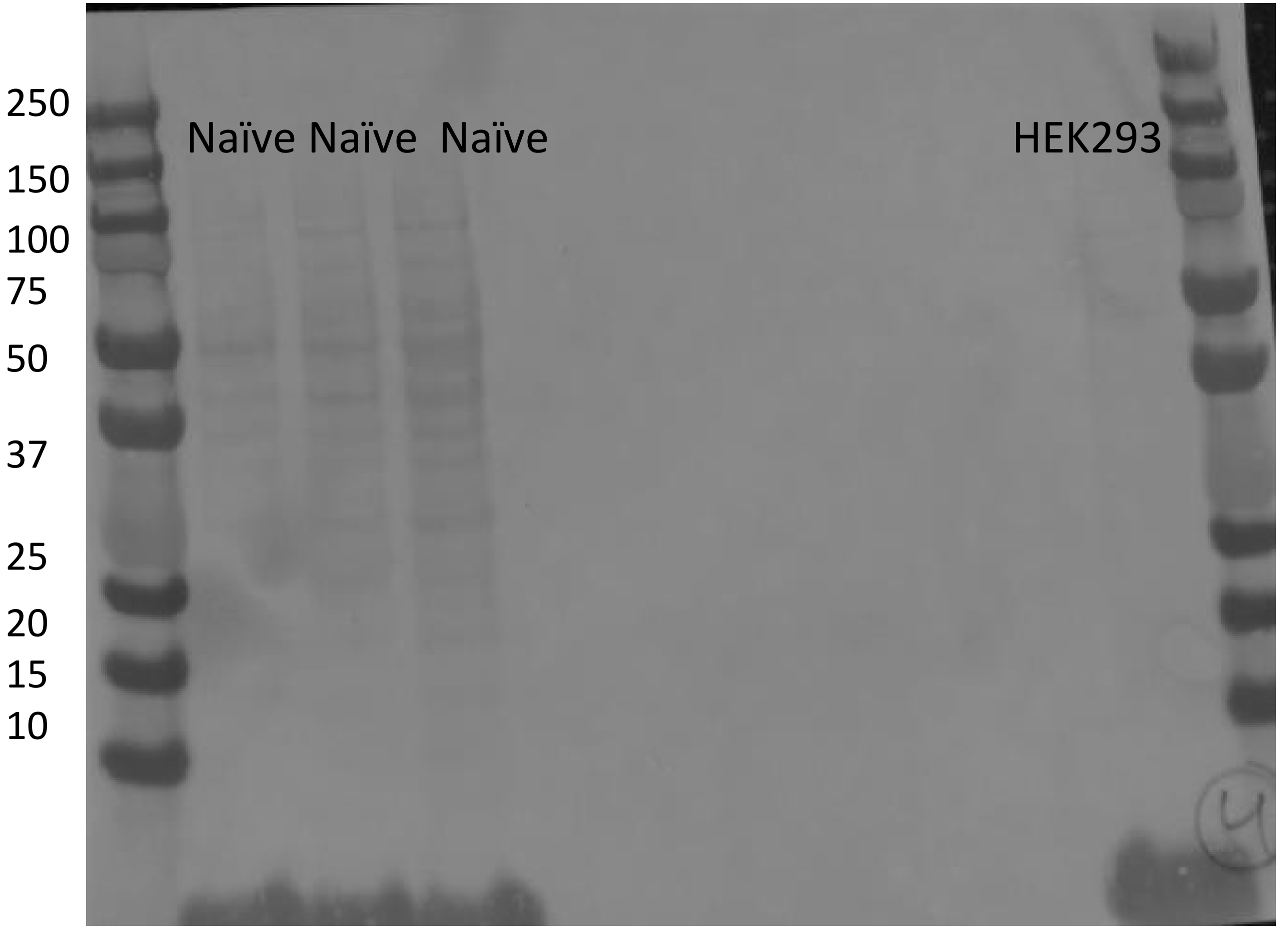

Protein load: SMC, **10 µg**
Ab . Primary . SOD2 ENZO. 1:1500

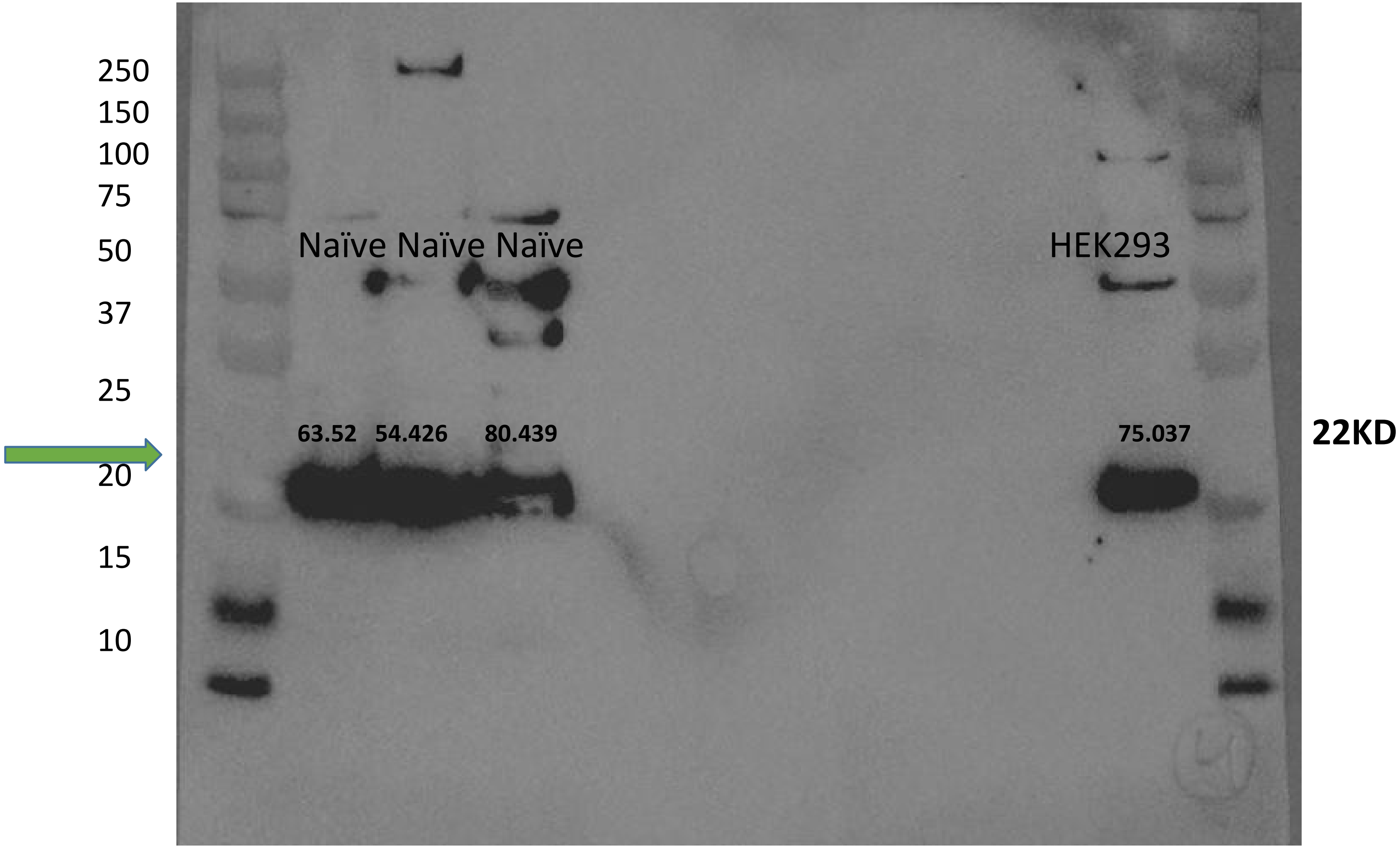

Membrane 5- Ponceau

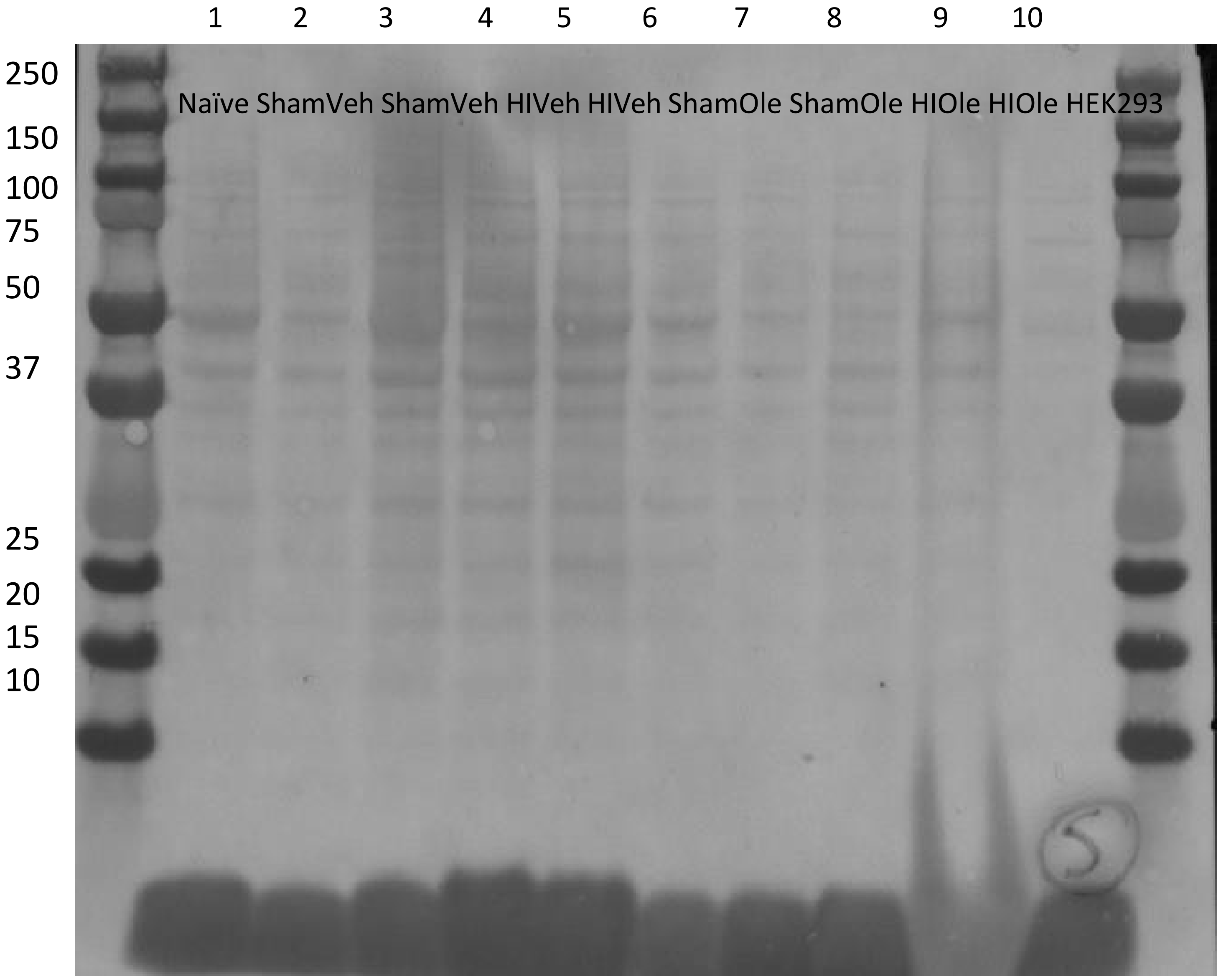

Protein load: WM, **10 µg**
Ab . Primary . SOD2 ENZO. 1:1500

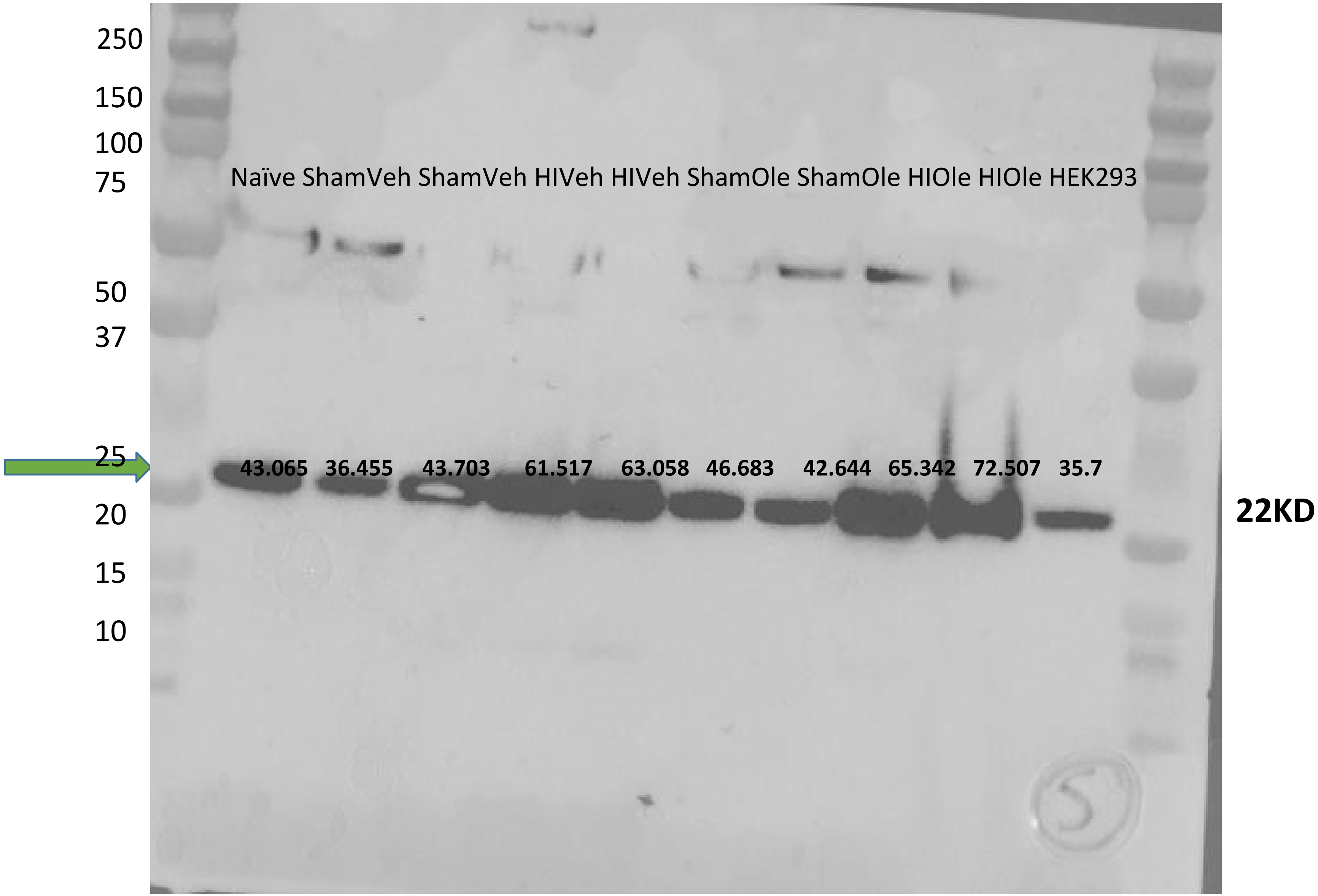

Membrane 6- Ponceau

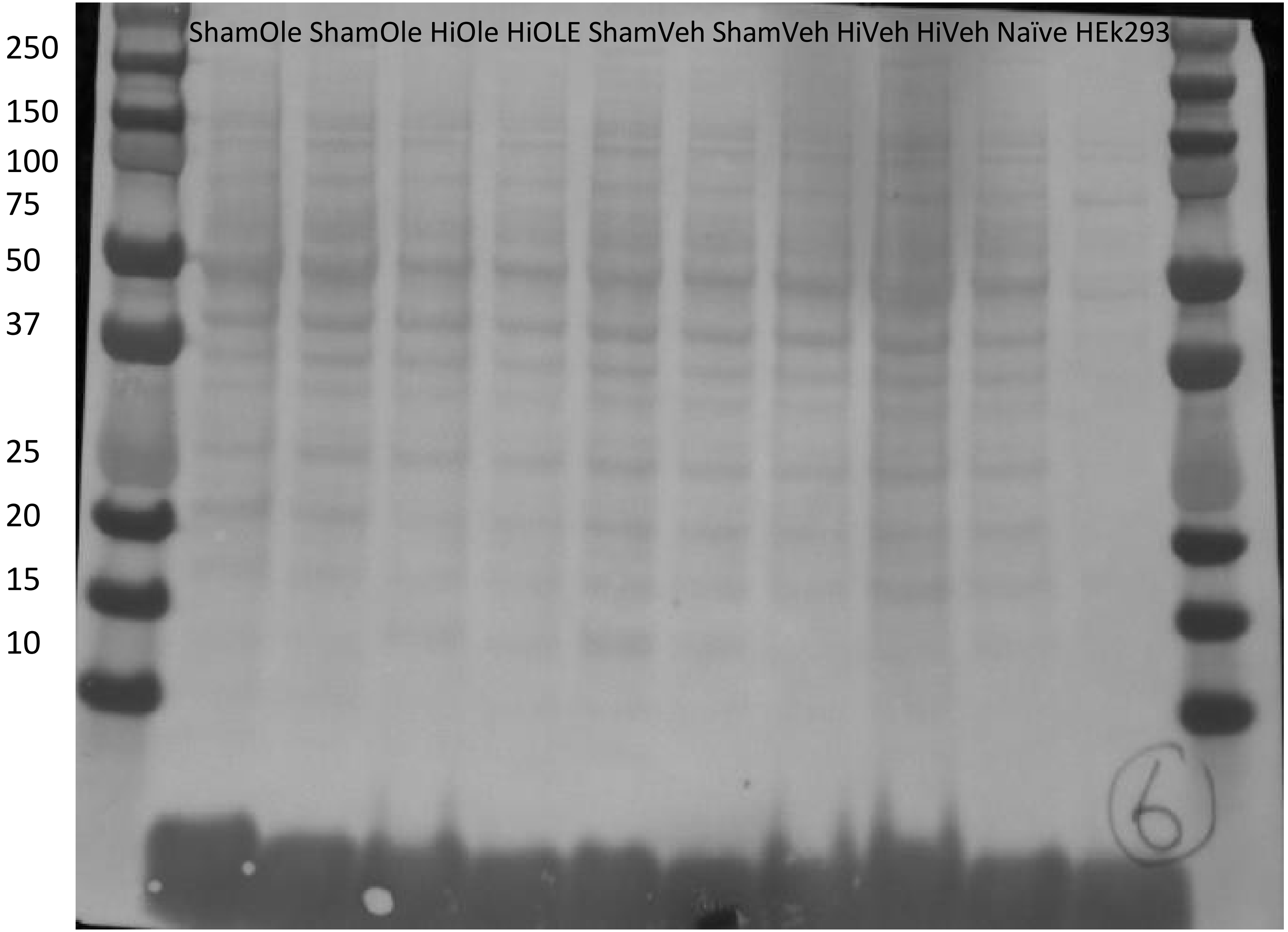

Protein load: WM, **10 µg**
Ab . Primary . SOD2 ENZO. 1:1500

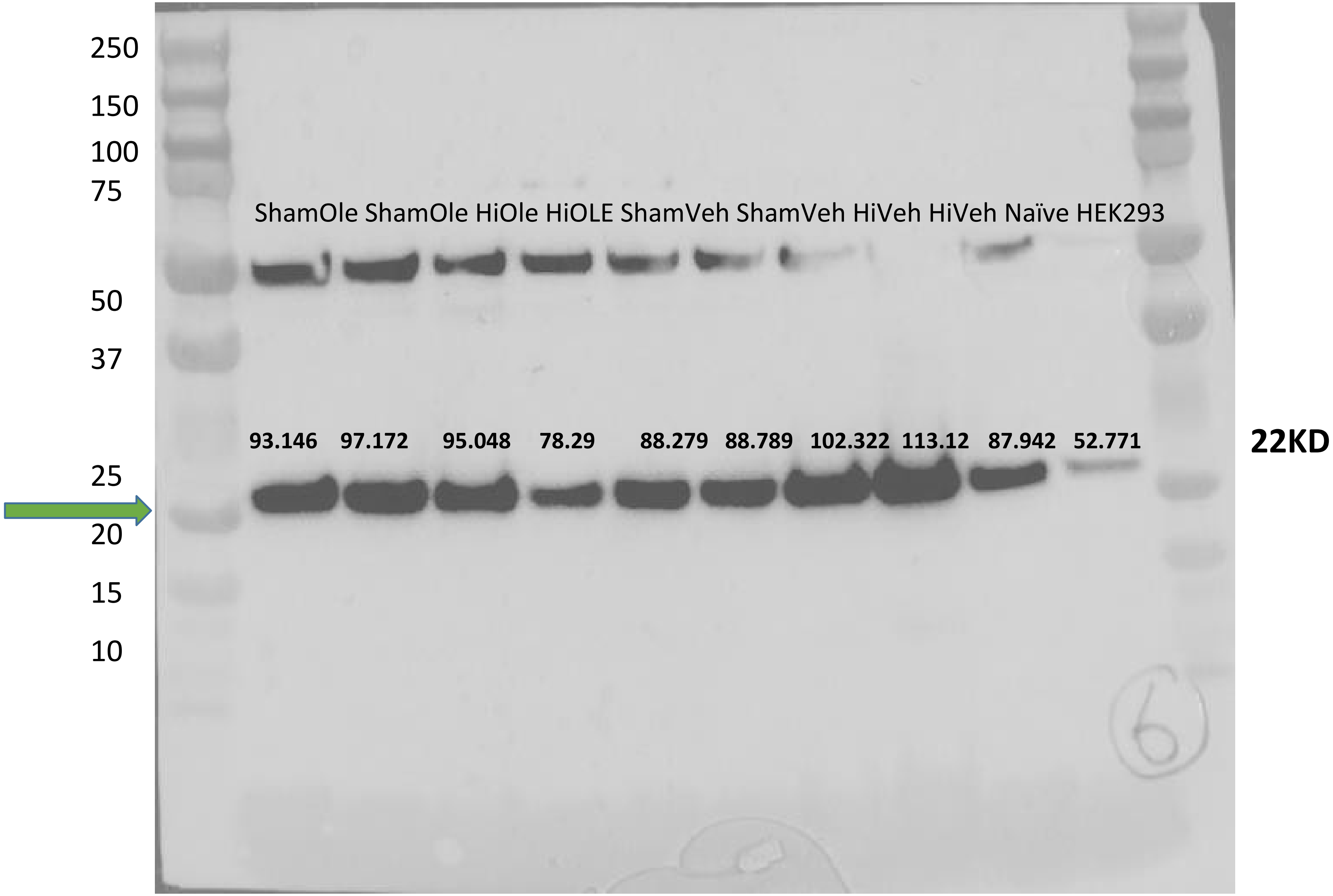

Membrane 7- Ponceau

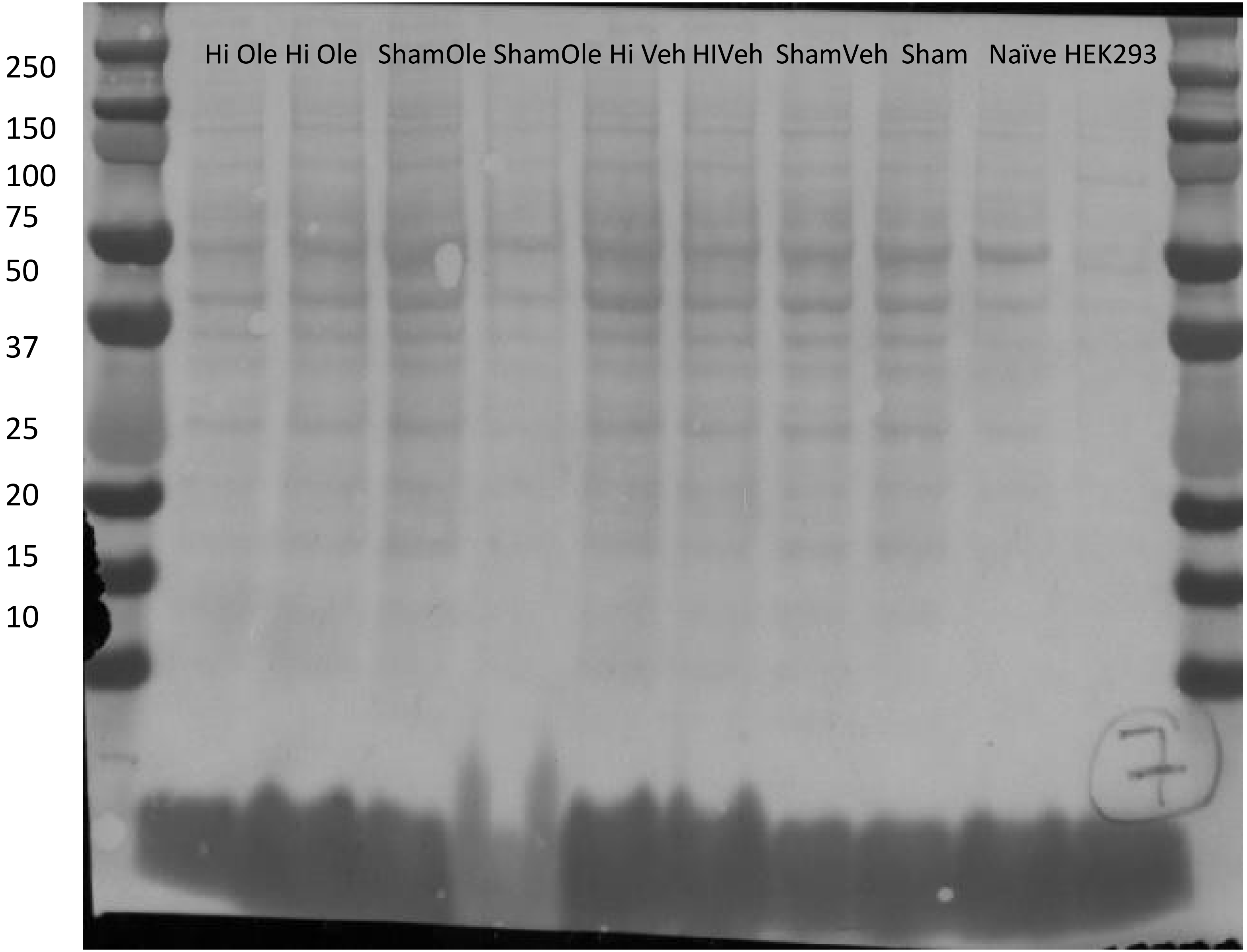

Protein load: WM, **10 µg**
Ab . Primary . SOD2 ENZO. 1:1500

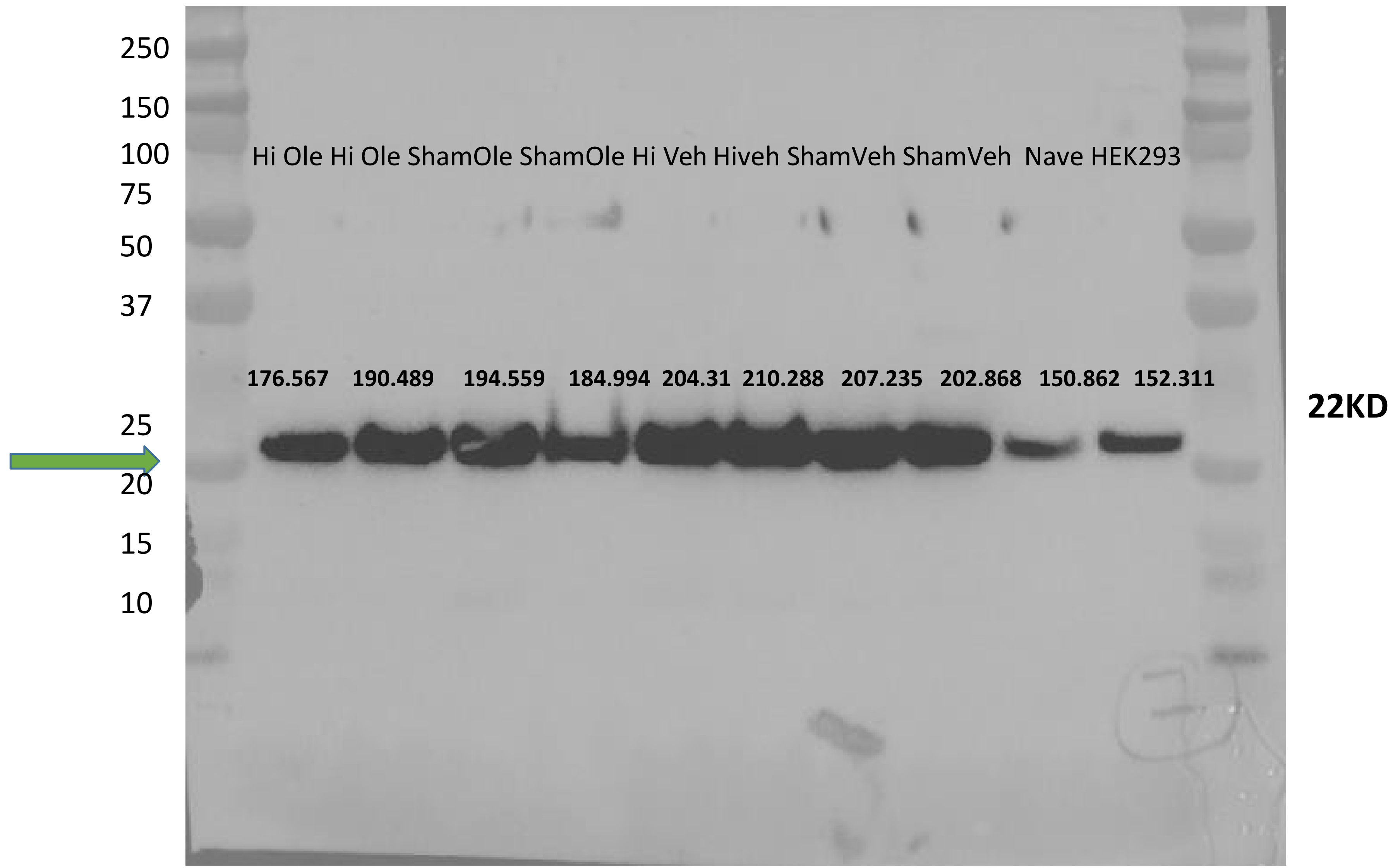

Membrane 8- Ponceau

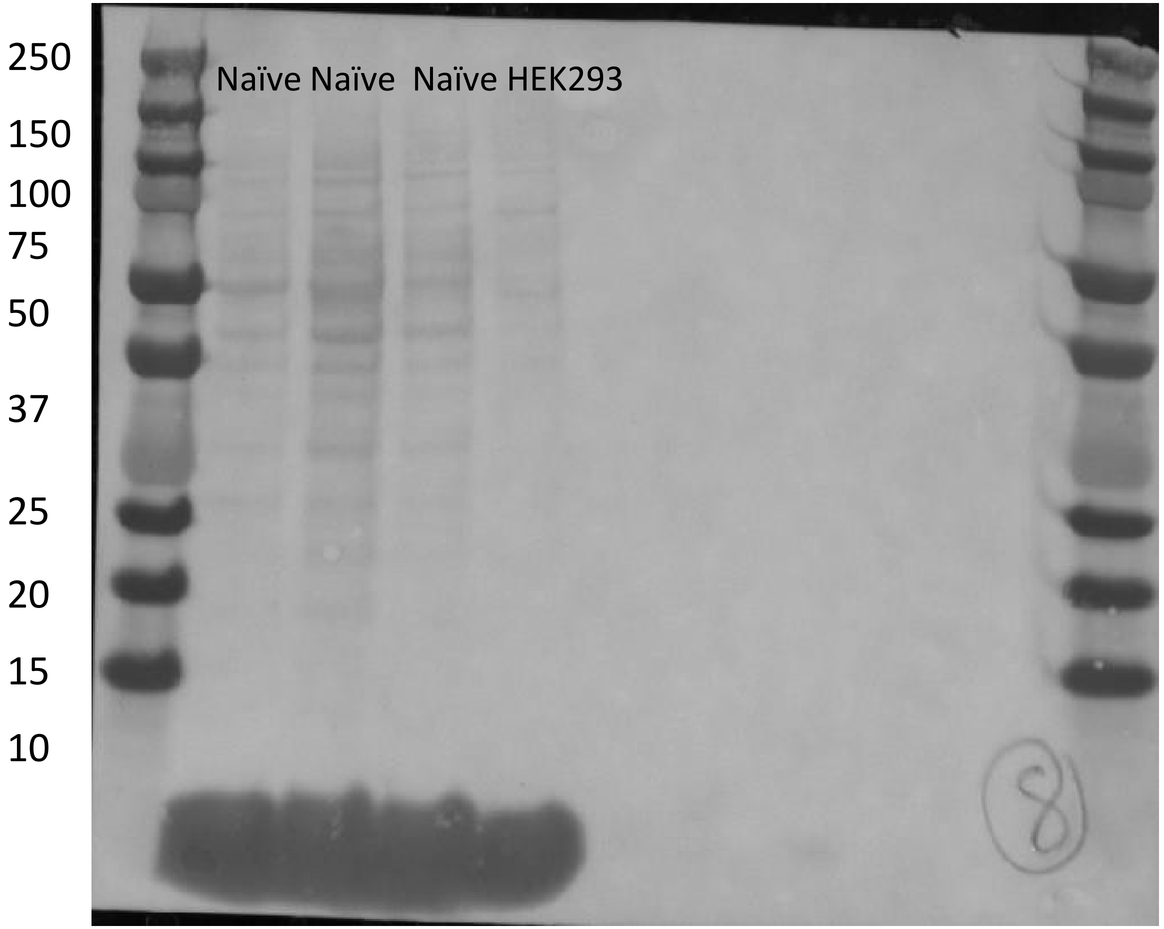

Protein load: WM, **10 µg**
Ab . Primary . SOD2 ENZO. 1:1500

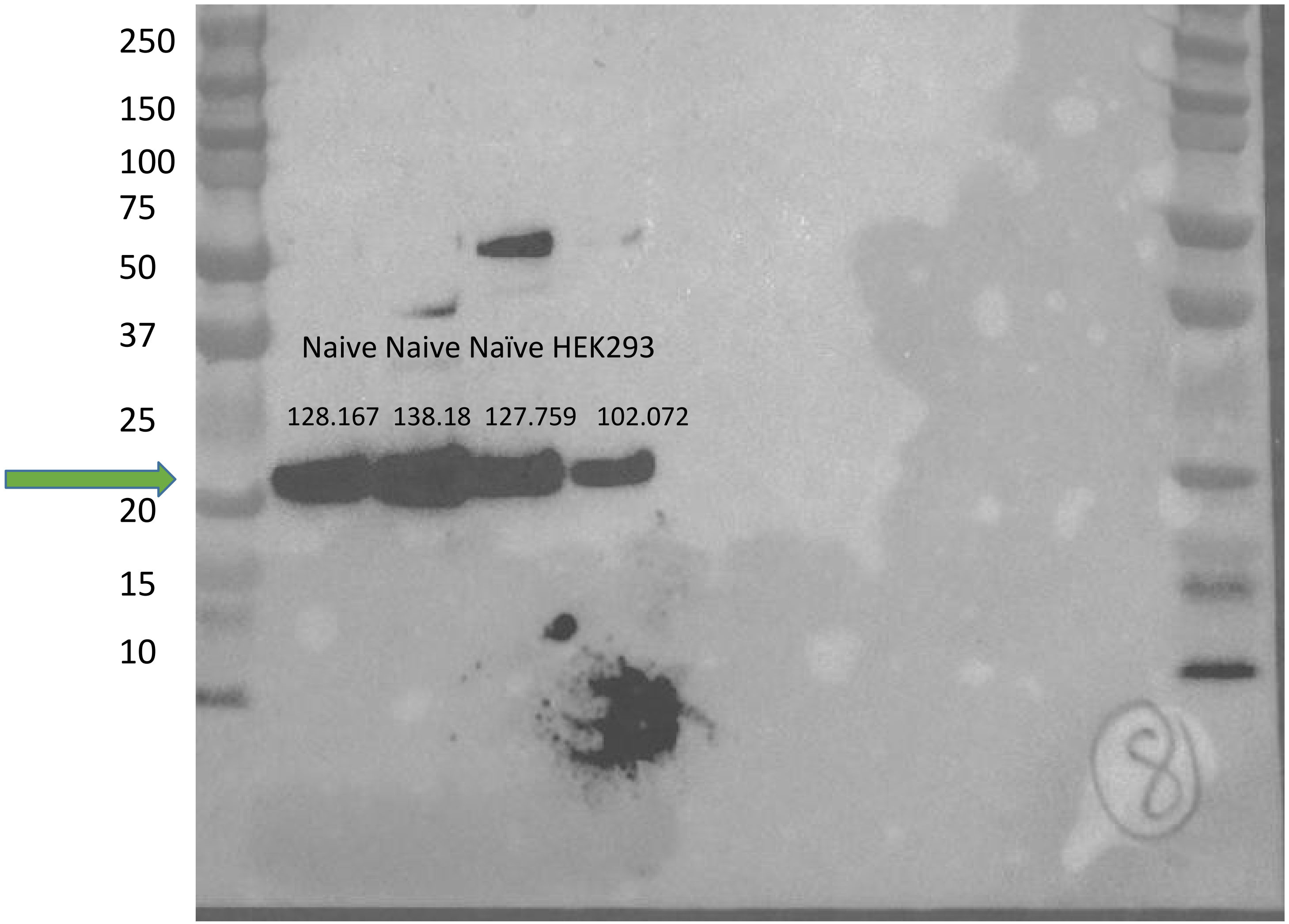

Caspase-7
Full-Length Western blots

Membrane 1- Ponceau

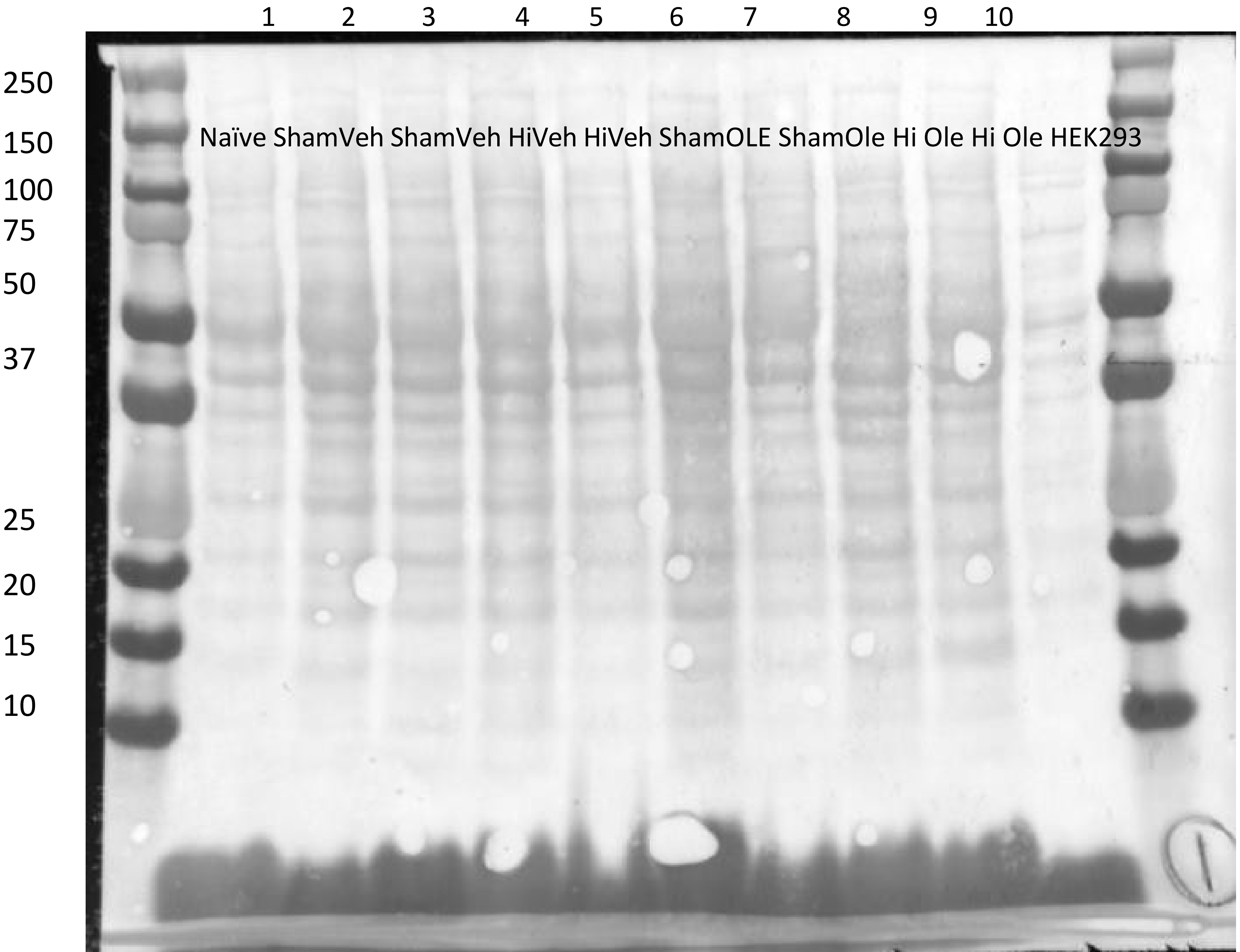

Protein load: SMC, **50 µg**

Ab . Primary . Caspase7 cell signaling . 1:1000

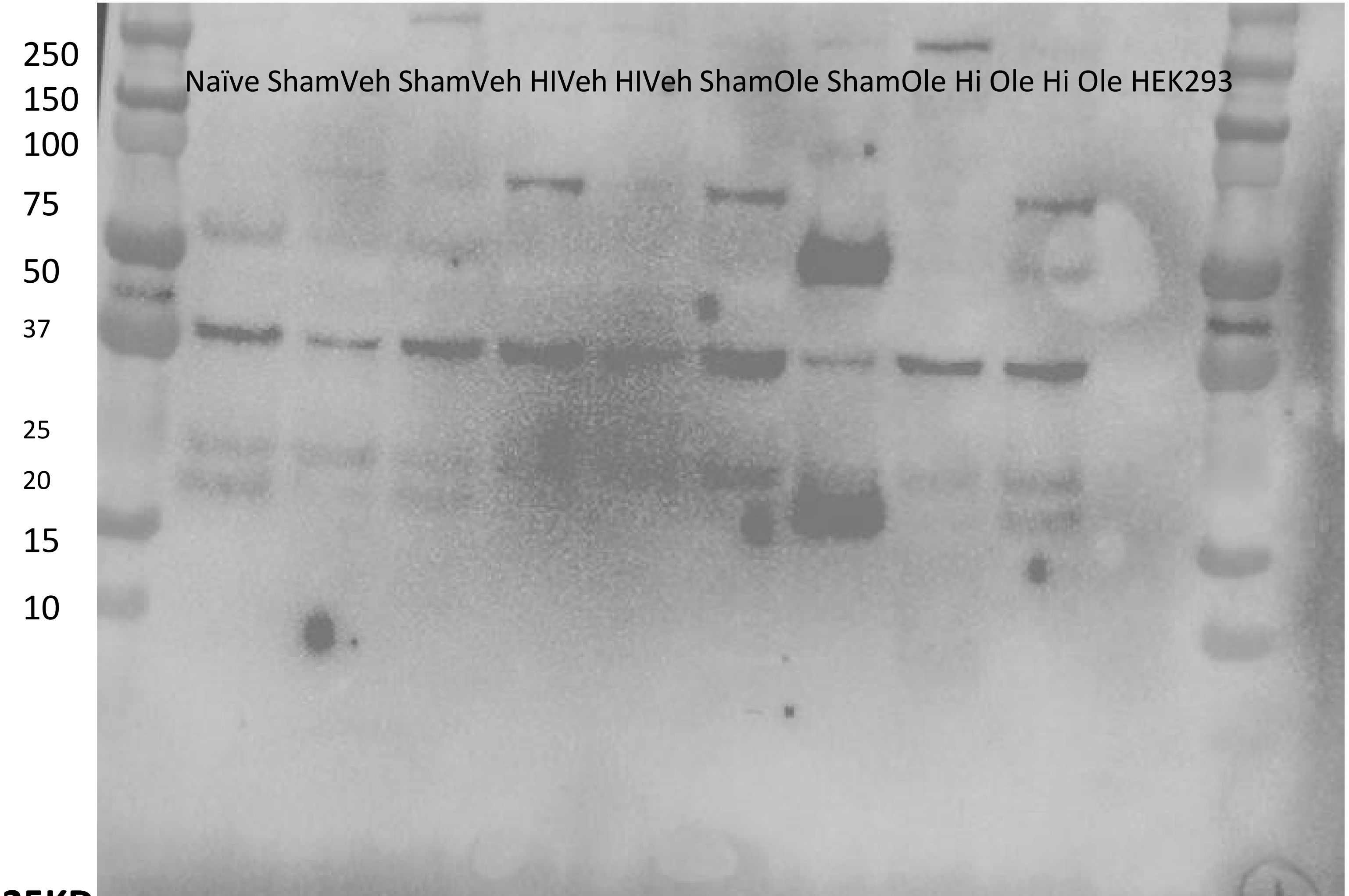

**25KD**	**119.843**	**117.137**	**124.734**	**152.836**	**160.267**	**157.591**	**147.6**	**119.617**	**121.372**	**113.358**
**18KD**	**108.716**	**113.338**	**124.085**	**143.712**	**146.275**	**148.254**	**155.147**	**117.618**	**124.965**	**113.432**

Membrane 2- Ponceau

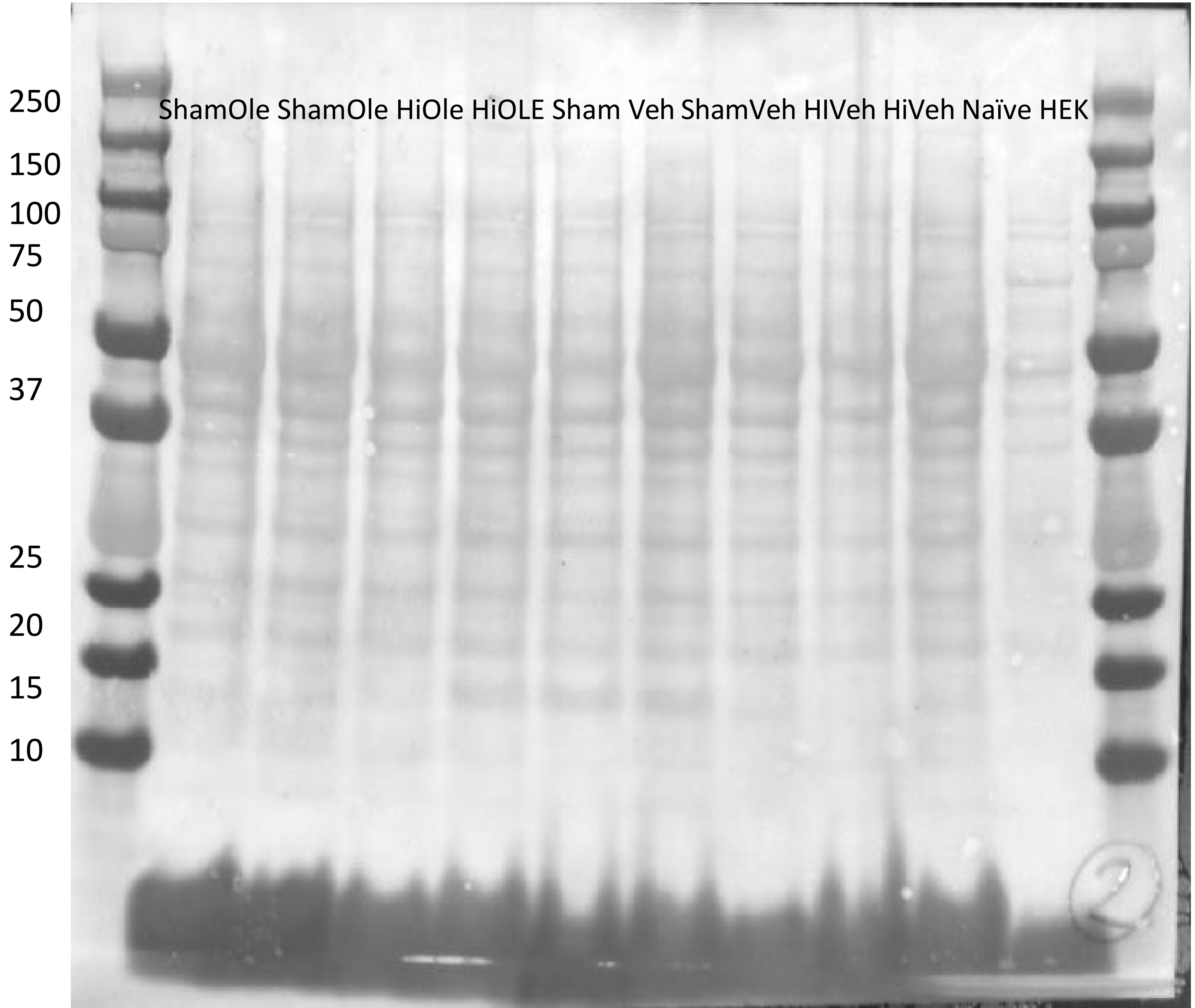

Protein load: SMC, **50 µg**

Ab . Primary . Caspase7 cell signaling . 1:1000

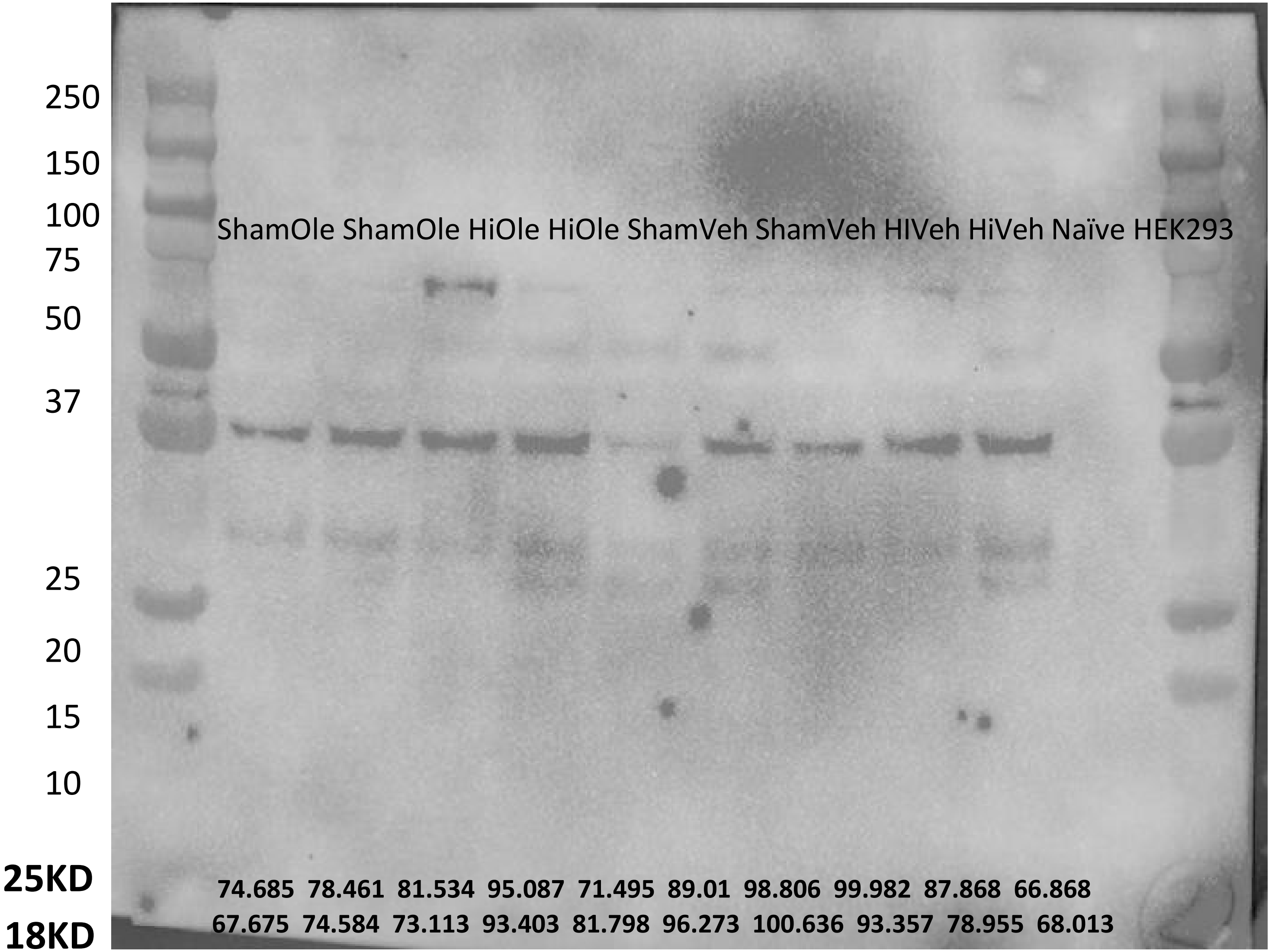

Membrane 3- Ponceau

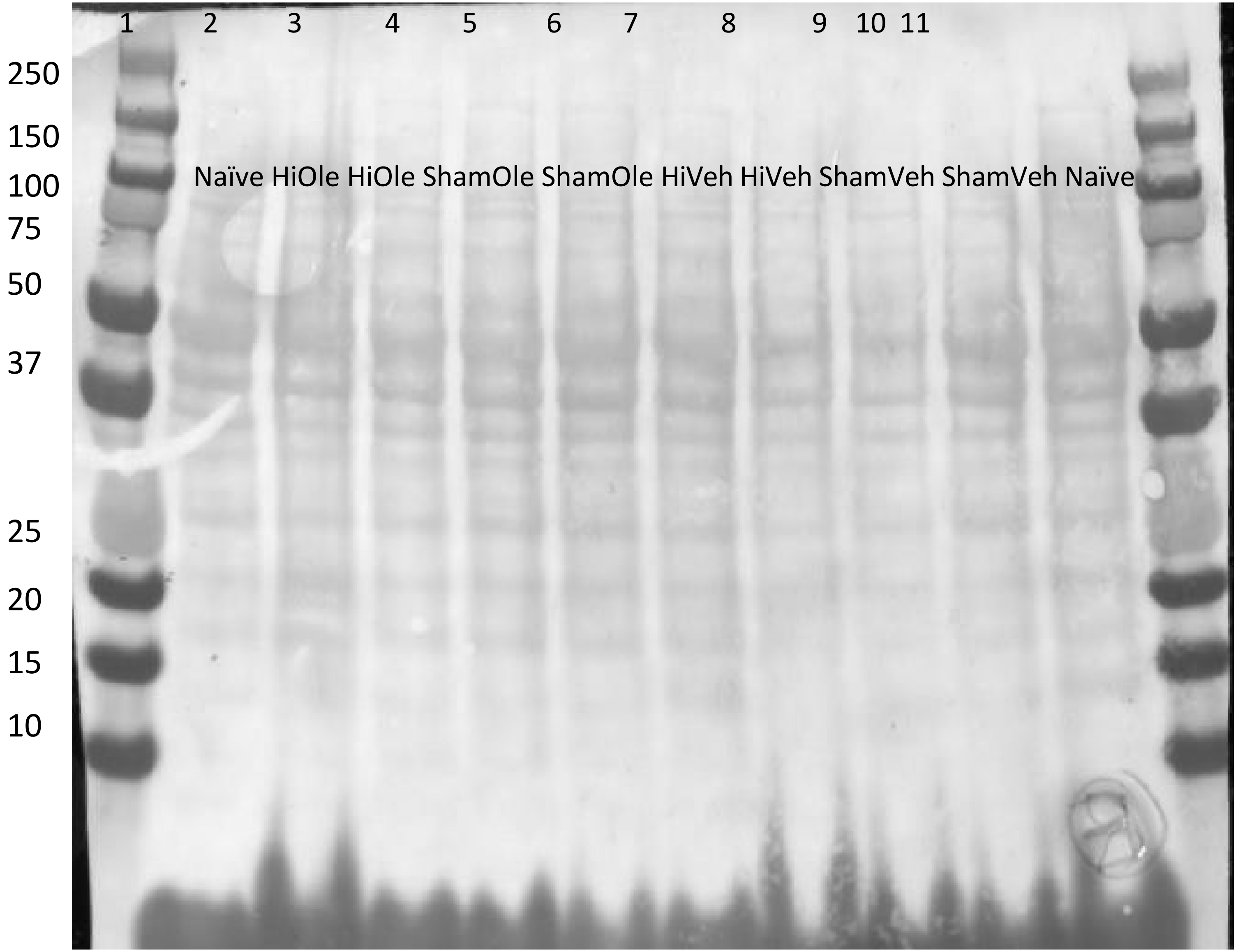

Protein load: SMC, **50 µg**

Ab . Primary . Caspase7 cell signaling . 1:1000

250
150
100
75
50
37
25
20
15
10

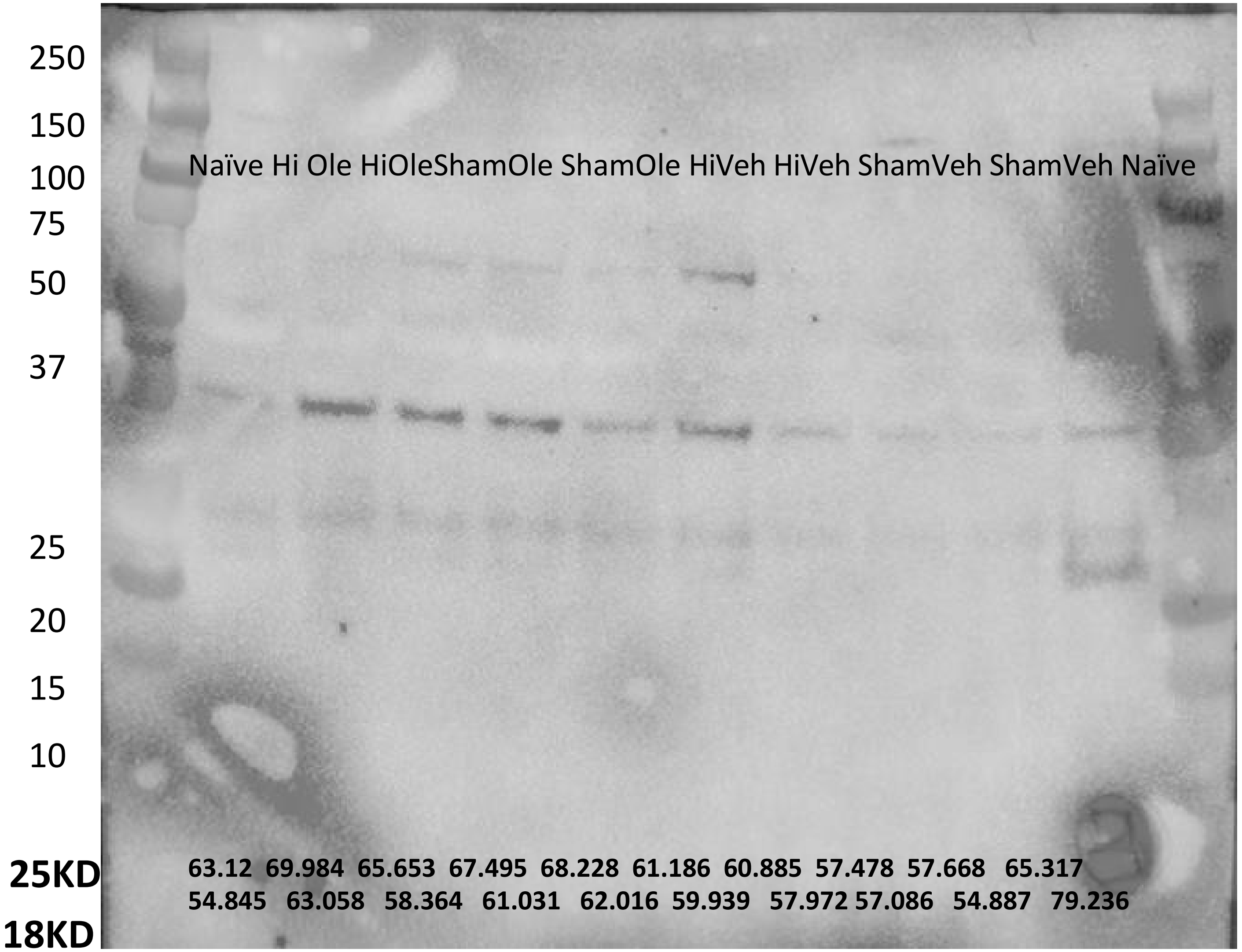

25KD
18KD

Membrane 4- Ponceau

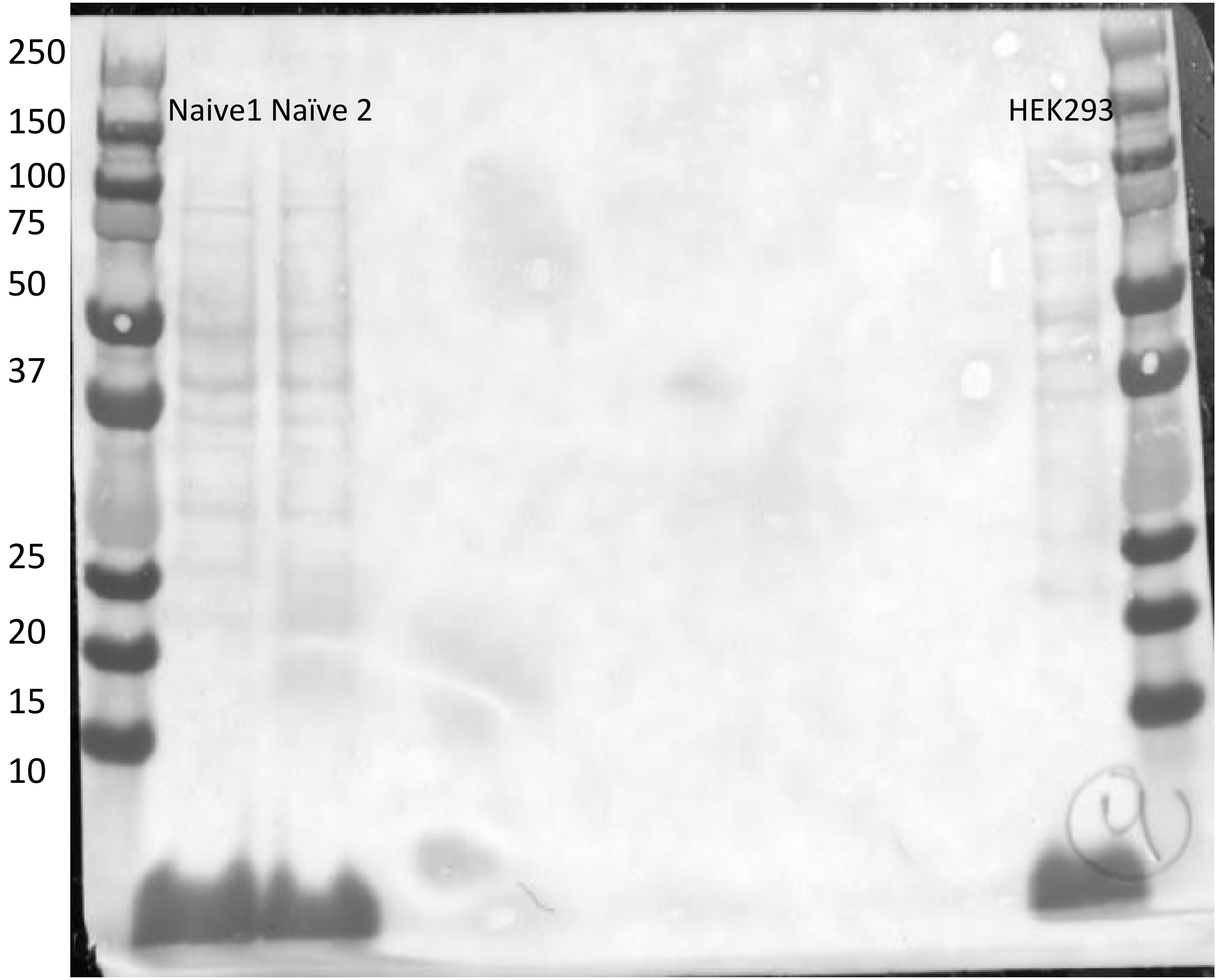

Protein load: SMC, **50 µg**

Ab . Primary . Caspase7 cell signaling . 1:1000

250
150
100
75
50
37
25
20
15
10
25KD
18KD

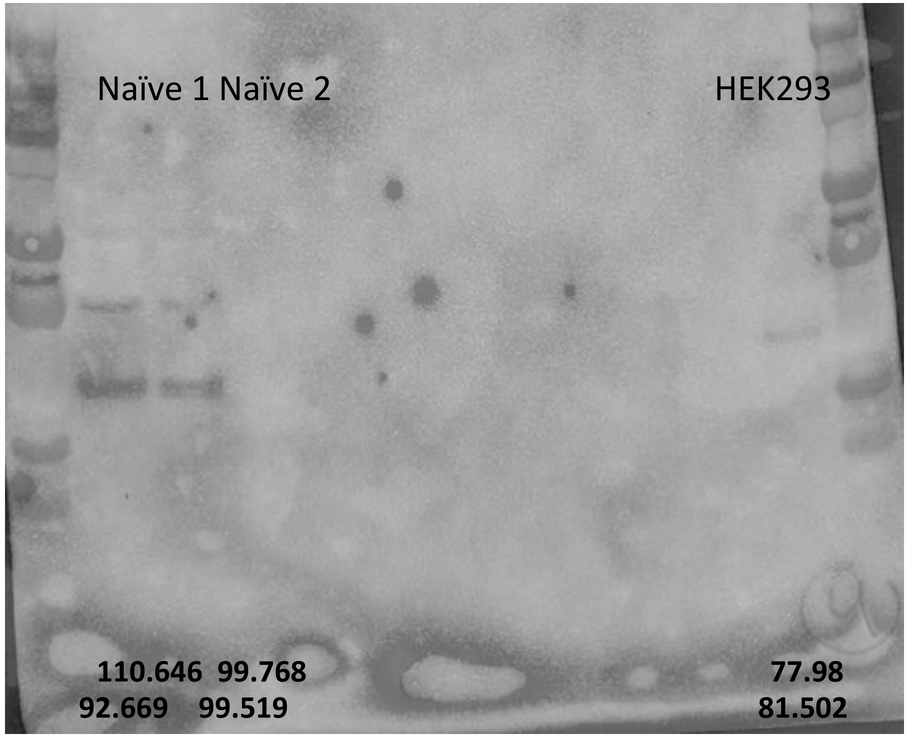

Ubiquitin
Full-Length Western blots

Membrane 1- Ponceau

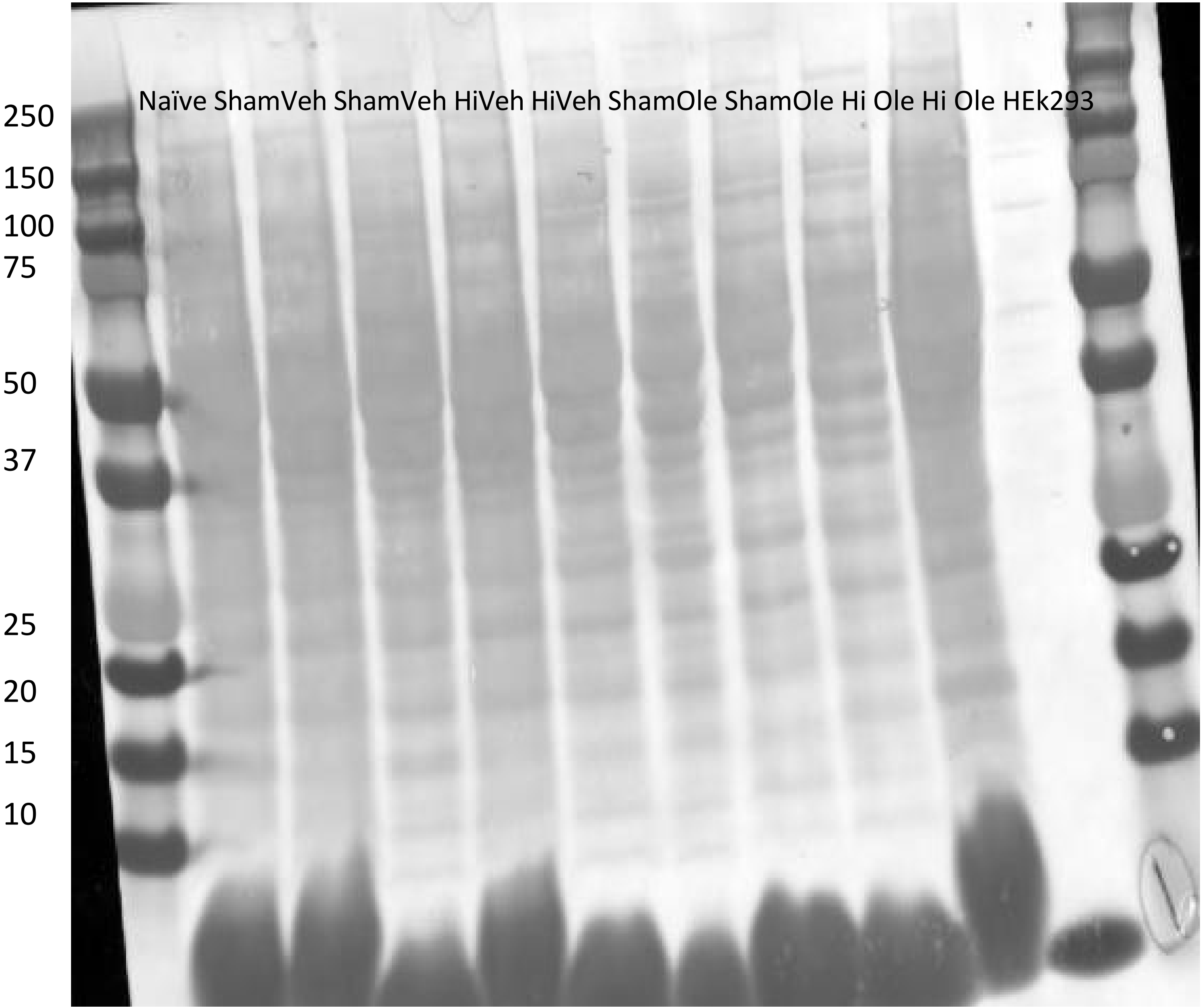

Protein load: SMC, **50 μg**
Ab . Primary . Ubiquitin Abcam. 1:3000

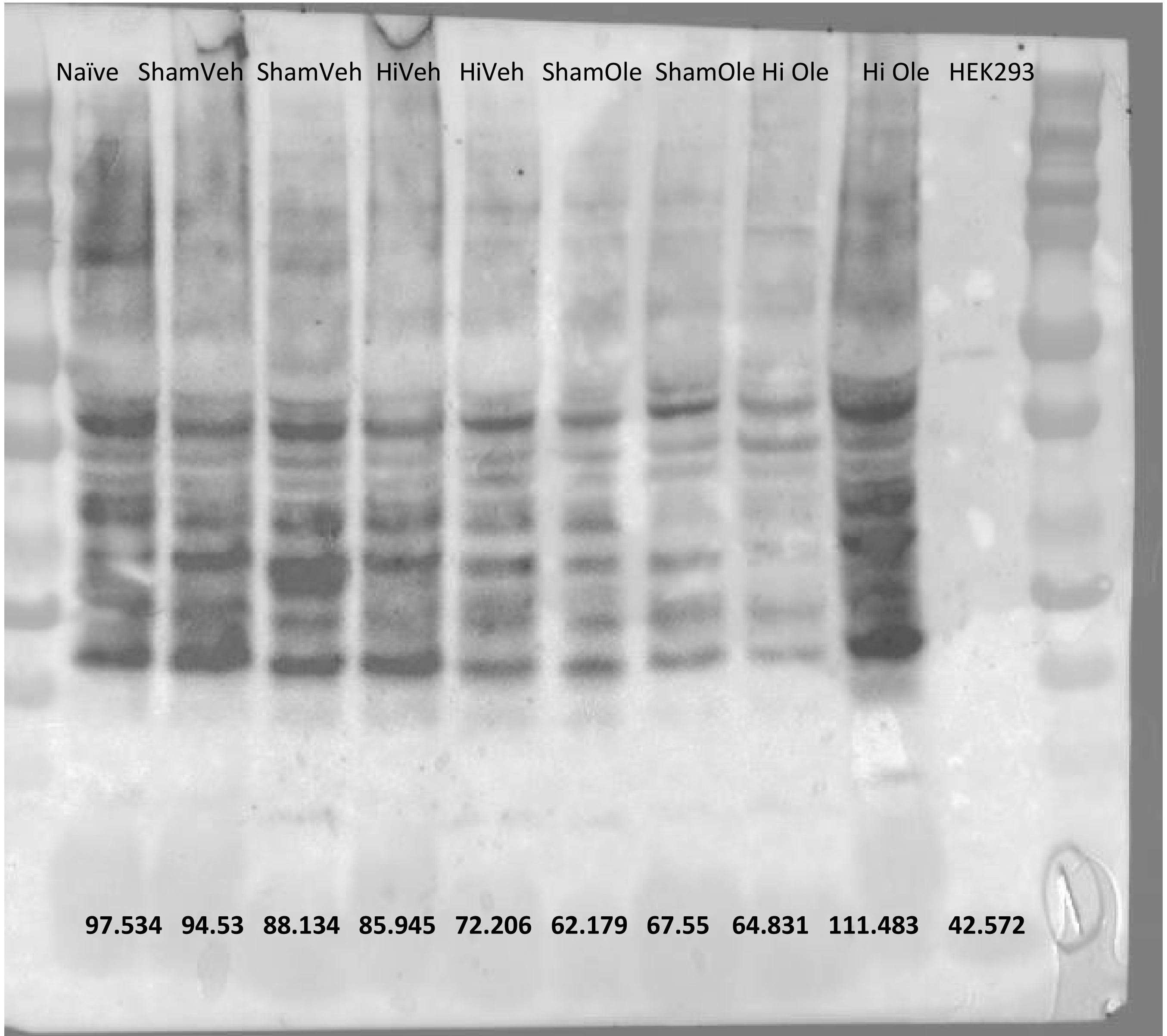

**Total Density (10-250)**	**97.534**	**94.53**	**88.134**	**85.945**	**72.206**	**62.179**	**67.55**	**64.831**	**111.483**	**42.572**

Membrane 2- Ponceau

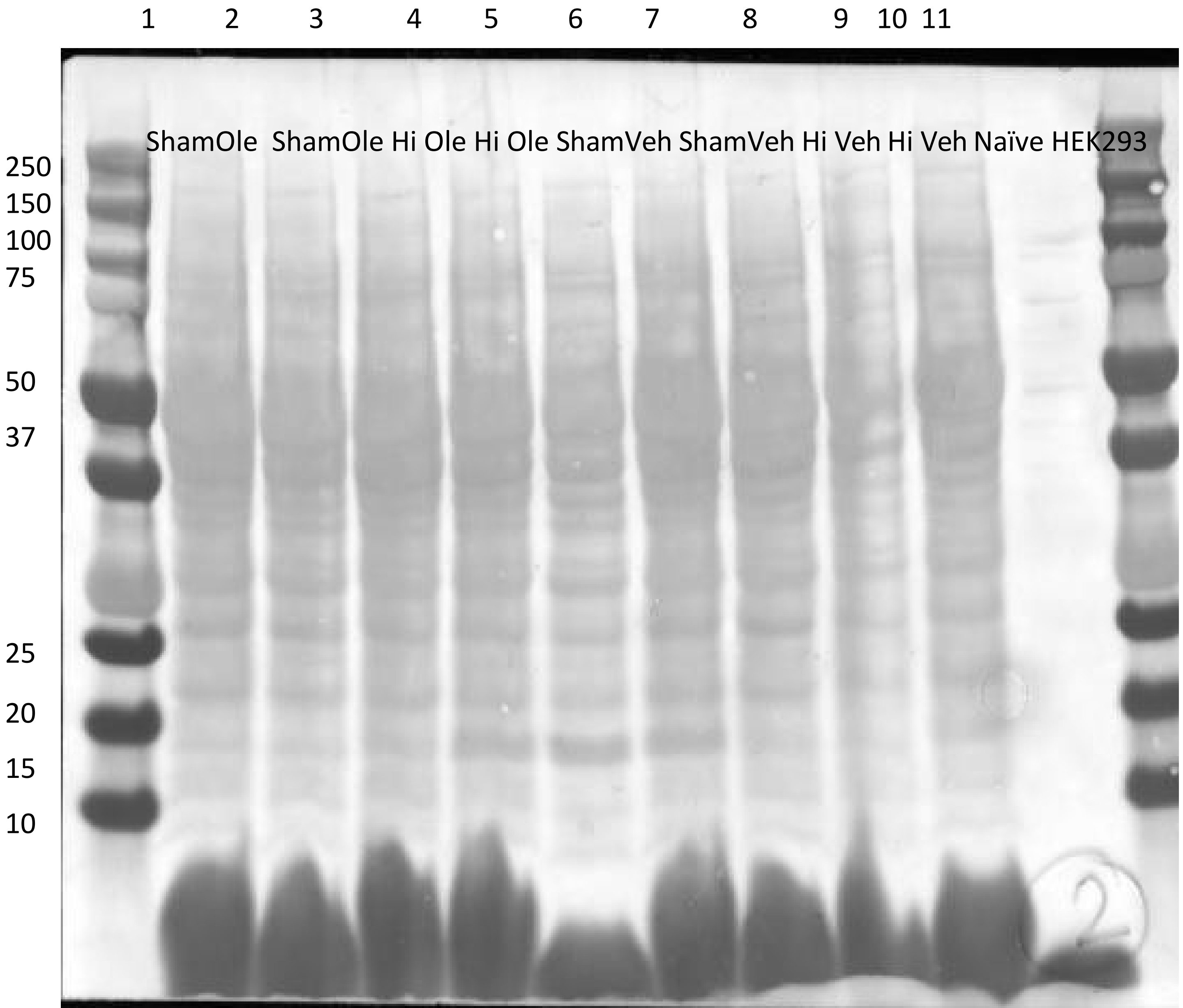

Protein load: SMC, **50 µg**

Ab . Primary . Ubiquitin Abcam. 1:3000

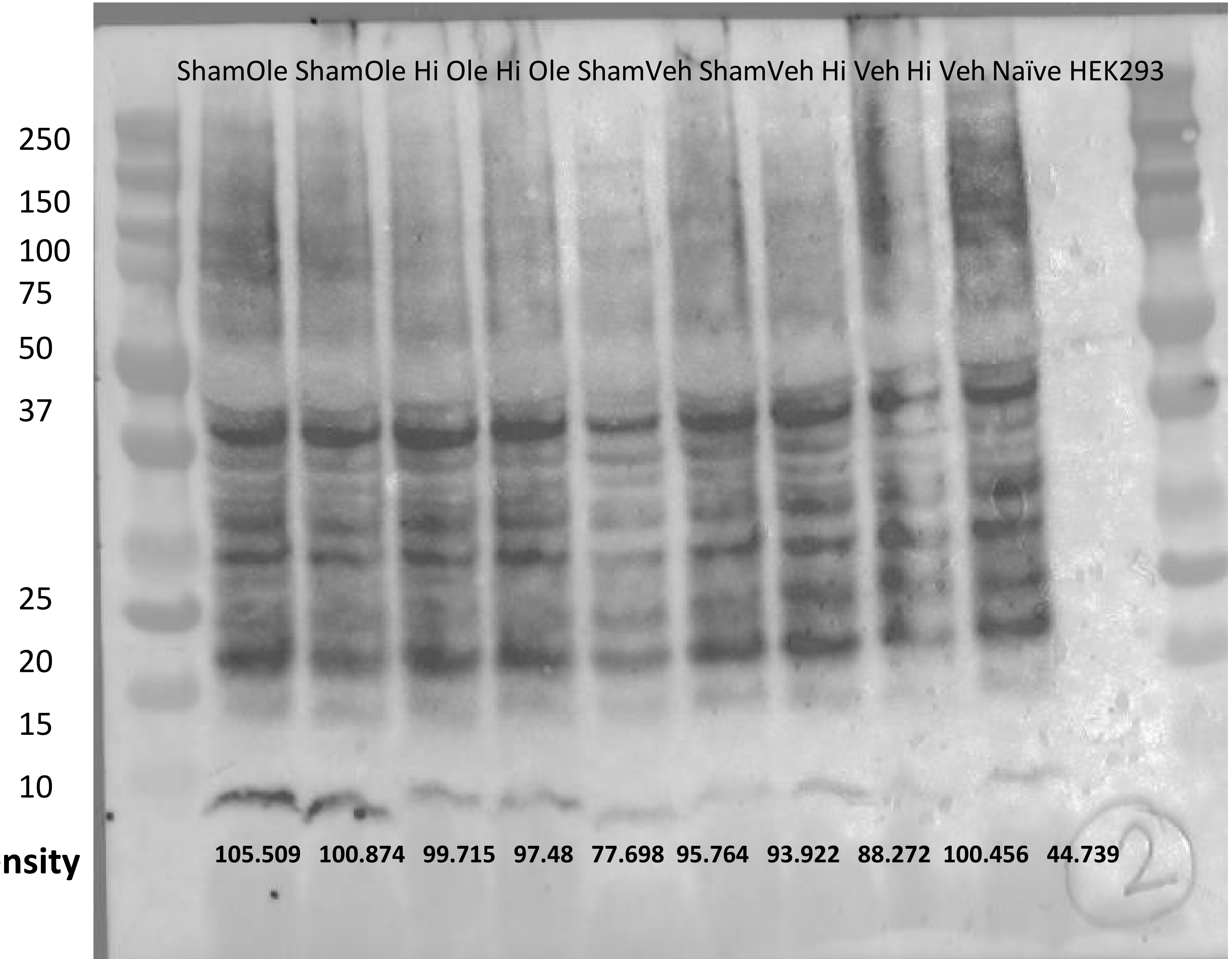

Total Density (10-250)

Membrane 3- Ponceau

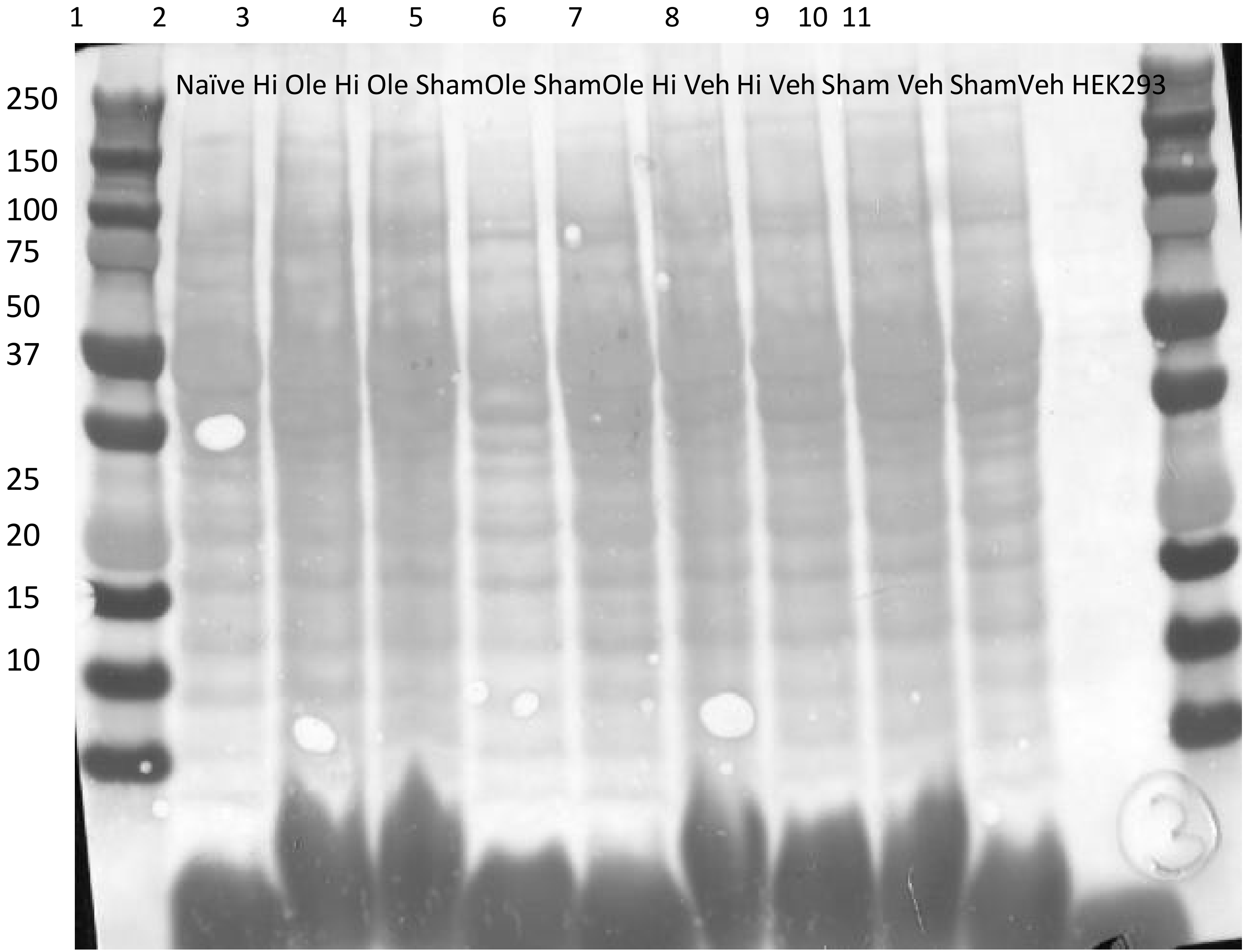

Protein load: SMC, **50 µg**
Ab . Primary . Ubiquitin Abcam. 1:3000

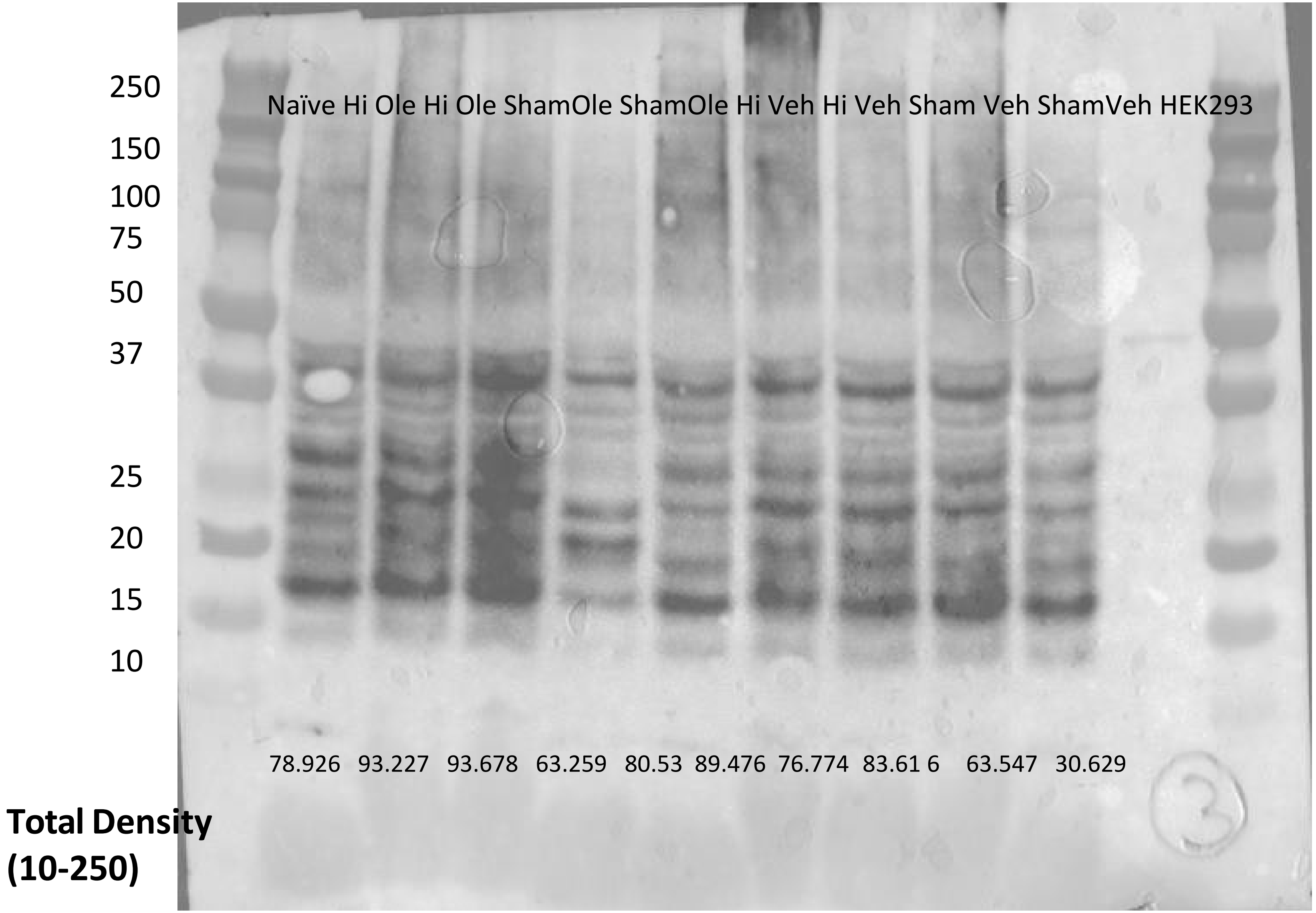

Membrane 4- Ponceau

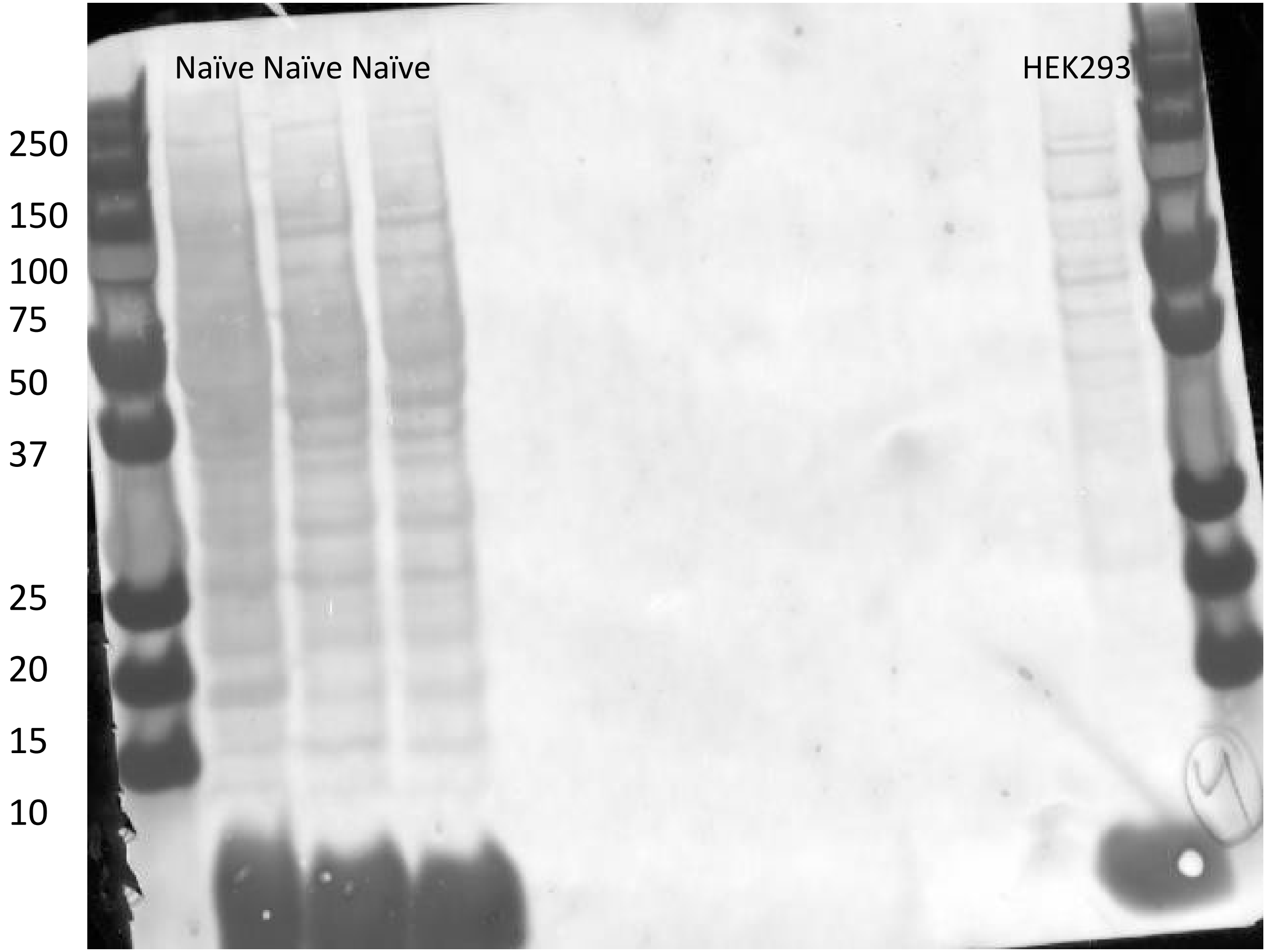

Protein load: SMC, **50 µg**
Ab . Primary . Ubiquitin Abcam. 1:3000

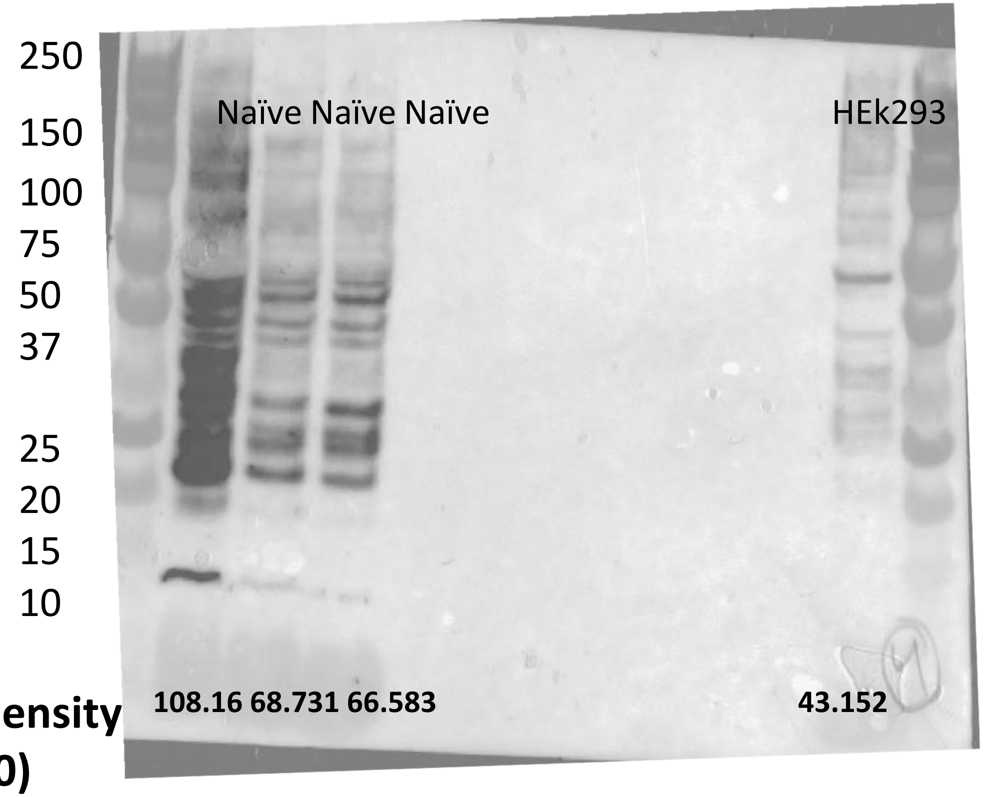

Total Density (10-250)

Full-Length Western blots for AAV Experiments

AAV Pigs
PSMA3

Protein load: SMC, **5 0µg**
Ab . Primary . PSMA3. 1:1000

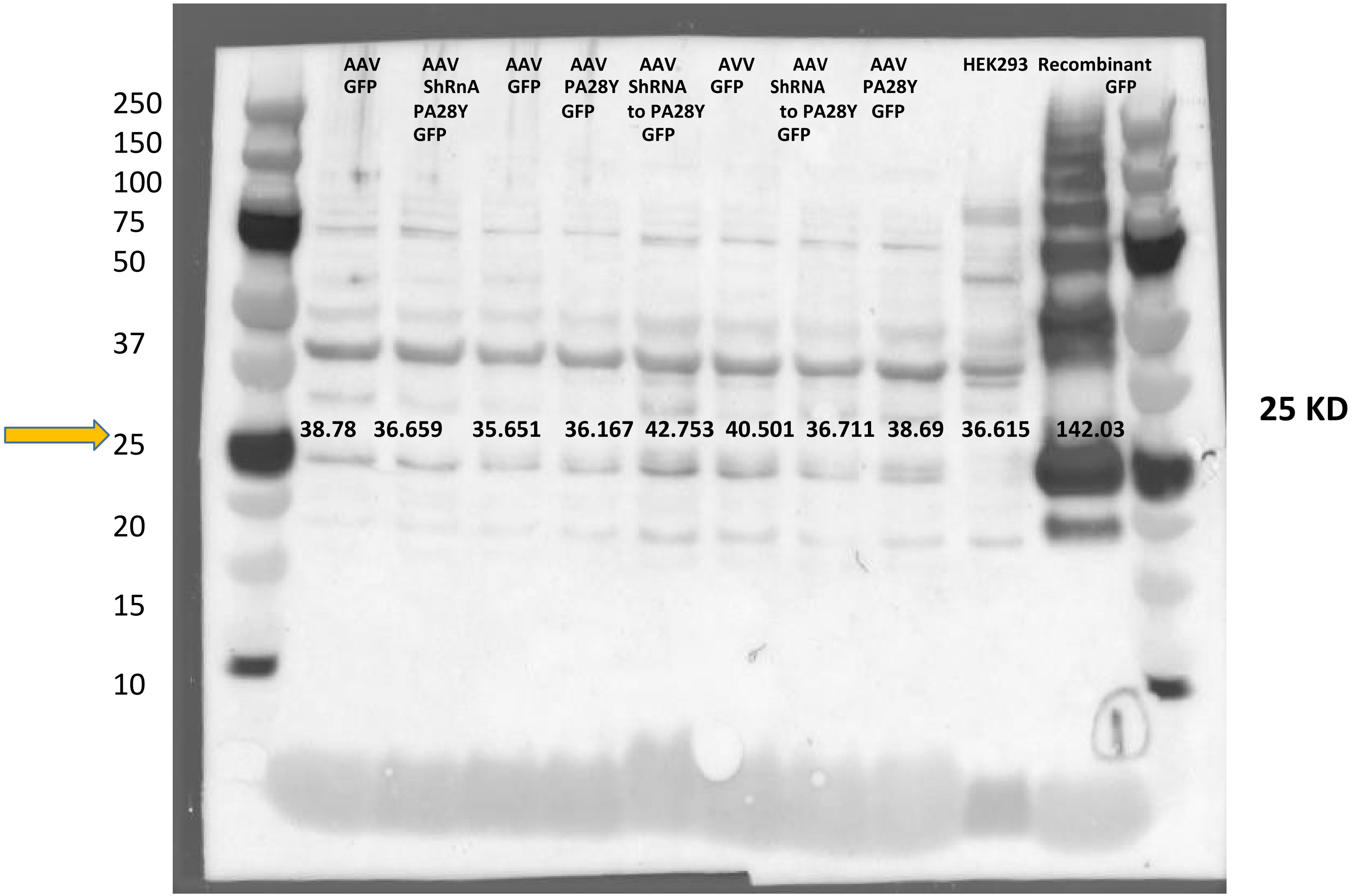

Protein load: SMC, **50 µg**
Ab . Primary . PSMA3. 1:1000

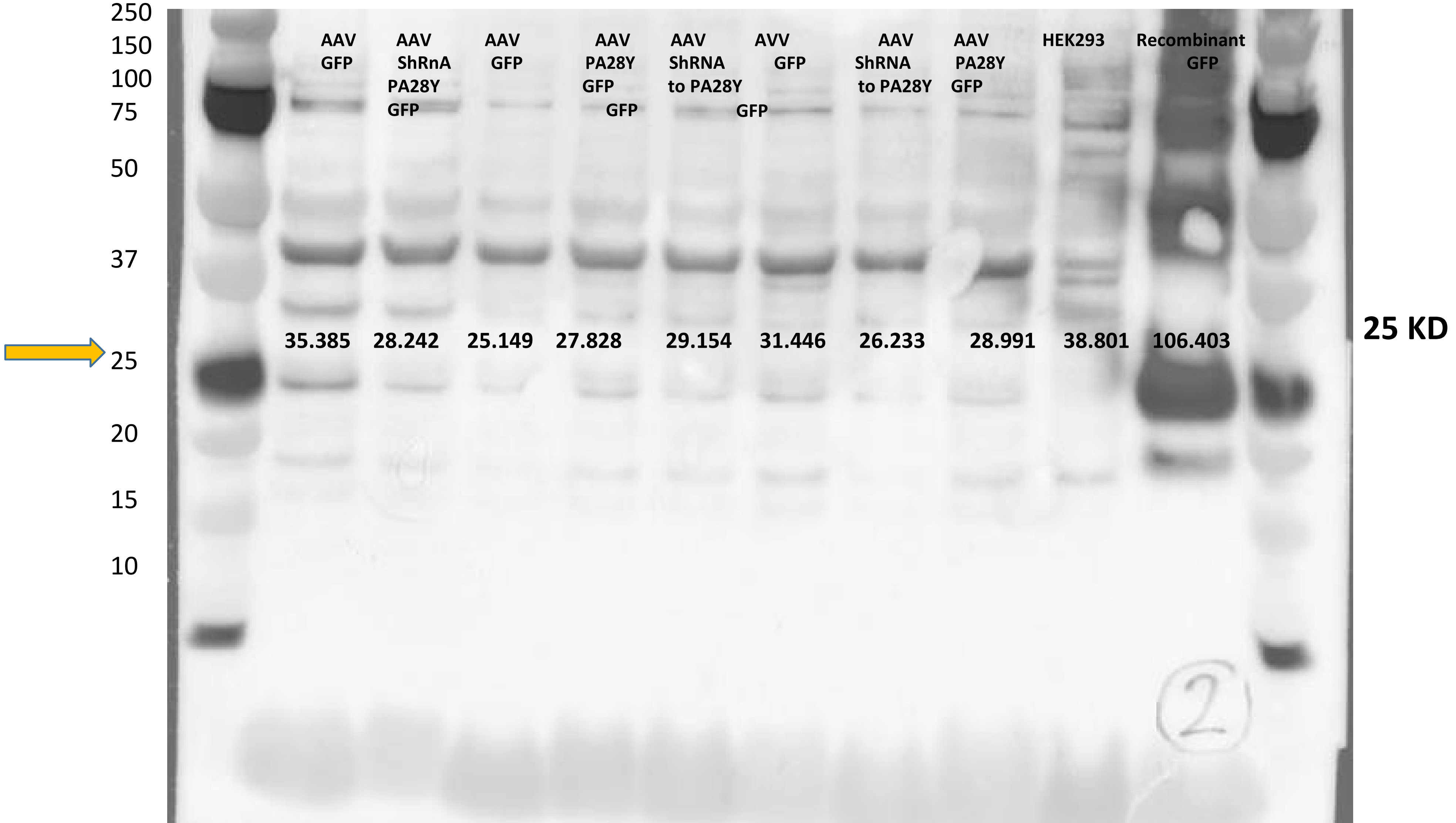

Protein load: WM, **50 µg**

Ab . Primary PSMA3. 1:1000

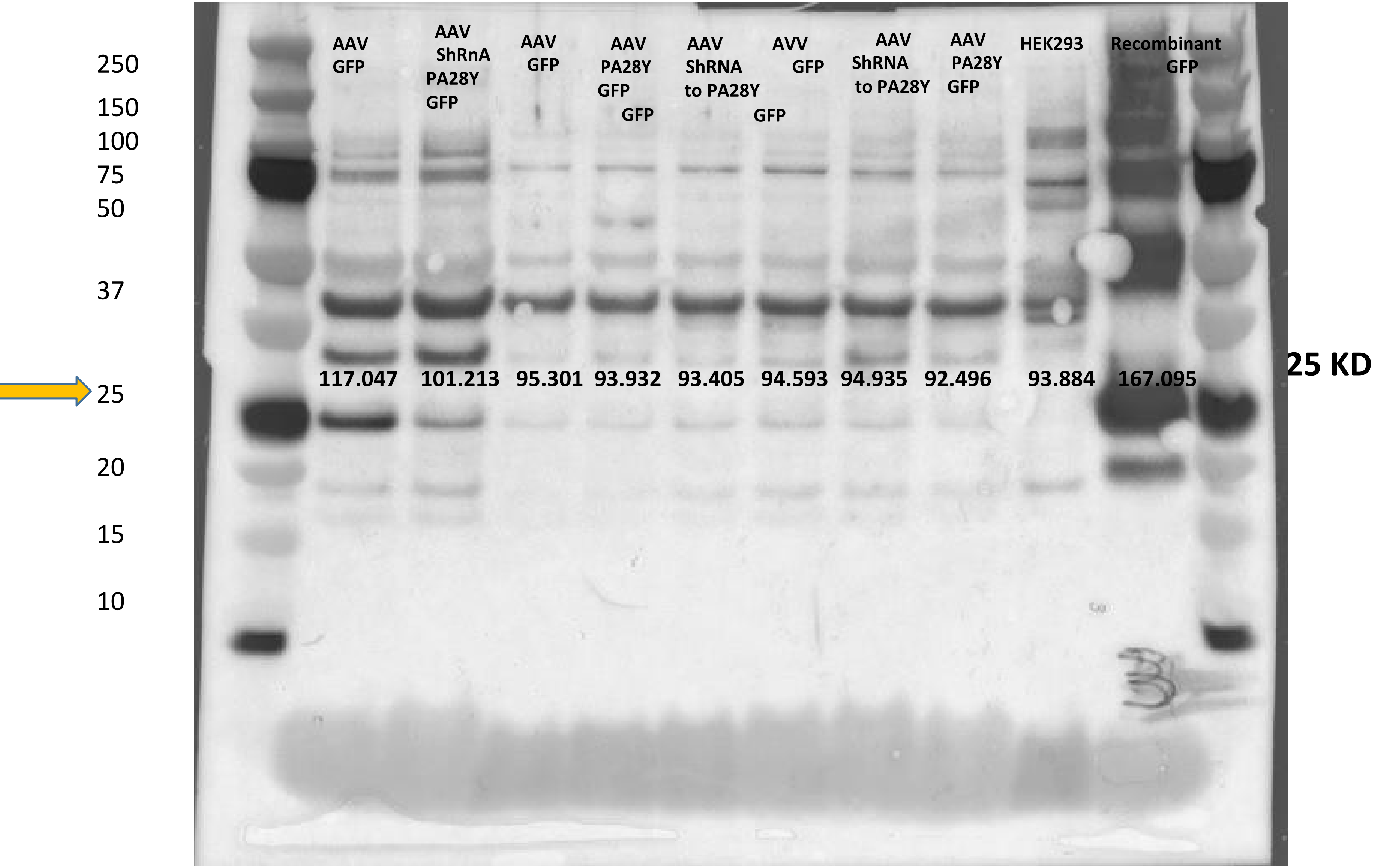

Protein load: WM, **50 µg**
Ab . Primary . PSMA3. 1:1000

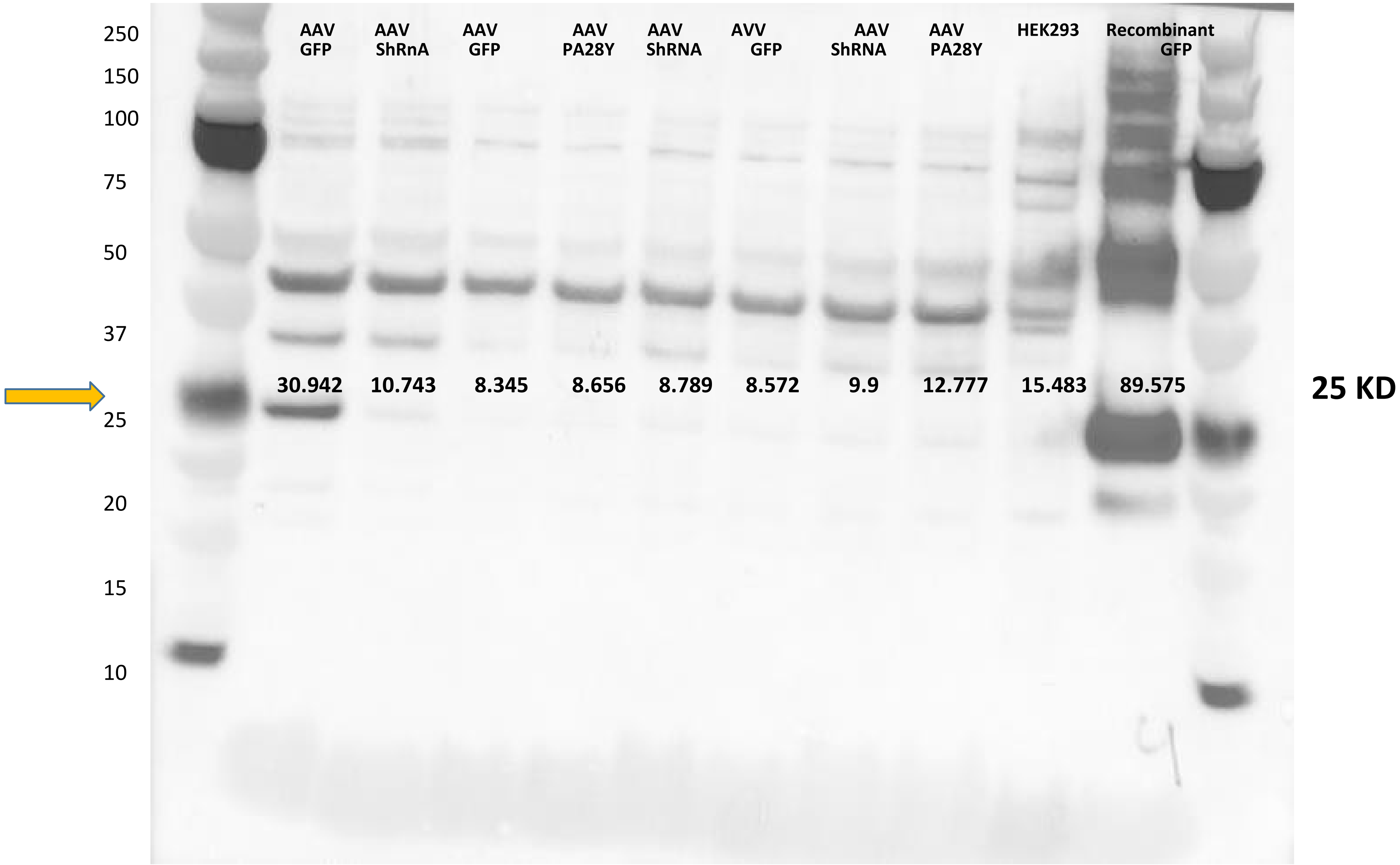

AAV Pigs
GFP

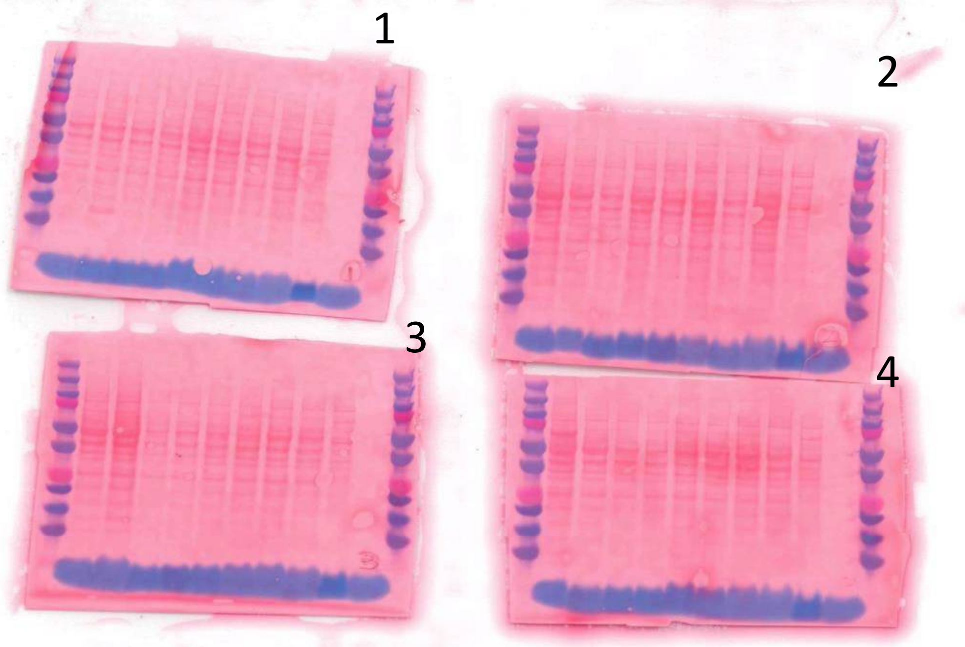

Ponceau membranes correspond to the next 4 WBs

Protein load: SMC, **50 µg**
Ab . Primary . GFP Bovison . 1:1500

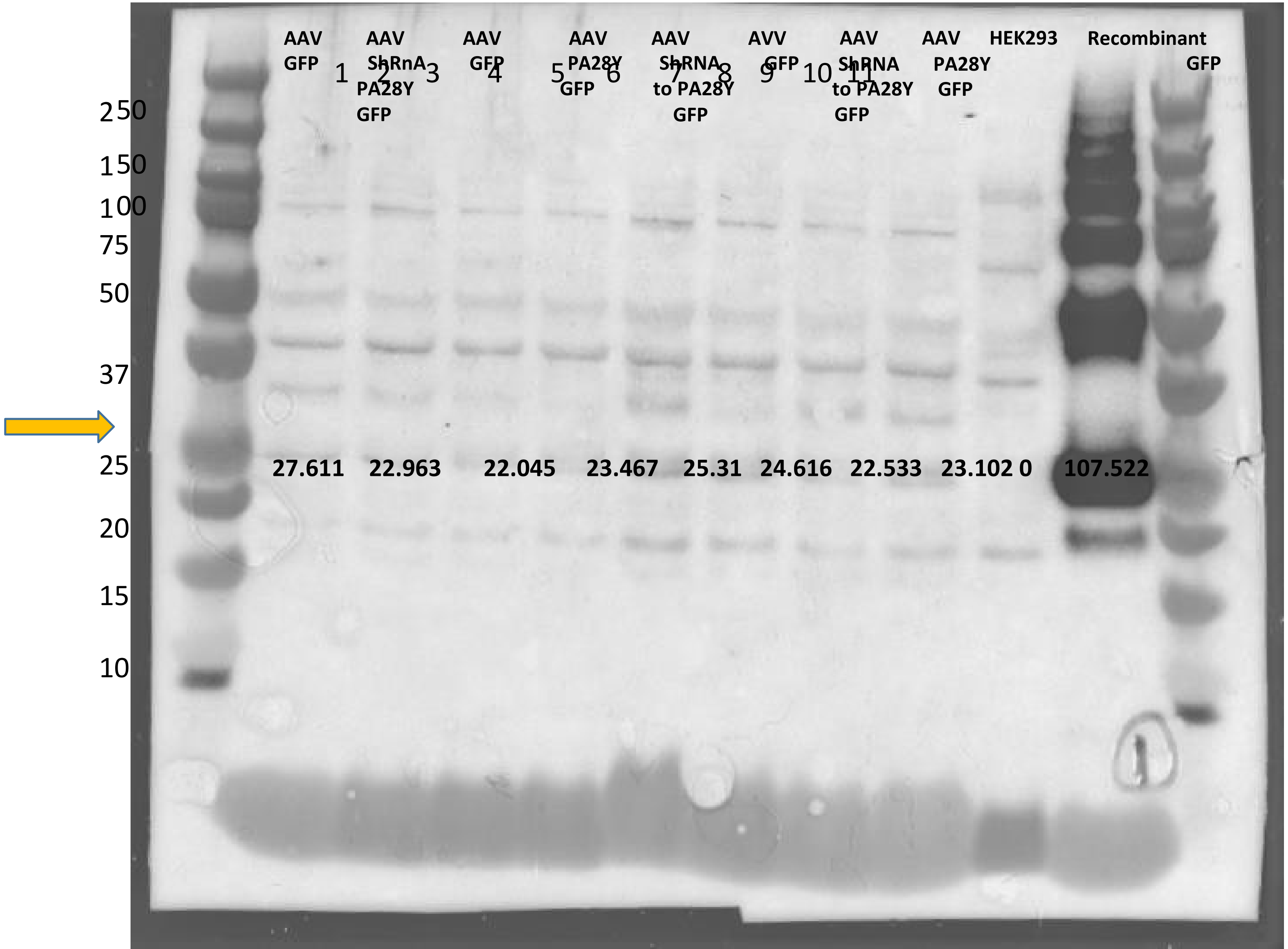

32KD

Protein load: SMC, **50 µg**
Ab . Primary . GFP Bovison . 1:1500

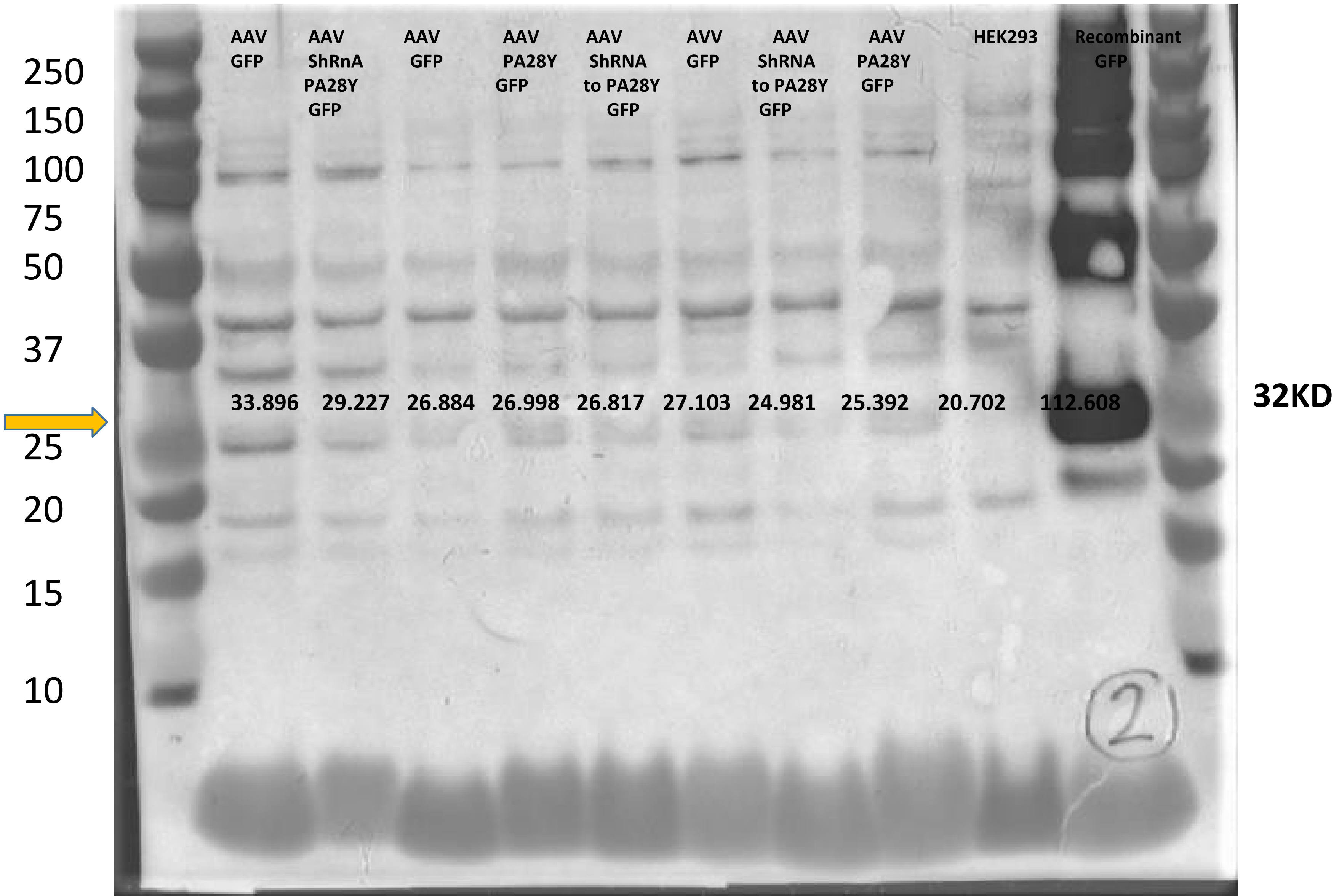

White Matter Supernatant WB sample loaded at concentration **50µg**
Ab . Primary . GFP Bovison . 1:1500 prepared in 5% MILK as recommended incubation over night at 4 `C
Ab . Secondary . Goat anti Rabbit IgG 1: 5000 prepared in 5% Milk incubation 1 H room Temp

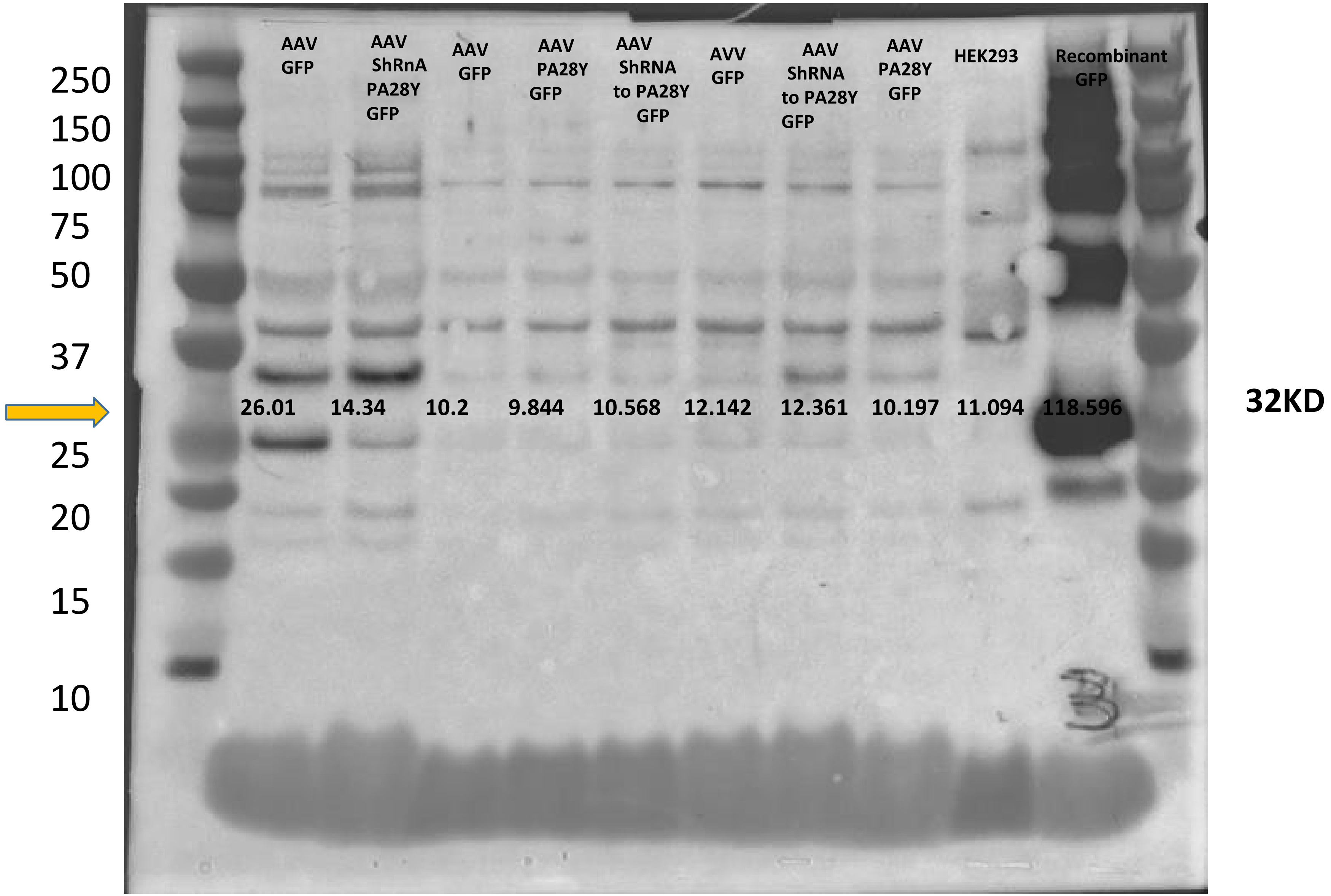

White Matter Supernatant WB sample loaded at concentration **50µg**
Ab . Primary . GFP Biovison . 1:1500 prepared in 5% MILK as recommended incubation over night at 4 `C
Ab . Secondary . Goat anti Rabbit IgG 1: 5000 prepared in 5% Milk incubation 1 H room Temp

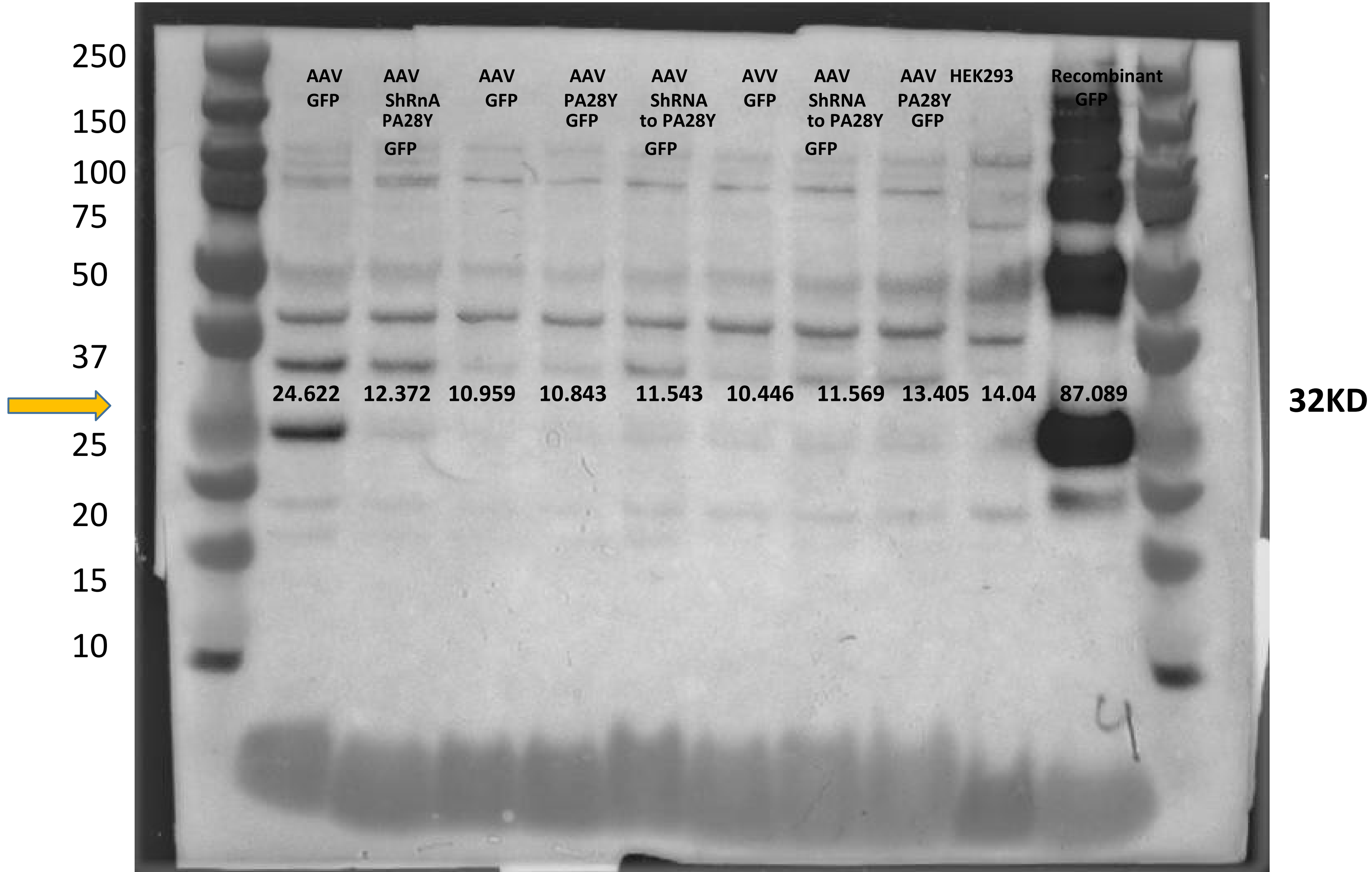

AAV Pigs
PA28gamma

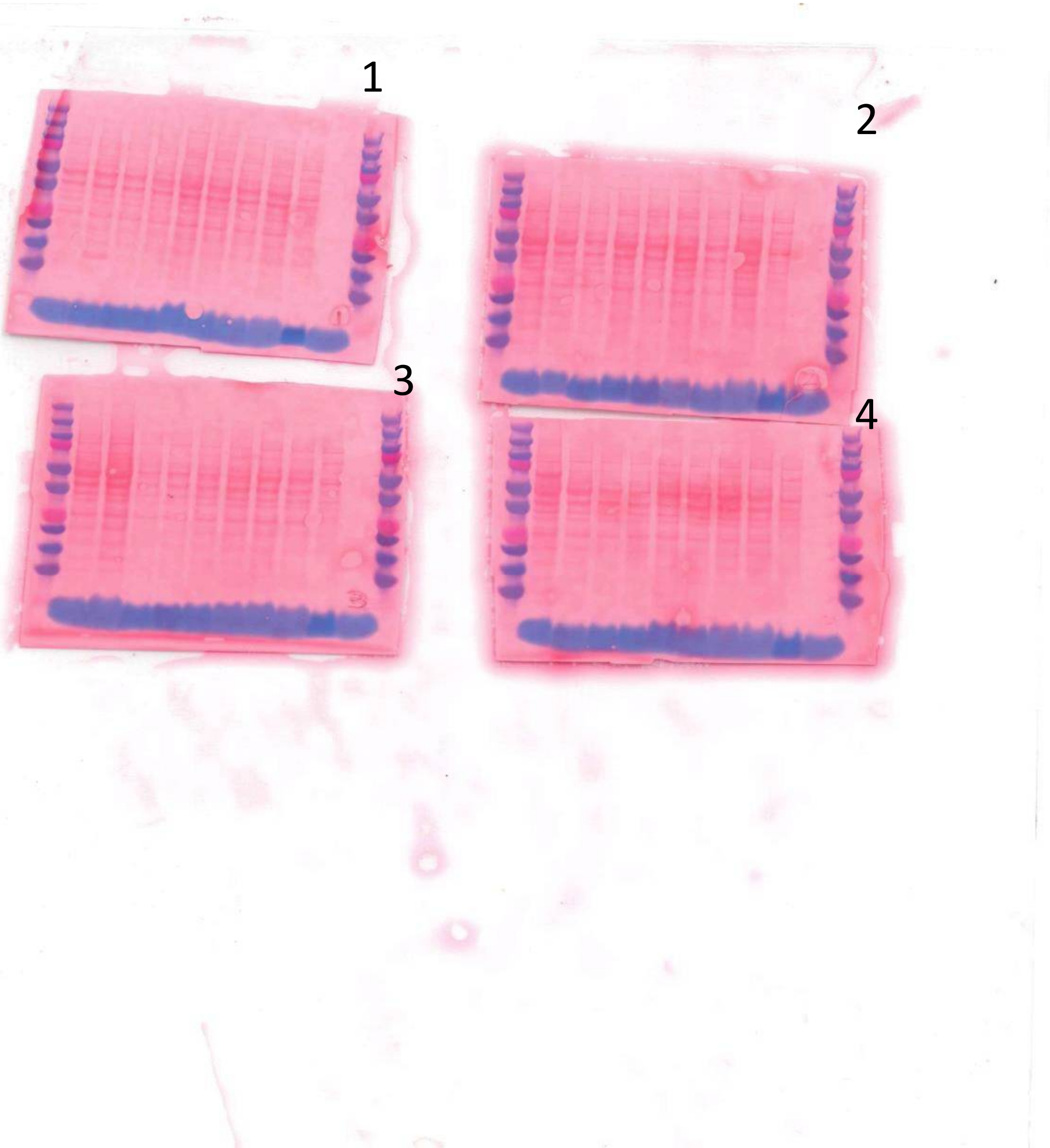
1
2
3
4

Grey Matter Supernatant WB sample loaded at concentration   **50µg**

Ab . Primary . P28 gamma. 1:1000 prepared in 5% Milk as recommended incubation over night at 4`C

Ab . Secondary . Goat anti Rabbit 1: 5000 prepared in 5% Milk   incubation 1 H room Temp

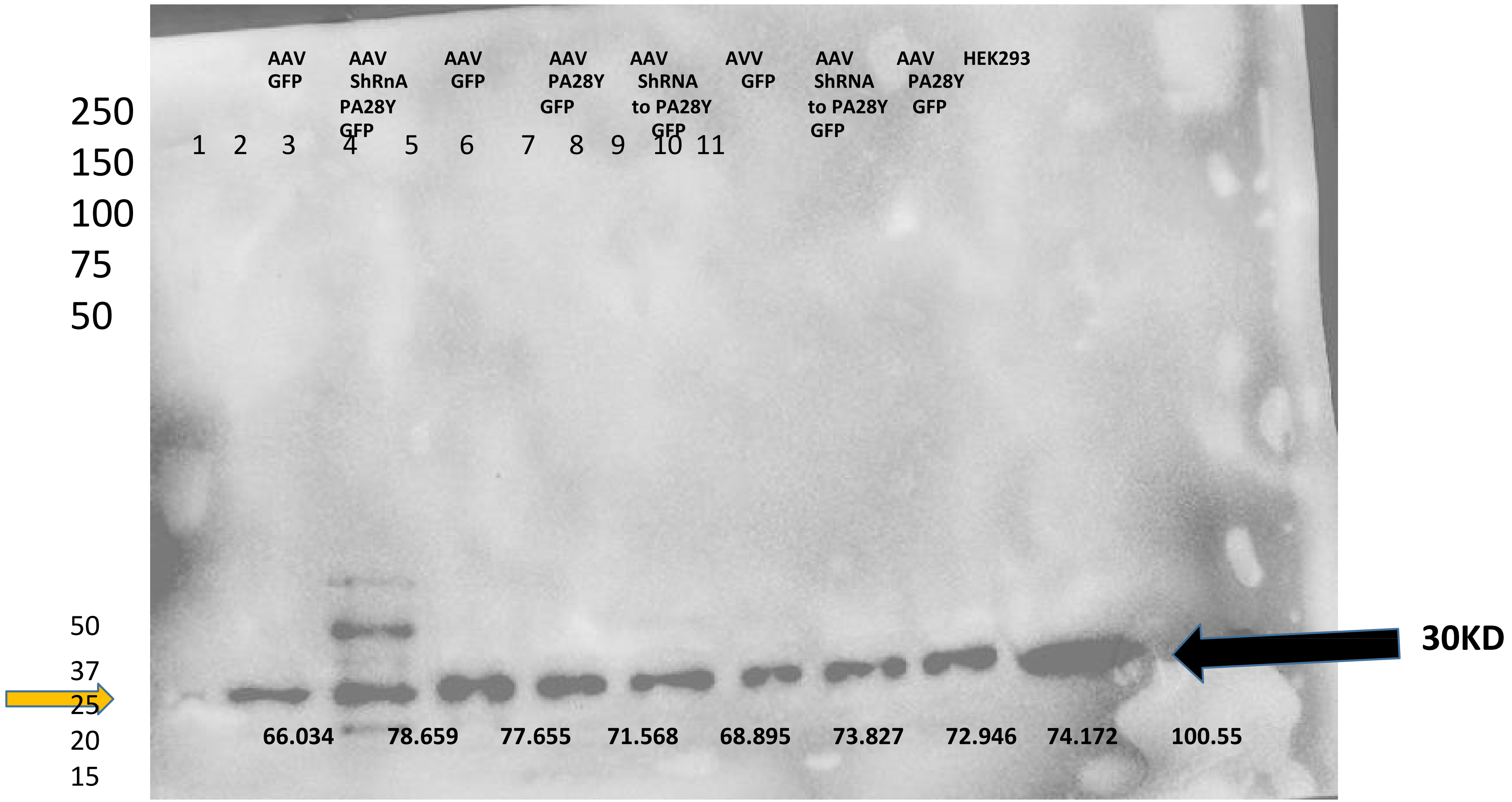

Grey Matter Supernatant WB sample loaded at concentration **50µg**

Ab . Primary . P28 gamma. 1:1000 prepared in 5% Milk as recommended incubation over night at 4`C

Ab . Secondary Goat anti Rabbit 1: 5000 prepared in 5% Milk incubation 1 H room Temp

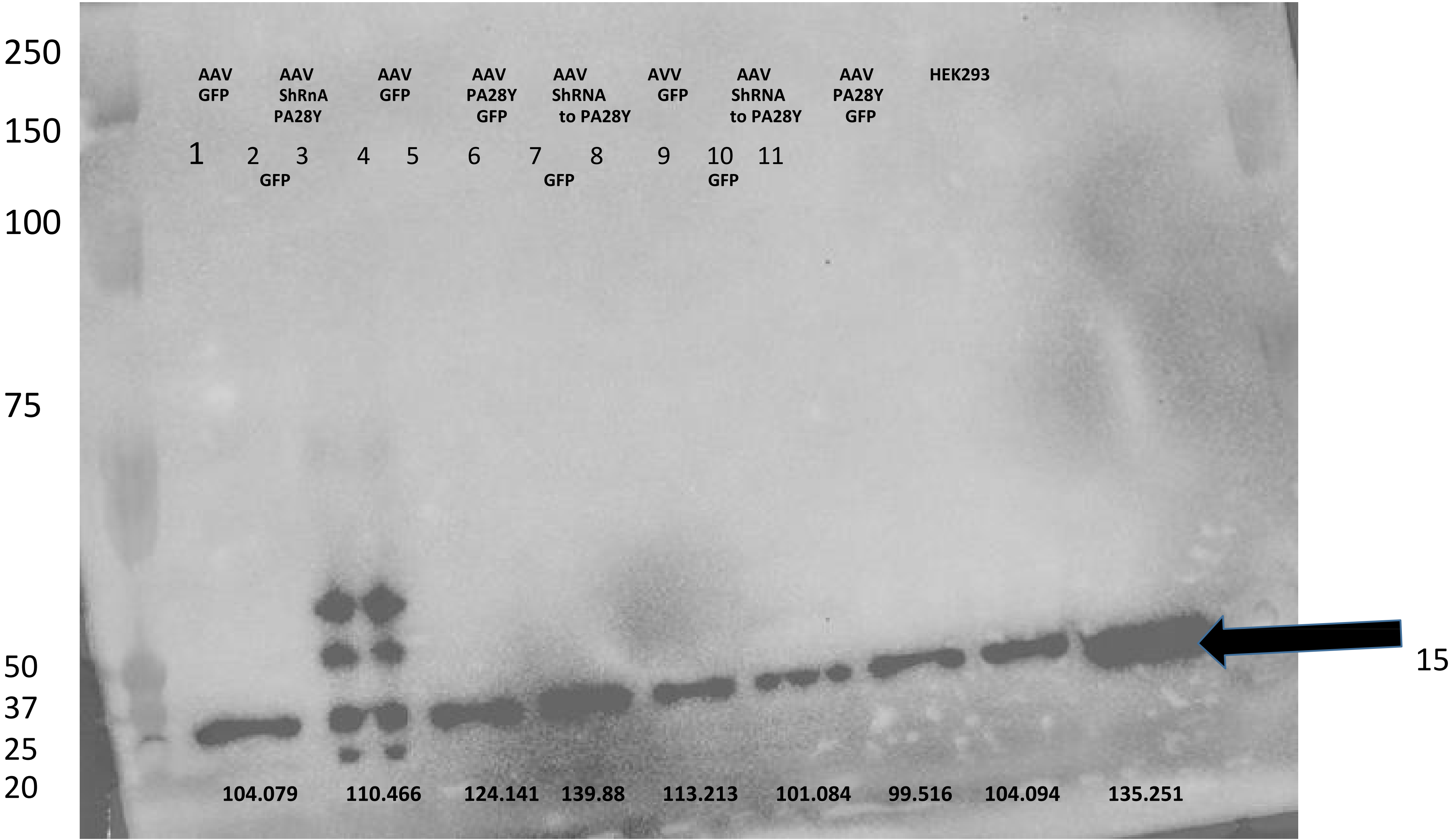

30KD

Protein load: WM, **50 µg**
Ab . Primary P28 gamma . 1:1000

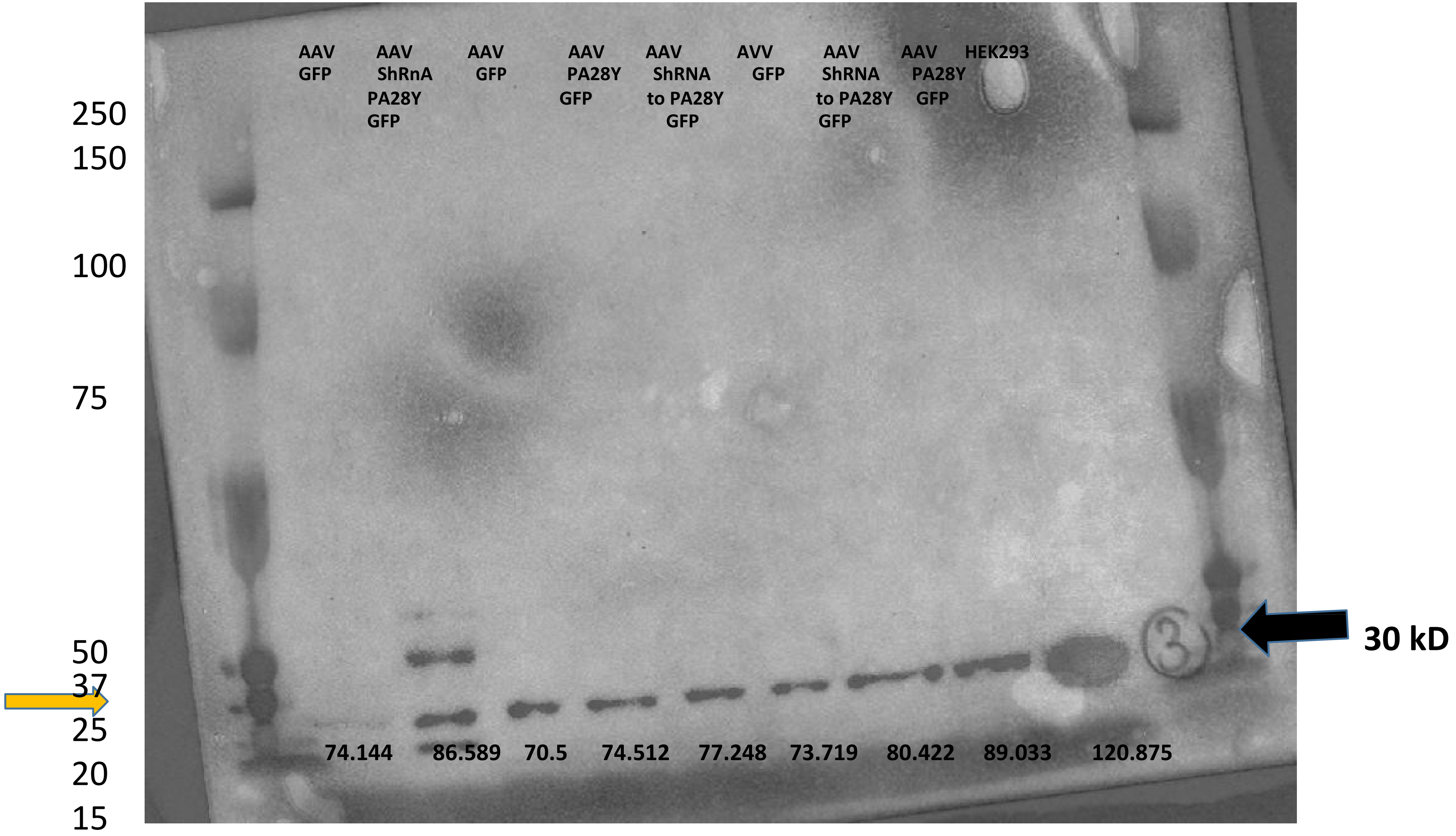

Protein load: WM, **50 µg**

Ab . Primary . P28 gamma. 1:1000

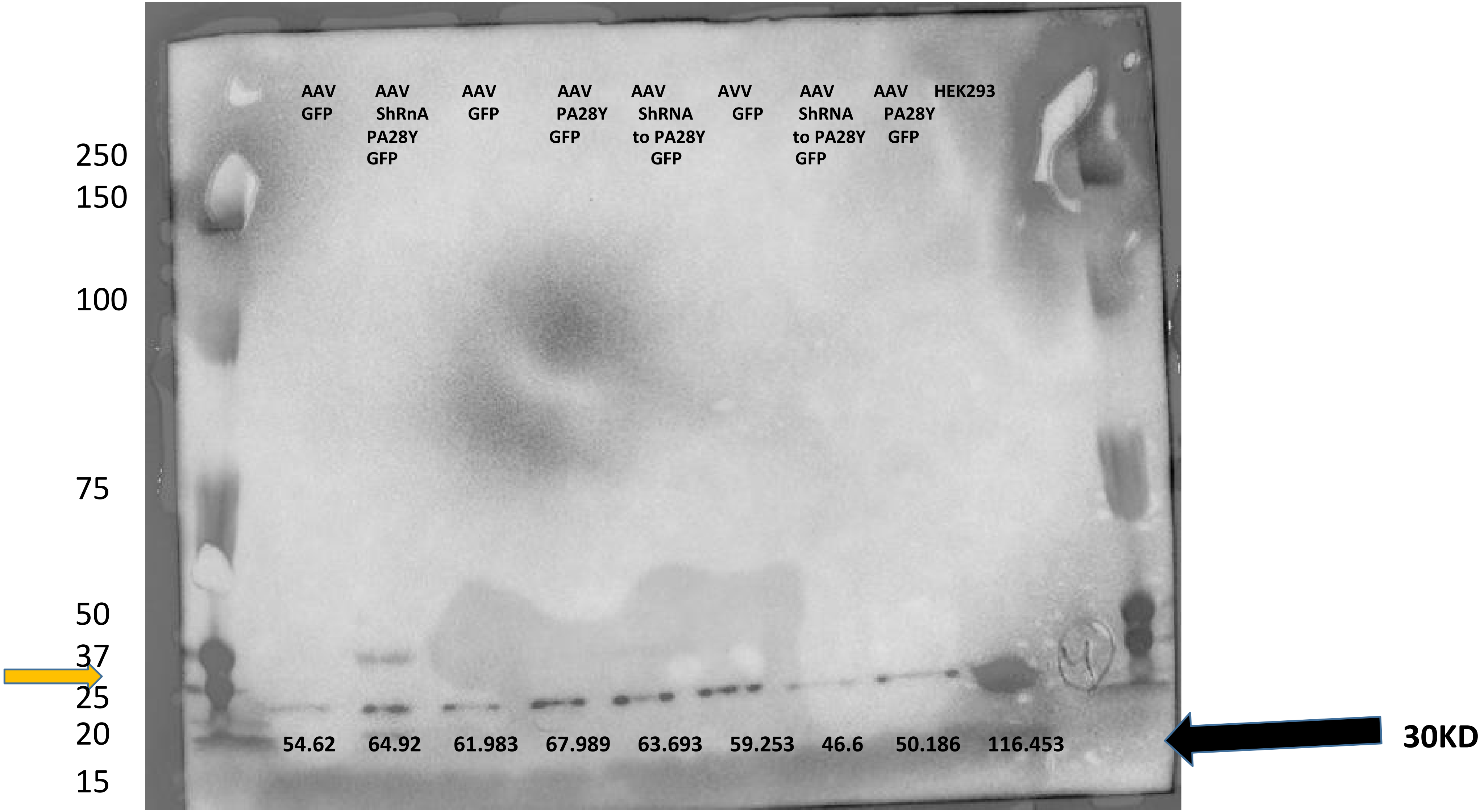

AAV Pigs
PSMB5

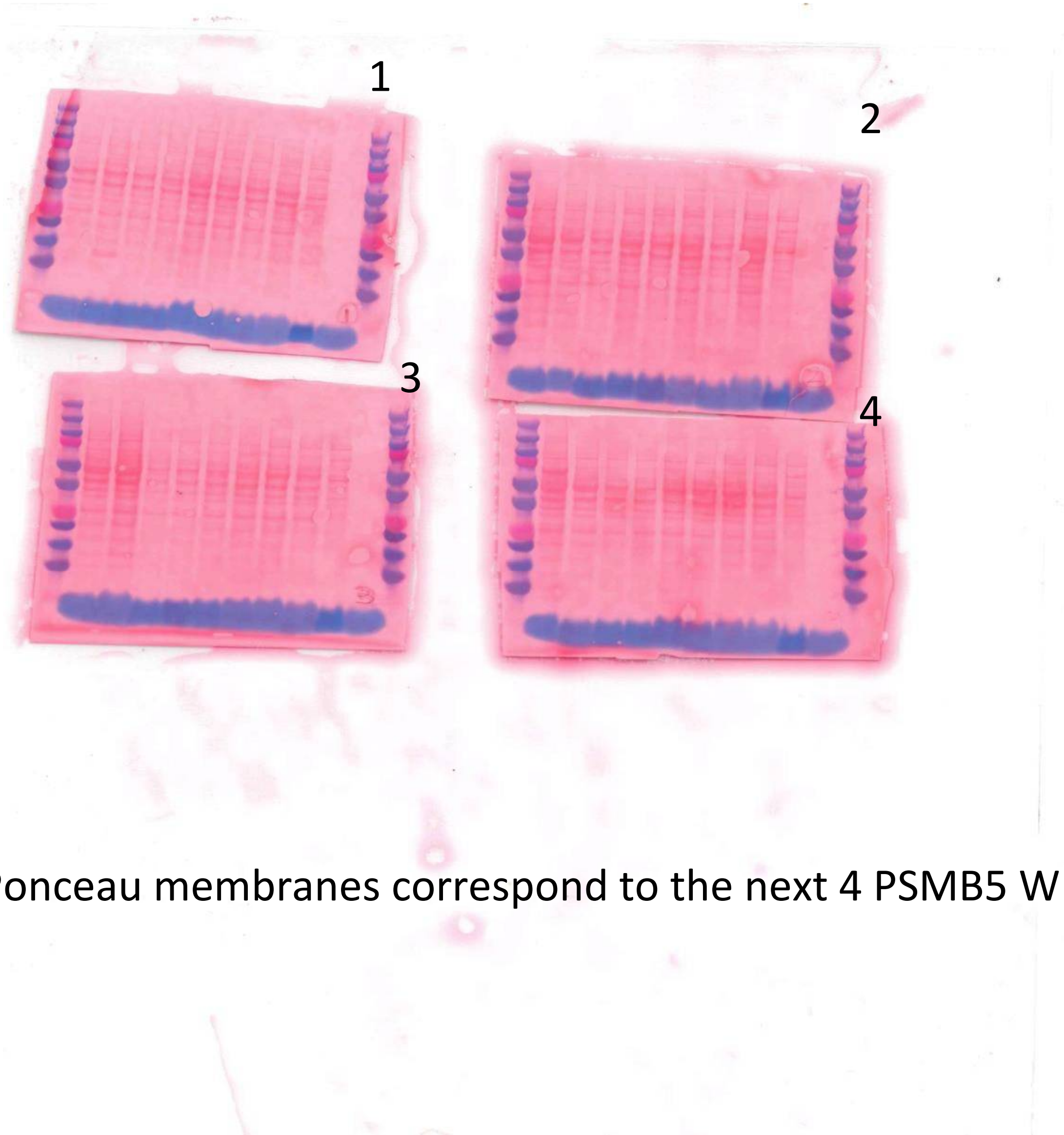

Ponceau membranes correspond to the next 4 PSMB5 WBs.

Protein load: SMC, **50 µg**

Ab . Primary . PSMB5. 1:1000

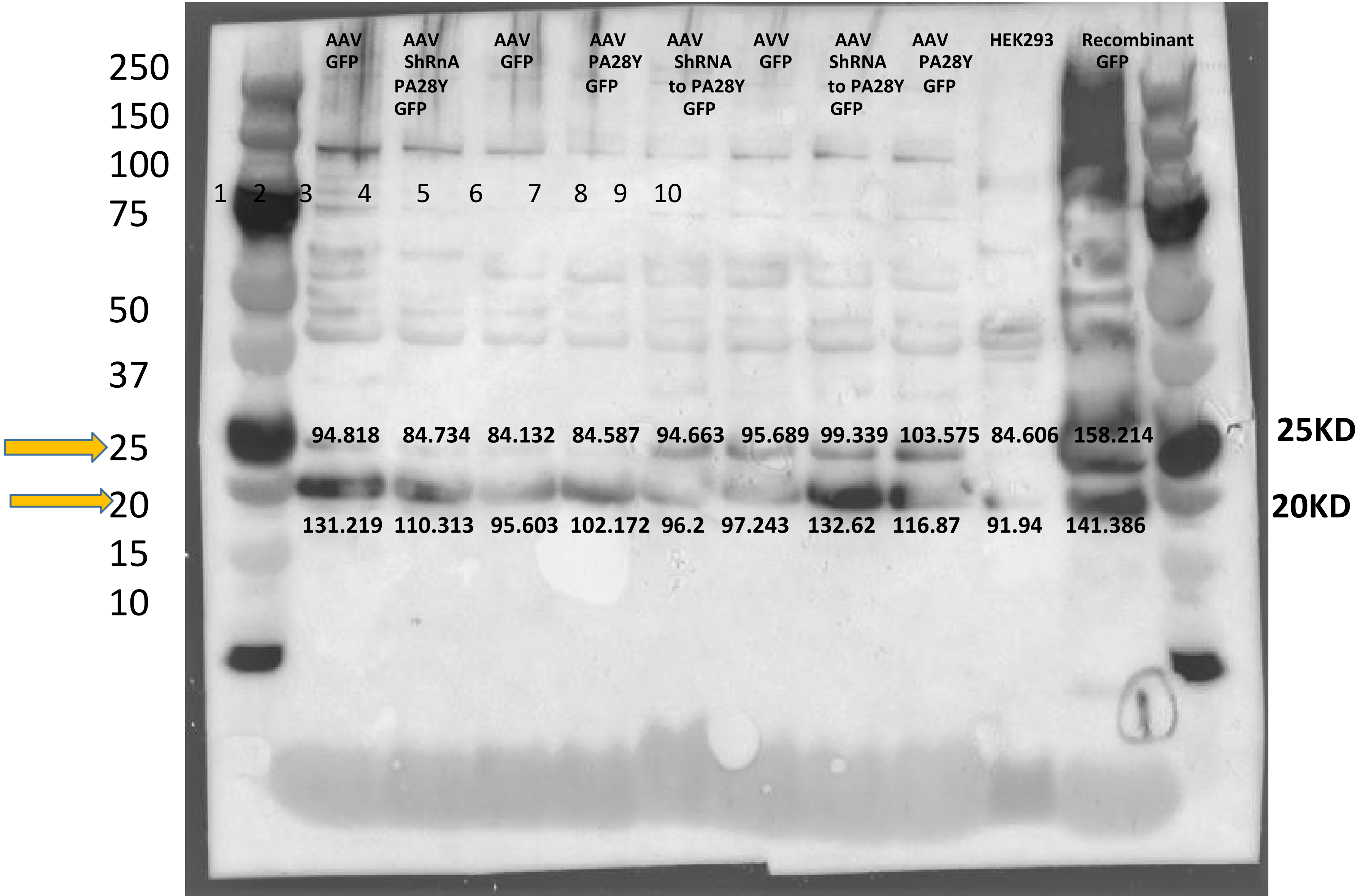

Protein load: SMC, **50 µg**

Ab . Primary . PSMB5. 1:1000

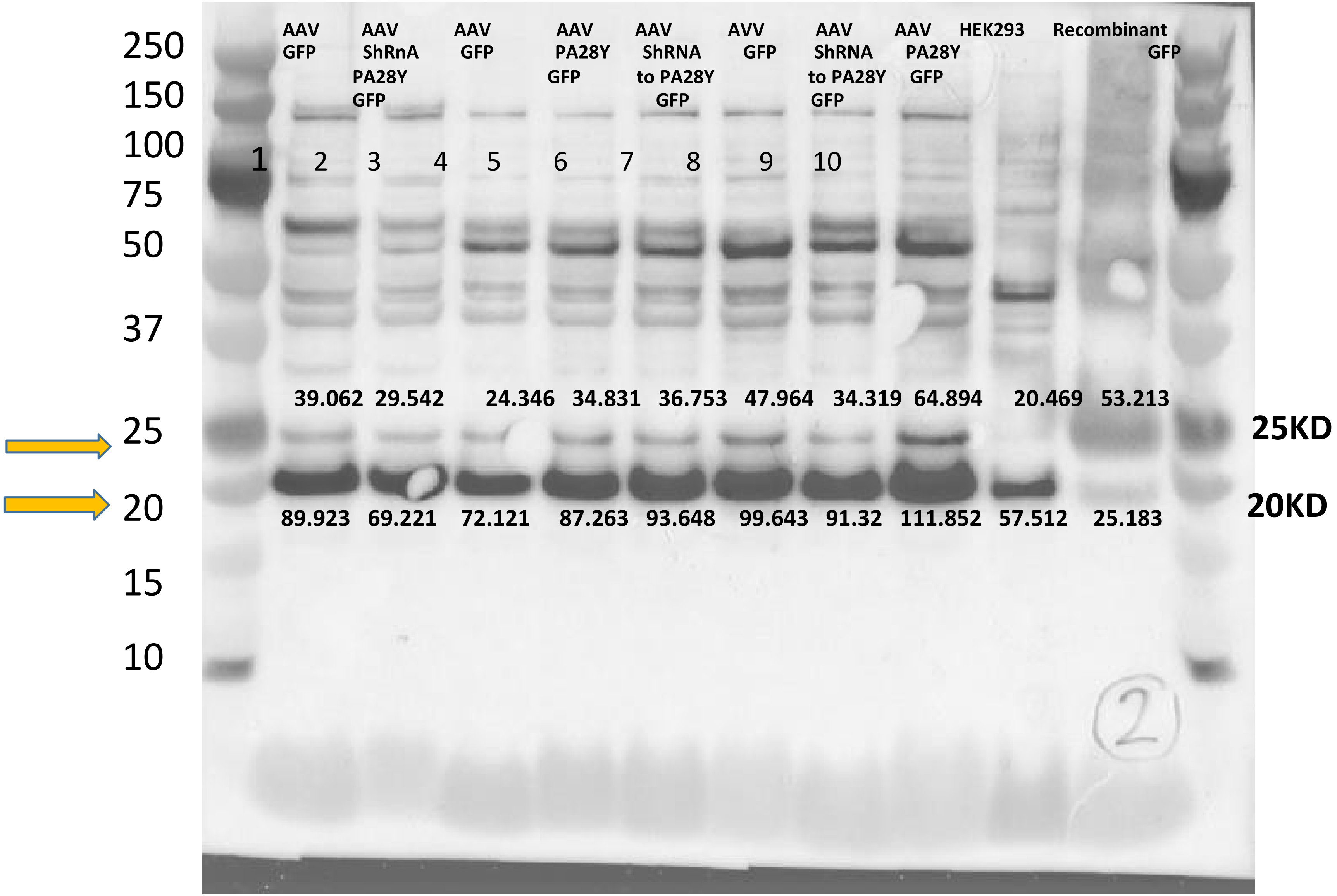

Protein load: WM, **50 µg**

Ab . Primary PSMB5. 1:1000

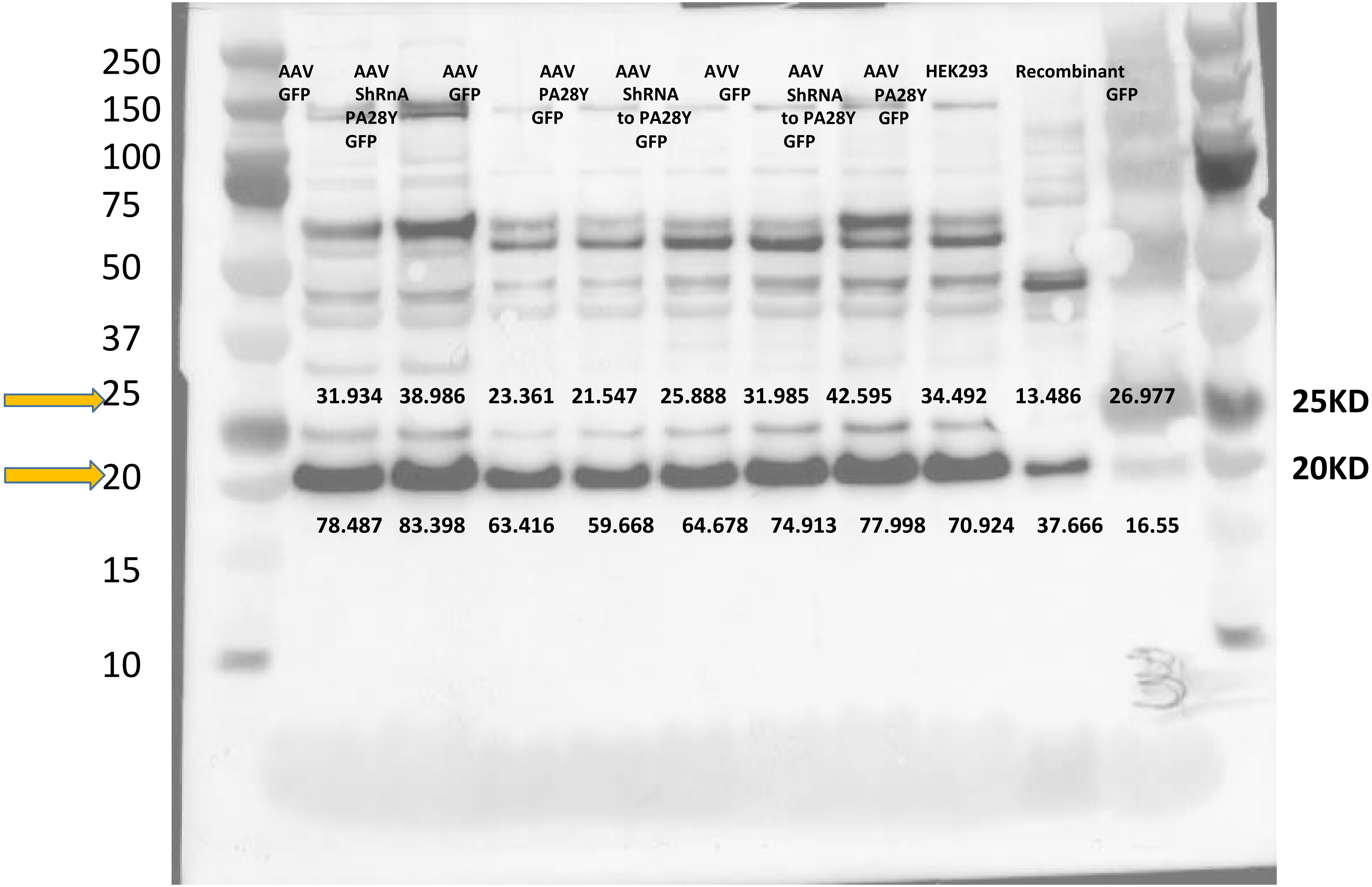

Protein load: WM, **50 µg**
Ab . Primary . PSMB5. 1:1000

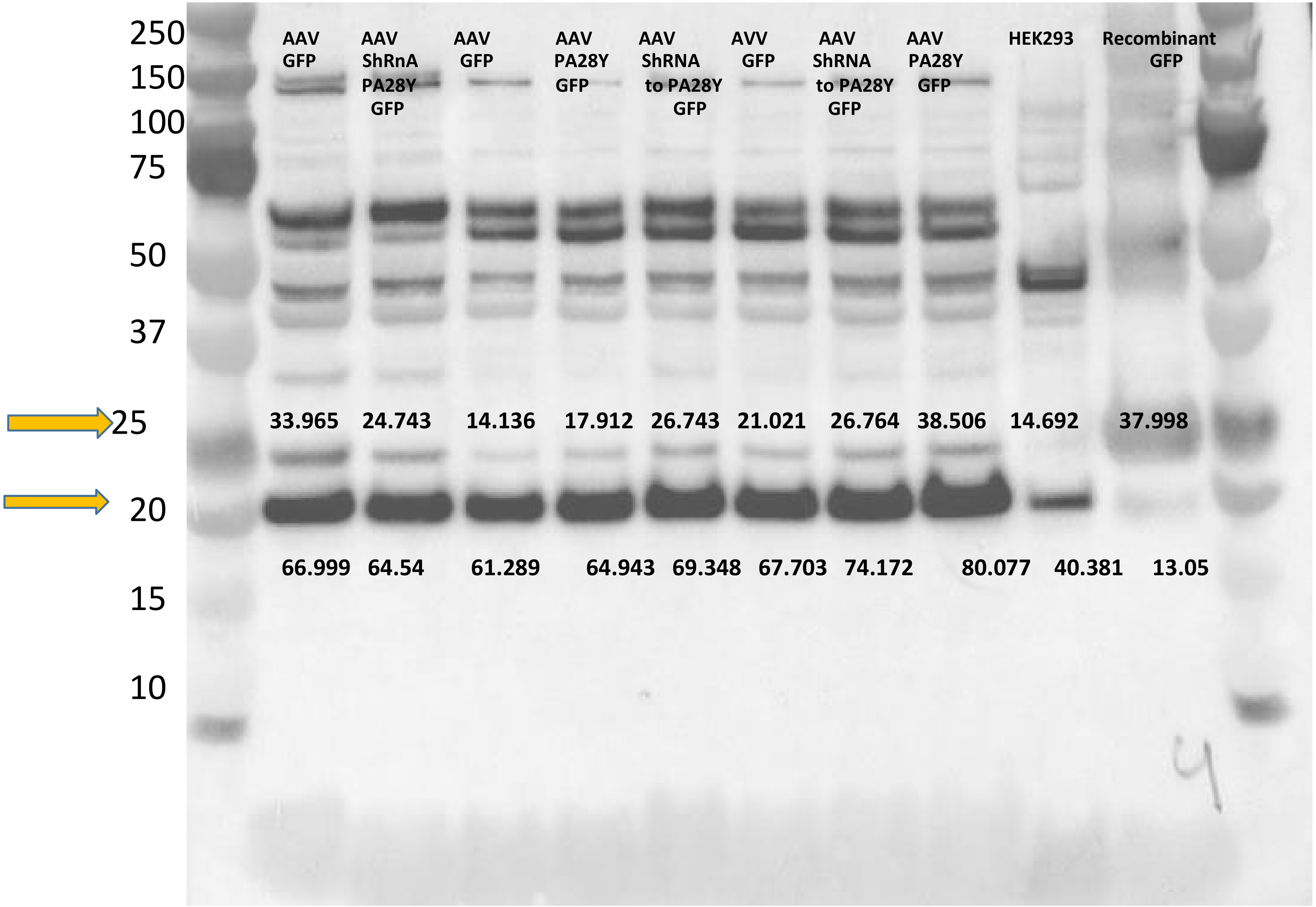

Supplement: Supplementary file 1 [file cells-10-02120-s001.zip › cells-1326434-supplementary.pdf]
